# New Phenol Derivatives from the Haima Cold Seep-Derived Fungus *Aspergillus subversicolor* CYH-17

**DOI:** 10.3390/md22030117

**Published:** 2024-02-29

**Authors:** Yi-Hao Che, Wen-Ping Ding, Zhi-Hui Xiao, Jia-Min Wu, Hao Yin, Fa-Zuo Wang, Si Zhang

**Affiliations:** 1 CAS Key Laboratory of Tropical Marine Bio-Resources and Ecology, Southern Marine Science and Engineering Guangdong Laboratory (Guangzhou), Guangdong Key Laboratory of Marine Materia Medica, RNAM Center for Marine Microbiology, South China Sea Institute of Oceanology, Chinese Academy of Sciences, 164 West Xingang Road, Guangzhou 510301, China; cheyihao1995@163.com (Y.-H.C.); dingwenping19@mails.ucas.ac.cn (W.-P.D.); xzh@scsio.ac.cn (Z.-H.X.); eira3558@163.com (J.-M.W.); yinhao@scsio.ac.cn (H.Y.); 2 University of Chinese Academy of Sciences, 19 Yuquan Road, Beijing 100049, China; 3 Equipment Public Service Center of South China Sea Institute of Oceanology, Chinese Academy of Sciences, 164 West Xingang Road, Guangzhou 510301, China

**Keywords:** cold seep, fungi, phenol derivatives, activity

## Abstract

Seven new phenol derivatives, subversins A–E (**1**–**5**), subversic acid A (**6**) and *epi*-wortmannine G (**7**); one new natural product, 4-hydroxy-7-methoxyphthalide (**8**); and five known compounds (**9**–**13**) were isolated from the fungus *Aspergillus subversicolor* CYH-17 collected from the Haima cold seep. The structures and absolute configurations of these compounds were determined via NMR, MS, optical rotation, electronic circular dichroism (ECD) calculation, X-ray diffraction analysis and comparison with the literature. Compounds **2** and **5** were two pairs of enantiomers. All compounds were tested for their α-glucosidase and acetylcholinesterase (AChE) inhibitory activity, antioxidant activity and antibacterial activity, but no obvious activity was observed among these studied compounds.

## 1. Introduction

Cold seeps have attracted increasing amounts of research interest since they were first discovered in 1983 [1]. In cold seeps, hydrocarbons such as methane, hydrogen sulfide and carbon dioxide are carried to the ocean floor due to geological activity, which leads to abundant chemosynthetic ecosystems [2]. The biological resources found in cold seeps are abundant and include archaea, bacteria, fungi, tubeworms, clams and mussels [3]. Over the past 40 years, most related research has focused on the taxonomy of species [4,5] and the ecological role of microorganisms [6,7]. However, few studies have reported that new secondary metabolites are produced by cold seep-derived creatures [8]. 

In fact, cold seep organisms possess the potential to produce intriguing natural products as they survive in extreme environments [9]. Under extreme conditions, cold seep organisms have evolved unique pathways to produce structurally diverse and biologically active secondary metabolites. According to previous reports, there were a great deal of new compounds that were found in the microorganisms derived from cold seeps, involving alkaloids [10], polyketides [11], terpenoids [12], glycosides [13], macrolides [14] and so forth. The new secondary metabolites displayed significant biological activity, including antimicrobial activity, cytotoxic activity and antioxidant activity. Therefore, cold seeps are a new and significant source for the discovery of active natural products.

**Figure 1:**
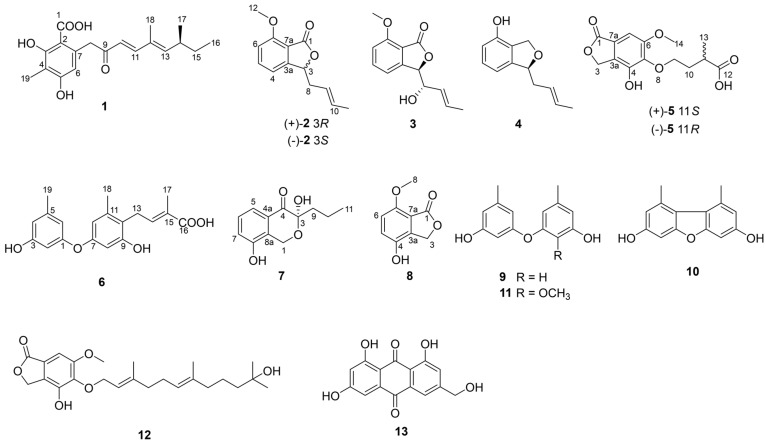
Structures of compounds **1**–**13**.

With the aim of uncovering new secondary metabolites from cold seep-derived fungi, our team carried out a series of works. As a result, we isolated some fungi from the Haima cold seep and discovered several new diketopiperazine alkaloids from the fungi *Aspergillus fumigatus* CYH-5 [15] and *Toxicocladosporium* sp. CYH-18 [16]. Recently, the fungus *Aspergillus subversicolor* CYH-17, isolated from the sediment of the Haima cold seep at a depth of 1363 m in 2021, attracted our attention. Seven new phenol derivatives, subversins A–E (**1**–**5**), subversic acid A (**6**) and epi-wortmannine G (**7**); one new natural product, 4-hydroxy-7-methoxyphthalide (**8**); and five known products, diorcinol (**9**) [17], 3,7-dihydroxy-1,9-dimethyldibenzofuran (**10**) [18], 2-methoxyl cordyol C (**11**) [19], farnesylemefuranone E (**12**) [12] and citreorosein (**13**) [20] (Figure 1), were separated from the fungus *A. subversicolor* CYH-17. NMR, MS, optical rotation, electronic circular dichroism calculation and X-ray diffraction analysis were used to confirm the planar structures and absolute configurations of these compounds. In this study, the separation, structural elucidation and biological activity of those secondary metabolites are reported.

## 2. Results and Discussion

### 2.1. Structural Elucidation

**Figure 2:**
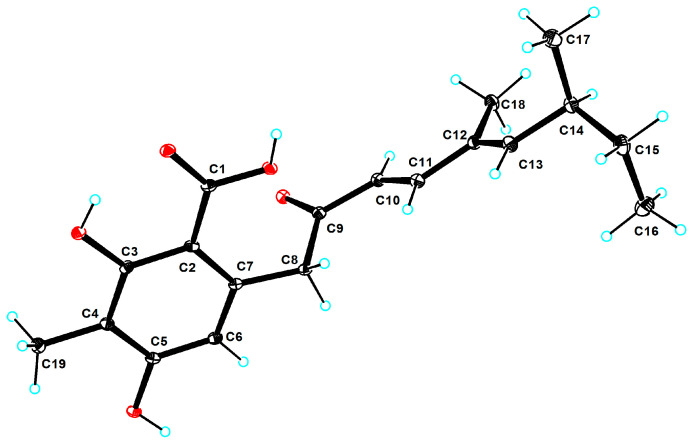
The crystal structure for compound **1**.

Compound **1** was obtained as a colorless crystal with the molecular formula of C_19_H_24_O_5_ based on the molecular ion peak at *m*/*z* 333.1696 [M + H]^+^ (calculated for C_19_H_25_O_5_, 333.1697), suggesting eight degrees of unsaturation. After analyzing detailed NMR data, **1** was found to have a benzoic acid skeleton similar to that of 2,4-dihydroxy-6-(5,7-dimethyl-2-*oxo*-*trans*-3-*trans*-5-nonadienyl)-3-methylbenzaldehyde [21], except for the aldehyde group being a carboxyl group in **1**. The downshift of C-1 (*δ*_C_ 173.23) in **1** and the mass data revealed the difference. The coupling constant (*J*_10,11_ = 15.9 Hz) and the NOESY correlations between H-10 (*δ*_H_ 6.12, d, *J* = 15.9 Hz) and H_3_-18 (*δ*_H_ 1.75, s) and between H-11 (*δ*_H_ 7.22, d, *J* = 15.9 Hz) and H-13 (*δ*_H_ 5.81, d, *J* = 9.7 Hz) suggested that the geometric configurations of the double bonds were determined to be 10*E* and 12*E*. Compared with the optical rotation data of 2,4-dihydroxy-6-(5,7-dimethyl-2-*oxo*-*trans*-3-*trans*-5-nonadienyl)-3-methylbenzaldehyde (${\left[\alpha \right]}_{D}^{23}$ = +51.0 (c 1.0, CHCl_3_)), the absolute configuration of **1** (${\left[\alpha \right]}_{D}^{25}$ = +31.5 (c 0.02, CHCl_3_)) was determined as 14*S*. After repeated crystallization, the crystal of compound **1** was obtained, and the absolute configuration of compound **1** was unambiguously confirmed as 14*S* based on the Cu Kα radiation data with a good flack parameter (−0.01 (8)) (Figure 2). Compound **1** was named subversin A.

**Figure 3:**
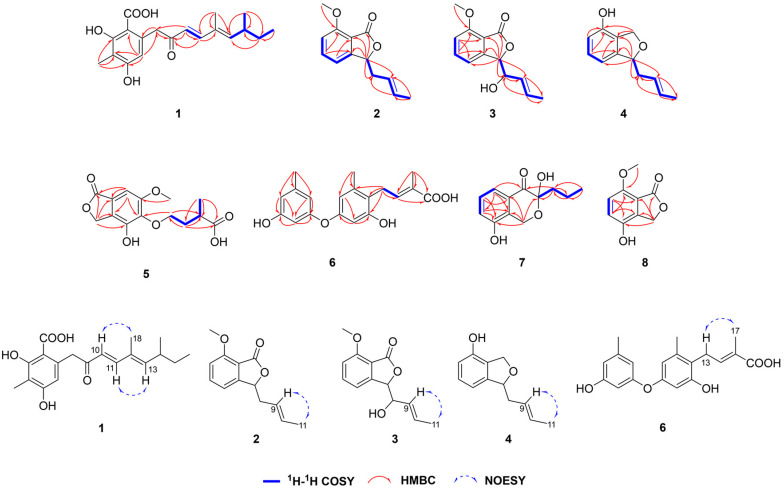
Key ^1^H-^1^H COSY, HMBC and NOESY correlations for compounds **1**–**8**.

Compounds **2**–**3** were obtained as oils with the molecular formulas of C_13_H_14_O_3_ and C_13_H_14_O_4_ on the basis of molecular ion peaks at *m*/*z* 219.1018 [M + H]^+^ (calculated for C_13_H_15_O_3_, 219.1016) and 235.0966 [M + H]^+^ (calculated for C_13_H_15_O_4_, 235.0965), respectively. NMR data showed that **2**-**3** shared the same isobenzofuran-1(3*H*)-one molecular framework. **2** was close to the known compound (*S*)-3-allyl-7-methoxyisobenzofuran-1(3*H*)-one [22], with the exception of the presence of one methyl (*δ*_C_ 18.12/*δ*_H_ 1.61) at the C-10 position in **2**. The above change was proven by the ^1^H-^1^H COSY correlations between H-10/H-11 and the HMBC correlations from H_3_-11 (*δ*_H_ 1.61) to C-9 (*δ*_C_ 124.92) and C-10 (*δ*_C_ 131.17). Compared with **2**, there was a hydroxyl group at the C-8 position in **3.** This was confirmed by the detailed HMBC and ^1^H-^1^H COSY correlations (Figure 3). Thus, the planar structures of **2** and **3** were determined, and **2**-**3** were named subversins B–C. 

The geometric configuration of the double bond of **2**–**3** was confirmed to be 9*E* via the NOESY correlations between H-9 and H_3_-11. Compound **2** was a racemic mixture (${\left[\alpha \right]}_{D}^{25}$ = 0 (c 0.1, CHCl_3_)), which was separated via chiral HPLC into two optically pure enantiomers: (+)-**2** and (−)-**2** (Figure S72). Based on optical rotation data of the (*S*)-3-allyl-7-methoxyisobenzofuran-1(3*H*)-one (${\left[\alpha \right]}_{D}^{34}$ = −43.8 (c 1.0, CHCl_3_)) [22], the absolute structures of (+)-**2** (${\left[\alpha \right]}_{D}^{25}$ = +18.5 (c 0.04, CHCl_3_)) and (−)-2 (${\left[\alpha \right]}_{D}^{25}$ = −26.2 (c 0.05, CHCl_3_)) were defined as 3*R* and 3*S*, respectively. Additionally, the absolute configurations of (+)-**2** and (−)-**2** were confirmed further by ECD calculations, and the calculated ECD spectra for (3*R*)-**2** and (3*S*)-**2** agreed with the experimental curves (Figure S73). The relative configuration of **3** was confirmed as 3R*, 8S* (**3b**) by DP4+ probability analysis using GIAO NMR chemical shift calculations [23] (Table S31). The absolute configuration of **3** was determined through ECD calculation. The calculated ECD spectrum for (3*R*,8*S*)-**3** agreed with the experimental curve (Figure S81).

Compound **4** was yielded as yellow oil with molecular formula of C_12_H_14_O_2_ according to the molecular ion peak at *m*/*z* 191.1074 [M + H]^+^ (calculated for C_12_H_15_O_2_, 191.1067), implying six degrees of unsaturation. According to the NMR data, **4** possessed an isobenzofuran skeleton and was similar to riboxylarinol B [24]**,** and the differences were the presence of one double bond between C-9 and C-10 and the absence of the two hydroxyl groups on C-9 and C-10 in **4**. The ^1^H-^1^H COSY correlations between H-3/H-8/H-9/H-10/H-11, the HMBC correlations from H-9 (*δ*_H_ 5.37–5.52, m) and H-10 (*δ*_H_ 5.59–5.64, m) to C-8 (*δ*_C_ 41.85) and C-11 (*δ*_C_ 18.23) and the mass data indicated the differences. The geometric configuration of the double bond was determined in the same way as **2**. Compared with optical rotation data of the (*S*)-3-deoxyisoochracinic acid [25] $({\left[\alpha \right]}_{D}^{22}$ = −13.0 (c 0.08, MeOH)), the configuration of **4** (${\left[\alpha \right]}_{D}^{25}$ = −24.7 (c 0.08, MeOH)) was defined as 3*S*. Compound **4** was named subversin D.

**Table 1:** ^1^H NMR and ^13^C NMR data of compounds **1**, **5** and **6** (700, 176 MHz, *δ* in ppm, *J* in Hz).

Position	1 ^a^		5 ^b^		6 ^b^	
	*δ*_C_	*δ*_H_	*δ*_C_	*δ*_H_	*δ*_C_	*δ*_H_
1	173.23, C		173.84, C		159.53, C	
2	104.89, C				103.91, CH	6.17 (t, 2.1)
3	162.93, C		69.19, CH_2_	5.22 (s)	160, C	
3a			129.65, C			
4	108.61, C		146.91, C		111.46, CH	6.26 (s)
5	159.10, C		142.04, C		141.57, C	
6	110.94, CH	6.15 (s)	156.63, C		111.71, CH	6.33 (s)
7	136.70, C		99.61, CH	6.94 (s)	157.12, C	
7a			121.58, C			
8	47.47, CH_2_	4.09–4.16 (m)			104.77, CH	6.30 (s)
9	197.23, C		72.84, CH_2_	4.12 (m) 4.03–4.09 (m)	157.35, C	
10	124.45, CH	6.12 (d, 15.9)	35.75, CH_2_	2.00–2.08 (m) 1.81–1.89 (m)	120.99, C	
11	146.61, CH	7.22 (d, 15.9)	39.92, CH	2.68 (m)	139.9, C	
12	131.76, C		184.64, C		113.02, CH	6.30 (s)
13	148.35, CH	5.81 (d, 9.7)	18.46, CH_3_	1.19 (d, 6.8)	26.46, CH_2_	3.48 (d, 7.0)
14	34.37, CH	2.44–2.48 (m)	56.66, CH_3_	3.88 (s)	140.17, CH	6.60 (t, 6.8)
15	29.48, CH_2_	1.36–1.42 (m) 1.24–1.30 (m)			130.37, C	
16	11.84, CH_3_	0.81 (t, 7.4)			175.62, C	
17	20.09, CH_3_	0.96 (d, 6.6)			13.09, CH_3_	1.96 (s)
18	12.29, CH_3_	1.75 (s)			19.99, CH_3_	2.20 (s)
19	8.14, CH_3_	1.94 (s)			21.55, CH_3_	2.21 (s)

^a^ Spectra were measured in DMSO-*d*_6_; ^b^ spectra were measured in methanol-*d*_4_.

**Table 2:** ^1^H NMR and ^13^C NMR data of compounds **2**–**4** (700, 176 MHz, *δ* in ppm, *J* in Hz).

Position	2 ^a^		3 ^a^		4 ^b^	
	*δ*_C_	*δ*_H_	*δ*_C_	*δ*_H_	*δ*_C_	*δ*_H_
1	171.03, C		171.06, C		58.97, CH_2_	4.95 (s)
3	81.58, CH	5.46–5.53 (m)	84.49, CH	5.42 (d, 4.0)	70.89, CH	4.82 (dd, 7.9, 5.3)
3a	154.00, C		151.26, C		142.03, C	
4	115.06, CH	7.10 (d, 7.3)	116.16, CH	7.15 (d, 7.6)	118.13, CH	6.98 (d, 7.7)
5	137.92, CH	7.69 (dd, 8.1, 7.7)	137.57, CH	7.67 (t, 8.0)	129.32, CH	7.20 (t, 7.9)
6	112.03, CH	7.08 (d, 8.0)	112.25, CH	7.09 (d, 8.3)	116.16, CH	6.82 (d, 8.0)
7	159.92, C		159.90, C		156.63, C	
7a	114.35, C		114.97, C		122.95, C	
8	38.31, CH_2_	2.68–2.75 (m) 2.46–2.59 (m)	74.23, CH	4.42–4.45 (m)	41.85, CH_2_	2.38–2.45 (m)
9	124.92, CH	5.25–5.35 (m)	129.15, CH	5.51 (m)	126.79, CH	5.37–5.52 (m)
10	131.17, CH	5.58 (m)	130.49, CH	5.73 (m)	130.04, CH	5.59–5.64 (m)
11	18.12, CH_3_	1.61 (dd, 6.5, 1.4)	17.94, CH_3_	1.68 (m)	18.23, CH_3_	1.70 (d, 6.3)
12	56.33, CH_3_	3.96 (s, 3H)	56.30, CH_3_	3.96 (s)		

^a^ Spectra were measured in methanol-*d*_4_; ^b^ spectra were measured in chloroform-*d*.

Compound **5** was obtained as a yellow oil with the molecular formula of C_14_H_16_O_7_ on the basis of the molecular ion peak at *m*/*z* 295.0829 [M − H]^−^ (calculated for C_14_H_15_O_7_, 295.0823), suggesting seven degrees of unsaturation. The ^1^H and ^13^C NMR data indicated that 5 also had a isobenzofuran-1(3*H*)-one unit, similar to the known compound (+)-5-(3-carboxy-butoxy)-7-hydroxy-4,6-dimethylphthalide [26,27], with the exception of the different substituents on C-4, C-6 and C-7 of the benzene ring in **5**. The differences were proven by the HMBC correlations from H_2_-3 (*δ*_H_ 5.22, s) to C-4 (*δ*_C_ 146.91, s), from H_3_-14 (*δ*_H_ 3.88, s) to C-6 (*δ*_C_ 156.63) and from H-7 (*δ*_H_ 6.94, s) to C-1 (*δ*_C_ 173.84) and C-7a (*δ*_C_ 121.58). Compound **5** was also a racemic mixture (${\left[\alpha \right]}_{D}^{25}$ = 0 (c 0.1, MeOH)), which was separated via chiral HPLC into two optically pure enantiomers: (+)-**5** and (−)-**5** (Figure S74). Based on optical rotation data of the (+)-5-(3-carboxy-butoxy)-7-hydroxy-4,6-dimethylphthalide (${\left[\alpha \right]}_{D}^{25}$ = +6.2 (c 0.36, MeOH)), the absolute structures of (+)-**5** (${\left[\alpha \right]}_{D}^{25}$ = +7.1 (c 0.1, MeOH)) and (−)-**5** (${\left[\alpha \right]}_{D}^{25}$ = −7.7 (c 0.08, MeOH)) were defined as 11*S* and 11*R*, respectively. Compound **5** was named subversin E.

Compound **6** was purified as a yellow oil with the molecular formula of C_19_H_20_O_5_ according to the molecular ion peak at *m*/*z* 329.1393 [M + H]^+^ (calculated for C_19_H_21_O_5_, 329.1384), indicating 10 degrees of unsaturation. The NMR data of **6** were close to those of the known compound verticilatin [28] and the changes were the presence of a carboxyl (*δ*_C_ 175.62) and the absence of one methyl group in **6**. This suggested that the carboxyl group might replace the methyl group in **6**. The above deduction was supported via the HMBC correlations from H-14 (*δ*_H_ 6.60, t, *J* = 6.8 Hz) and H_3_-17 (*δ*_H_ 1.96, s) to C-16 (*δ*_C_ 175.62) and the MS data. The NOESY correlations between H_2_-13 (*δ*_H_ 3.48, d, *J* = 7.0 Hz) and H_3_-17 indicated that the geometric configuration of the double bond was determined as 14*E*. Compound **6** was named subversic acid A.

Compound **7** was a yellow oil with the molecular formula of C_12_H_14_O_4_ based on the molecular ion peak at *m*/*z* 245.0796 [M + Na]^+^ (calculated for C_12_H_14_NaO_4_, 245.0784), implying six degrees of unsaturation. The NMR and the mass data of **7** proved that **7** had the same planar structure as the known compound wortmannine G [29]. However, based on the optical rotation data of wortmannine G (${\left[\alpha \right]}_{D}^{20}$ = +4.0 (c 4 mM, CHCl_3_)), the absolute structure of **7** (${\left[\alpha \right]}_{D}^{25}$ = −5.9 (c 0.1, CHCl_3_)) was defined as 3*R*. Compound **7** was named *epi*-wortmannine G.

Compound **8** was obtained as a white powder with the molecular formula of C_9_H_8_O_4_ on the basis of the molecular ion peak at *m*/*z* 181.0498 [M + H]^+^ (calculated for C_9_H_9_O_4_, 181.0495), suggesting six degrees of unsaturation. The ^1^H-^1^H COSY correlations between H-5/H-6 and the HMBC correlations from H_2_-3 (*δ*_H_ 5.22, s) to C-1 (*δ*_C_ 172.00), C-3a (*δ*_C_ 136.44), C-4 (*δ*_C_ 147.22) and C-7a (*δ*_C_ 114.58), from H-5 (*δ*_H_ 7.05, d, *J* = 8.7 Hz) to C-3a (*δ*_C_ 136.44) and C-7 (*δ*_C_ 152.87), and from H-6 (*δ*_H_ 6.92, d, *J* = 8.7 Hz) to C-4 (*δ*_C_ 147.22) and C-7a (*δ*_C_ 114.58), confirmed the planar structure of compound **8**. Compound **8** was first synthesized by Keay [30] in 1984 and this was the first time that compound **8** had been reported from nature.

**Table 3:** ^1^H NMR and ^13^C NMR data of compounds **7**–**8** (700, 176 MHz, *δ* in ppm, *J* in Hz).

Position	7 ^a^		8 ^a^	
	*δ*_C_	*δ*_H_	*δ*_C_	*δ*_H_
1	58.42, CH_2_	5.03 (d, 16.2) 4.84 (d, 16.2)	172.00, C	
3	97.69, C		68.88, CH_2_	5.22 (s)
3a			136.44, C	
4	193.27, C		147.22, C	
4a	130.64, C			
5	118.59, CH	7.46 (d, 7.6)	123.37, CH	7.05 (d, 8.7)
6	128.87, CH	7.23 (t, 7.9)	113.43, CH	6.92 (d, 8.7)
7	120.56, CH	7.00 (dd, 8.0, 0.7)	152.87, C	
7a			114.58, C	
8	154.19, C		56.53, CH_3_	3.87 (s)
8a	130.29, C			
9	39.75, CH_2_	2.02 (ddd, 13.5, 11.7, 4.8) 1.77 (ddd, 13.6, 11.7, 4.8)		
10	17.61, CH_2_	1.51 (m) 1.31–1.39 (m)		
11	14.76, CH_3_	0.93 (t, 7.4)		

^a^ Spectra were measured in methanol-*d*_4_.

### 2.2. Biological Test

All compounds were tested for antibacterial activity, antioxidant activity, α-glucosidase inhibitory activity and acetylcholinesterase inhibitory activity. Compound **10** displayed inhibitory activity against five Gram-positive bacteria (*B. subtilis*, *E. profundum*, *E. faecalis*, *S. aureus* and MRSA) and one Gram-negative bacterium (*A. baumannii*). Specifically, compound **10** potently inhibited *B. subtilis* with an MIC value of 0.1 μM. No obvious activity of the compounds was observed in terms of antioxidant activity and enzyme inhibitory activity. The IC_50_ and MIC values of the compounds larger than 200 μM were not included in the results of the bioassays (Tables S32 and S33). The structure and activity analysis of **10** and its analogues **6**, **9** and **11** indicated that the dibenzofuran skeleton played an essential role in the antibacterial activity, which was consistent with the literature [31,32]. 

By the end of 2023, over 575 isobenzofuran derivatives had been reported, mainly in Umbelliferae plants and fungi [33,34,35,36,37,38]. Among the isobenzofuran derivatives, 97 originate from marine fungi. Based on the literature [36,37], it was reported that isobenzofuran derivatives exhibited effects on neuroprotective, anti-inflammatory, hepatoprotective and cytotoxicity assays. 

## 3. Materials and Methods

### 3.1. Fungal Materials

The fungus was separated from the sediment (−1363 m) obtained from the Haima cold seep in 2021. The DNA of the fungus was extracted according to the instructions of the DNA extraction kit. Then, a polymerase chain reaction instrument was used to amplify the purified DNA of the fungus with ITS primers (ITS1:5’-CTTGGTCATTTAGAGGAAGTAA-3’; ITS4: 5’-TCCTCCGCTTATTGATATGC-3’). According to the ITS region sequence in the NCBI database, the strain was 99.59% identical to *A. subversicolor* (accession No. NR_135446.1). In terms of the results of the morphological features and the ITS region sequence, the strain was determined to be *A. subversicolor* and was named *A. subversicolor* CYH-17. The fungus was stored in the Research Network for Applied Microbiology (RNAM) Center for Marine Microbiology, South China Sea Institute of Oceanology, Chinese Academy of Sciences.

### 3.2. General

Sephadex LH-20 (GE Healthcare, Stockholm, Sweden) and 100–200 and 200–300 mesh Silica gel (Qingdao Marine Chemical Group Co., Qingdao, China) were used for column chromatography (CC), while an Agilent 1260 HPLC (Agilent Technologies, Santa Clara, CA, USA) equipped with an ODS C-18 column (5 μm, 10 × 250 mm) was used for HPLC separation. In addition, all the data for structural elucidation were collected from an MCP500 automatic polarimeter (AntonPaar, Graz, Austria), UV-2600 spectrometer (Shimadzu, Kyoto, Japan), IR Affinity-1 spectrometer (Shimadzu, Kyoto, Japan), AVANCE IIIHD 700 MHz Digital NMR Spectrometer (Bruker, Billerica, MA, USA), MaXis quadrupole time-of-flight mass spectrometer (Bruker, Mannheim, Germany), Rigaku XtaLAB AFC12 single-crystal diffractometer (Rigaku, Tokyo, Japan) and chirascan circular dichroism spectrometer (Applied Photophysics, Leatherhead, UK).

### 3.3. Fermentation, Extraction and Purification

The fungus was fermented on PDA plates and then transferred to 200 flasks containing medium (rice 100.0 g, artificial sea salt 3.0 g, distilled water 0.1 L). The strain was fermented statically at 25 °C for a month. The rice was extracted with ethyl acetate three times to gather 77.0 g of the ethyl acetate extract. 

The ethyl acetate extract of the fungus was fractionated by silica gel column chromatography (CC) eluted with petroleum ether and ethyl acetate gradient (100:0 to 0:100) to obtain seven fractions (Frs.1–7). Fr.4 was purified via HPLC with MeOH-H_2_O gradient (5:95 to 100:0) to obtain six fractions (Frs.4.1–Fr.4.6). Fr.4.3 was fractionated via silica gel CC eluted with CH_2_Cl_2_-MeOH (100:0 to 90:10) to yield two parts (Frs.4.3.1–Fr.4.3.2). Fr.4.3.1 was purified by HPLC (65% MeOH/H_2_O) to obtain **7** (3.1 mg). Fr.4.3.2 was subjected to Sephadex LH-20 (CH_2_Cl_2_/MeOH = 1:1) to give **4** (1.5 mg). Fr.4.4 was fractionated via silica gel CC eluted with CH_2_Cl_2_-MeOH (100:0 to 90:10) to yield three subfractions Frs.4.4.1–Fr.4.4.3. Fr.4.4.2 was subjected to Sephadex LH-20 (CH_2_Cl_2_/MeOH = 1:1) to give **9** (46.7 mg) and three fractions (Frs.4.4.2.1–4.4.2.3). Fr.4.4.2.1 was purified by HPLC (60% ACN/H2O) to obtain **2** (1.1 mg). Fr.4.4.2.2 was separated by HPLC (60% ACN/H_2_O) to yield **11** (1.8 mg). Fr.4.4.2.3 was fractionated by HPLC (60% ACN/H_2_O) to give **10** (3.9 mg). Fr.4.4.3 was subjected to Sephadex LH-20 (CH_2_Cl_2_/MeOH = 1:1) to obtain Fr.4.4.3.1. Fr.4.4.3.1 was separated via silica gel CC eluted with CH_2_Cl_2_-MeOH (100:0 to 90:10) to yield **13** (2.2 mg) and **6** (3.1 mg). Fr.4.5 was fractionated via silica gel CC eluted with CH_2_Cl_2_-MeOH (100:0 to 95:5) to give the two parts Fr.4.5.1–Fr.4.5.2. Fr.4.5.1 was purified by HPLC (70% ACN/H_2_O) to obtain **12** (3.0 mg). Fr.4.5.2 was fractionated via Sephadex LH-20 (CH_2_Cl_2_/MeOH = 1:1) to yield **1** (6.0 mg). Fr.5 was separated via HPLC with MeOH-H_2_O gradient (5:95 to 100:0) to obtain two fractions (Frs.5.1–Fr.5.2). Fr.5.2 was subjected to Sephadex LH-20 (CH_2_Cl_2_/MeOH = 1:1) to give Fr.5.2.1–Fr.5.2.2. Fr.5.2.1 was purified by HPLC (43% MeOH/H_2_O) to obtain **3** (2.2 mg). Fr.5.2.2 was fractionated by HPLC (55% MeOH/H_2_O) to obtain **8** (1.2 mg). Fr.6 was separated via HPLC with MeOH-H_2_O gradient (5:95 to 100:0) to yield three fractions (Frs.6.1–Fr.6.3). Fr.6.2 was subjected to Sephadex LH-20 (MeOH) to give Fr.6.2.1. Fr.6.2.1 was purified by HPLC (37% ACN/H_2_O) to obtain **5** (1.5 mg).

#### 3.3.1. Subversin A (**1**)

Colorless crystal; ${\left[\alpha \right]}_{D}^{25}$ = +41.1 (c 0.1, MeOH), ${\left[\alpha \right]}_{D}^{25}$ = +31.5 (c 0.02, CHCl_3_); HR-ESI-MS at *m*/*z* 333.1696 [M + H]^+^ (calculated for C_19_H_25_O_5_, 333.1697); UV (MeOH) *λ*_max_ (log *ε*) 219 (4.38), 279 (4.33) nm; IR (film) ν_max_ 3340, 2949, 1647, 1018, 671 cm^−1^; ^1^H NMR and ^13^C NMR data, see Table 1. 

#### 3.3.2. Subversin B (**2**)

Colorless oil; ${\left[\alpha \right]}_{D}^{25}$ = 0 (c 0.1, CHCl_3_); HR-ESI-MS at *m*/*z* 219.1018 [M + H]^+^ (calculated for C_13_H_15_O_3_, 219.1016); UV (MeOH) *λ*_max_ (log *ε*) 215 (4.12), 235 (3.94) 298 (3.74) nm; IR (film) ν_max_ 2926, 1759, 1607,1298,1038,787 cm^−1^; ^1^H NMR and ^13^C NMR data, see Table 2.

(+)-2. Colorless oil; ${\left[\alpha \right]}_{D}^{25}$ = +18.5 (c 0.04, CHCl_3_); CD (MeOH): 209 nm (Δ*ε* = 2.94), 240 nm (Δ*ε* = −1.11), 296 nm (Δ*ε* = −0.65).

(−)-2. Colorless oil; ${\left[\alpha \right]}_{D}^{25}$ = −26.2 (c 0.05, CHCl_3_); CD (MeOH): 210 nm (Δ*ε* = −2.33), 241 nm (Δ*ε* = 1.06), 296 nm (Δ*ε* = 0.66).

#### 3.3.3. Subversin C (**3**)

Colorless oil; ${\left[\alpha \right]}_{D}^{25}$ = +6.4 (c 0.1, MeOH); HR-ESI-MS at *m*/*z* 235.0966 [M + H]^+^ (calculated for C_13_H_15_O_4_, 235.0965); UV (MeOH) *λ*_max_ (log *ε*) 213 (4.13), 236 (3.84) 299 (3.64) nm; IR (film) ν_max_ 3385, 2954, 1744, 1026, 689 cm^−1^; CD (MeOH): 209 nm (Δ*ε* = −9.85), 243 nm (Δ*ε* = 2.31), 298 nm (Δ*ε* = 1.25); ^1^H NMR and ^13^C NMR data, see Table 2.

#### 3.3.4. Subversin D (**4**)

Yellow oil; ${\left[\alpha \right]}_{D}^{25}$ = −17 (c 0.1, CHCl_3_), ${\left[\alpha \right]}_{D}^{25}$ = −24.7 (c 0.08, MeOH); HR-ESI-MS at *m*/*z* 191.1074 [M + H]^+^ (calculated for C_12_H_15_O_2_, 191.1067); UV (MeOH) *λ*_max_ (log *ε*) 220 (3.72), 281 (3.31) nm; IR (film) ν_max_ 3327, 2945,1018, 671 cm^−1^; ^1^H NMR and ^13^C NMR data, see Table 2.

#### 3.3.5. Subversin E (**5**)

Yellow oil; ${\left[\alpha \right]}_{D}^{25}$ = 0 (c 0.1, MeOH); HR-ESI-MS at *m*/*z* 295.0829 [M − H]^−^ (calculated for C_14_H_15_O_7_, 295.0823); UV (MeOH) *λ*_max_ (log *ε*) 216 (4.21), 266 (3.65) nm; IR (film) ν_max_ 3319, 2947, 1651, 1018, 675 cm^−1^; ^1^H NMR and ^13^C NMR data, see Table 1. 

(+)-5. Yellow oil; ${\left[\alpha \right]}_{D}^{25}$ = +7.1 (c 0.1, MeOH); CD (MeOH): 223 nm (Δ*ε* = 0.30).

(−)-5. Yellow oil; ${\left[\alpha \right]}_{D}^{25}$ = −7.7 (c 0.08, MeOH); CD (MeOH): 224 nm (Δ*ε* = −0.39). 

#### 3.3.6. Subversic Acid A (**6**)

Yellow oil; HR-ESI-MS at *m*/*z* 329.1393 [M + H]^+^ (calculated for C_19_H_21_O_5_, 329.1384); UV (MeOH) *λ*_max_ (log *ε*) 219 (4.61), 282 (3.73) nm; IR (film) ν_max_ 3364, 1595, 1522, 1153, 837, 677 cm^−1^; ^1^H NMR and ^13^C NMR data, see Table 1. 

#### 3.3.7. epi-Wortmannine G (**7**)

Yellow oil; ${\left[\alpha \right]}_{D}^{25}$ = −5.9 (c 0.1, CHCl_3_); HR-ESI-MS at *m*/*z* 245.0796 [M + Na]^+^ (calculated for C_12_H_14_NaO_4_, 245.0784); UV (MeOH) *λ*_max_ (log *ε*) 223 (4.18), 260 (3.86) 318 (3.41) nm; IR (film) ν_max_ 3334, 2954, 1690,1020, 754, 677 cm^−1^; ^1^H NMR and ^13^C NMR data, see Table 3. 

#### 3.3.8. 4-Hydroxy-7-methoxyphthalide (**8**)

White powder; HR-ESI-MS at *m*/*z* 181.0498 [M + H]^+^ (calculated for C_9_H_9_O_4_, 181.0495); UV (MeOH) *λ*_max_ (log *ε*) 217 (3.96), 237 (3.57) 324 (3.37) nm; IR (film) ν_max_ 3343, 2947, 1649, 1018, 669 cm^−1^; ^1^H NMR and ^13^C NMR data, see Table 3. 

### 3.4. X-ray Crystal Structure Analysis

Crystallographic data of compound **1** were yielded on a Rigaku XtaLAB AFC12 single-crystal diffractometer (Rigaku, Japan) via Cu Kα radiation. The crystal was kept at 100.5 (9) K during the data collection. Using Olex2, the structure was solved with the SHELXT structure solution program using Intrinsic Phasing and refined with the SHELXL refinement package using Least Squares minimization. The crystallographic data of compound **1** were stored in the Cambridge Crystallographic Data Centre database (deposition numbers 2324024). Copies of the data are available free of charge from the CCDC at www.ccdc.cam.ac.uk, accessed on 6 March 2022.

Crystal data for compound **1**: C_38_H_48_O_10_, *M* = 664.76, triclinic, space group P1 (no.1), *a* = 6.32630 (10) Å, *b* = 10.8354 (2) Å, *c* = 13.0039 (2) Å, *α* = 91.6160(10)°, *β* = 103.8220 (10)°, *γ* = 92.5990 (10)°, *V* = 863.99 (3) Å^3^, *Z* = 1, *T* = 100.5 (9) K, μ (Cu K*α*) = 0.751 mm^−1^, *Dcalc* = 1.278 g/cm^3^, 17397 reflections measured (7.006° ≤ 2Θ ≤ 148.54°), 6466 unique (*R*_int_ = 0.0298, *R*_sigma_ = 0.0318) which were used in all calculations. The final *R*_1_ was 0.0340 (I > 2σ (I)) and *wR*_2_ was 0.0928 (all data). The goodness of fit on F^2^ was 1.079. Flack parameter = −0.01 (8), melting point: 184.0–185.0 °C.

### 3.5. Bioassays

#### 3.5.1. Antibacterial Assay

The bacteria *Vibrio alginolyticus* XSBZ14, *Enterococcus faecalis* ATCC 29212, *Acinetobacter baumannii* ATCC 19606, *Escherichia coli* ATCC 25922, *Bacillus subtilis* BS01, *Klebsiella pneumoniae* ATCC 13883, *Exiguobacterium profundum Staphylococcus aureus* ATCC 29213 and MRSA 107352 were used to measure antibacterial activity. The procedures of the antibacterial test were the same as Zhang [39]. Ciprofloxacin was used as the positive control.

#### 3.5.2. AChE Inhibitory Assay

The procedures of the AChE inhibitory test were the same as Yang [40]. Tacrine was used as the positive control.

#### 3.5.3. α-Glucosidase Inhibitory Assay

The procedures of the α-glucosidase inhibitory test were the same as Ding [41]. Acarbose was used as the positive control.

#### 3.5.4. DPPH Radical Scavenging Assay

The procedures of the DPPH radical scavenging test were the same as Zhong [42]. Ascorbic acid was used as the positive control.

### 3.6. Chiral HPLC Separation of Compounds **2** and **5**

Chiral HPLC separations of the compounds were recorded on the HPLC (Agilent 1260) equipped with CHIRALPAK^®^ IA (250 × 4.6 mm, 5 μm). A phase: hexane with 0.1% formic acid; B phase: isopropanol.

Compound **2**: gradient program: 0 min (93%A–7%B) to 25 min (93%A–7%B); flow rate: 1 mL/min; detection: UV 215 nm. 

Compound **5**: gradient program: 0 min (80%A–20%B) to 12 min (80%A–20%B); flow rate: 1 mL/min; detection: UV 215 nm.

## 4. Conclusions

Chemical exploration of the fungus *Aspergillus subversicolor* CYH-17 resulted in the isolation and elucidation of seven new phenol derivatives, subversins A–E (**1**–**5**), subversic acid A (**6**) and *epi*-wortmannine G (**7**); one new natural product, 4-hydroxy-7-methoxyphthalide (**8**); and five known secondary metabolites (**9**–**13**). The structural frameworks of the compounds included benzoic acid, isobenzofuran-1(3*H*)-one, isobenzofuran and isochroman-4-one. Compound **10** inhibited six bacteria with MIC values ranging from 0.1 to 50 μM. No obvious activity of the compounds was seen in the enzyme inhibitory activity and antioxidant activity. Future research should focus on exploring the diverse structures of dibenzofuran and isobenzofuran derivatives through OSMAC strategies and elucidating the structure–activity relationship of the compounds in this fungus.

## Data Availability

Data is contained within the article or Supplementary Material.

## Supplementary Materials

The following supporting information can be downloaded at: https://www.mdpi.com/article/10.3390/md22030117/s1, HR-ESI-MS, ^1^H NMR, ^13^C NMR and DEPT 135°, HSQC, HMBC, ^1^H-^1^H COSY, NOESY, UV, IR spectra of compounds **1**–**8** (Figures S1–S71); the chiral HPLC separation profile **2** and **5** and the ECD calculation and results of **2** (Figures S72–S75, Table S1); the computational methods and results of **3** (Figures S76–S81 and Tables S2–S31); the ITS sequence of *A. subversicolor* CYH-17 and the antibacterial activity, α-glucosidase inhibitory activity and antioxidant activity of the compounds (Tables S32–S33) [43,44,45].
