# Supplementary material for: New Phenol Derivatives from the Haima Cold Seep-Derived Fungus Aspergillus subversicolor CYH-17
Source: Mar Drugs. 2024 Feb 29;22(3):117. doi: 10.3390/md22030117 (PMC10971832; doi:10.3390/md22030117)
Supplement: Supplementary file 1 [file marinedrugs-22-00117-s001.zip › marinedrugs-2871816-supplementary.pdf]

Supplementary Materials

New Phenol Derivatives from Haima the Cold Seep-derived Fungus *Aspergillus subversicolor* CYH-17

Yi-Hao Che [1,2], Wen-Ping Ding [1], Zhi-Hui Xiao [1,3], Jia-Min Wu [1,2], Hao Yin [1], Fa-Zuo Wang [1,3,]* and Si Zhang [1,]*

[1]CAS Key Laboratory of Tropical Marine Bio-Resources and Ecology, Southern Marine Science and Engi-neering Guangdong Laboratory (Guangzhou), Guangdong Key Laboratory of Marine Materia Medica, RNAM Center for Marine Microbiology, South China Sea Institute of Oceanology, Chinese Academy of Sciences, 164 West Xingang Road, Guangzhou 510301, China; cheyihao1995@163.com (Y.-H.C.); dingwen-ping19@mails.ucas.ac.cn (W.-P.D.); xzh@scsio.ac.cn (Z.-H.X.); yinhao@scsio.ac.cn (H.Y.); eira3558@163.com (J.-M.W.)

[2]University of Chinese Academy of Sciences, 19 Yuquan Road, Beijing 100049, China

[3]Equipment Public Service Center of South China Sea Institute of Oceanology, Chinese Academy of Sciences, 164 West Xingang Road, Guangzhou 510301, China

[*]Correspondence: wangfazuo@scsio.ac.cn (F.-Z.W.); zhsimd@scsio.ac.cn (S.Z.); Tel.: +86-020-3406-3746 (F.-Z.W.)

Abstract

Seven new phenol derivatives subversins A–E (**1**–**5**), subversic acid A (**6**) and *epi*-wortmannine G (**7**), one new natural product 4-hydroxy-7-methoxyphthalide (**8**), together with five known compounds (**9**–**13**) were isolated from the fungus *Aspergillus subversicolor* CYH-17 collected from the Haima cold seep. The structures and absolute configurations of these compounds were determined via NMR, MS, optical rotation, electronic circular dichroism (ECD) calculation, X-ray diffraction analysis and comparing with literatures. Compounds **2** and **5** were two pairs of enantiomers. All compounds were tested for their α-glucosidase and acetylcholinesterase (AChE) inhibitory activity, antioxidant activity and antibacterial activity, but no obvious activity was observed among these studied compounds.

Key words: Cold seep; fungi; phenol derivatives; activity

Contents

Figure S1. HR-ESI-MS spectra of compound **1**.

Figure S2. ^{1}H NMR spectra of compound **1**.

Figure S3. ^{13}C NMR and DEPT 135° spectra of compound **1**.

Figure S4. HSQC spectra of compound **1**.

Figure S5. HMBC spectra of compound **1**.

Figure S6. ^{1}H-^{1}H COSY spectra of compound **1**.

Figure S7. NOESY spectra of compound **1**.

Figure S8. UV spectra of compound **1**.

Figure S9. IR spectra of compound **1**.

Figure S10. HR-ESI-MS spectra of compound **2**.

Figure S11. ^{1}H NMR spectra of compound **2**.

Figure S12. ^{13}C NMR and DEPT 135° spectra of compound **2**.

Figure S13. HSQC spectra of compound **2**.

Figure S14. HMBC spectra of compound **2**.

Figure S15. ^{1}H-^{1}H COSY spectra of compound **2**.

Figure S16. NOESY spectra of compound **2**.

Figure S17. UV spectra of compound **2**.

Figure S18. IR spectra of compound **2**.

Figure S19. HR-ESI-MS spectra of compound **3**.

Figure S20. ^{1}H NMR spectra of compound **3**.

Figure S21. ^{13}C NMR and DEPT 135° spectra of compound **3**.

Figure S22. HSQC spectra of compound **3**.

Figure S23. HMBC spectra of compound **3**.

Figure S24. ^{1}H-^{1}H COSY spectra of compound **3**.

Figure S25. NOESY spectra of compound **3**.

Figure S26. UV spectra of compound **3**.

Figure S27. IR spectra of compound **3**.

Figure S28. HR-ESI-MS spectra of compound **4**.

Figure S29. ^{1}H NMR spectra of compound **4**.

Figure S30. ^{13}C NMR and DEPT 135° spectra of compound **4**.

Figure S31. HSQC spectra of compound **4**.

Figure S32. HMBC spectra of compound **4**.

Figure S33. ^{1}H-^{1}H COSY spectra of compound **4**.

Figure S34. NOESY spectra of compound **4**.

Figure S35. UV spectra of compound **4**.

Figure S36. IR spectra of compound **4**.

Figure S37. HR-ESI-MS spectra of compound **5**.

Figure S38. ^{1}H NMR spectra of compound **5**.

Figure S39. ^{13}C NMR and DEPT 135° spectra of compound **5**.

Figure S40. HSQC spectra of compound **5**.

Figure S41. HMBC spectra of compound **5**.

Figure S42. ^{1}H-^{1}H COSY spectra of compound **5**.

Figure S43. NOESY spectra of compound **5**.

Figure S44. UV spectra of compound **5**.

Figure S45. IR spectra of compound **5**.

Figure S46. HR-ESI-MS spectra of compound **6**.

Figure S47. ^{1}H NMR spectra of compound **6**.

Figure S48. ^{13}C NMR and DEPT 135° spectra of compound **6**.

Figure S49. HSQC spectra of compound **6**.

Figure S50. HMBC spectra of compound **6**.

Figure S51. ^{1}H-^{1}H COSY spectra of compound **6**.

Figure S52. NOESY spectra of compound **6**.

Figure S53. UV spectra of compound **6**.

Figure S54. IR spectra of compound **6**.

Figure S55. HR-ESI-MS spectra of compound **7**.

Figure S56. ^{1}H NMR spectra of compound **7**.

Figure S57. ^{13}C NMR and DEPT 135° spectra of compound **7**.

Figure S58. HSQC spectra of compound **7**.

Figure S59. HMBC spectra of compound **7**.

Figure S60. ^{1}H-^{1}H COSY spectra of compound **7**.

Figure S61. NOESY spectra of compound **7**.

Figure S62. UV spectra of compound **7**.

Figure S63. IR spectra of compound **7**.

Figure S64. HR-ESI-MS spectra of compound **8**.

Figure S65. ^{1}H NMR spectra of compound **8**.

Figure S66. ^{13}C NMR and DEPT 135° spectra of compound **8**.

Figure S67. HSQC spectra of compound **8**.

Figure S68. HMBC spectra of compound **8**.

Figure S69. ^{1}H-^{1}H COSY spectra of compound **8**.

Figure S70. UV spectra of compound **8**.

Figure S71. IR spectra of compound **8**.

Figure S72. The chiral HPLC separation profile of compound **2**.

ECD calculation of **2**.

Table S1. Energies at B3LYP/6–311g (d, p) level of 3*R*-**2** and 3*S*-**2**.

Figure S73. Comparison between the experimental ECD and the calculated ECD spectra for compound (+)-**2** and (−)-**2**.

Figure S74. The chiral HPLC separation profile of compound **5**.

Figure S75. The experiment ECD of compounds (+)-**5 and** (−)-**5**.

Computational methods of **3**.

Figure S76. Two computational configurations of 3*R**, 8*R** (**3a**) and 3*R**, 8*S** (**3b**).

Computational data of **3a**.

Figure S77. Optimized geometries of 11 dominant conformers of **3a** at the B3LYP-GD3bJ/6-31G(d) level of theory in methanol with the IEFPCM solvent.

Table S2. Conformational analysis of the optimized **3a** at the B3LYP/6-31G(d) of theory in methanol with the IEFPCM solvent.

Table S3. Cartesian coordinates of **3a-1**.

Table S4. Cartesian coordinates of **3a-2**.

Table S5. Cartesian coordinates of **3a-3**.

Table S6. Cartesian coordinates of **3a-4**.

Table S7. Cartesian coordinates of **3a-5**.

Table S8. Cartesian coordinates of **3a-6**.

Table S9. Cartesian coordinates of **3a-7**.

Table S10. Cartesian coordinates of **3a-9**.

Table S11. Cartesian coordinates of **3a-10**.

Table S12. Cartesian coordinates of **3a-11**.

Table S13. Cartesian coordinates of **3a-14**.

Computational data of **3b**.

Figure S78. Optimized geometries of 14 dominant conformers of **3b** at the B3LYP-GD3bJ/6-31G(d) level of theory in methanol with the IEFPCM solvent.

Table S14. Conformational analysis of the optimized **3b** at the B3LYP/6-31G(d) of theory in methanol with the IEFPCM solvent.

Table S15. Cartesian coordinates of **3b-1**.

Table S16. Cartesian coordinates of **3b-2**.

Table S17. Cartesian coordinates of **3b-3**.

Table S18. Cartesian coordinates of **3b-4**.

Table S19. Cartesian coordinates of **3b-5**.

Table S20. Cartesian coordinates of **3b-6**.

Table S21. Cartesian coordinates of **3b-7**.

Table S22. Cartesian coordinates of **3b-8**.

Table S23. Cartesian coordinates of **3b-9**.

Table S24. Cartesian coordinates of **3b-10**.

Table S25. Cartesian coordinates of **3b-11**.

Table S26. Cartesian coordinates of **3b-12**.

Table S27. Cartesian coordinates of **3b-13**.

Table S28. Cartesian coordinates of **3b-14**.

Figure S79. Linear regression analysis between the experimental and calculated NMR

chemical shifts of **3a**.
Figure S80. Linear regression analysis between the experimental and calculated NMR chemical shifts of **3b**.
Table S29. The experimental and calculated ^{13}C NMR chemical shifts of **3**, **3a** and **3b** and the MAE and CMAE analysis of **3a** and **3b**.
Table S30. The experimental and calculated ^{1}H NMR chemical shifts of **3**, **3a** and **3b** and the MAE and CMAE analysis of **3a** and **3b**.
Table S31. The DP4+ probabilities of **3a**-**3b**.
Figure S81. Comparison between the experimental ECD and the calculated ECD spectra for compound **3**.
The ITS sequence of A. subversicolor CYH-17
Table S32. The antibacterial activity of the compounds.
Table S33. The α-glucosidase inhibitory activity and antioxidant activity of the compounds.

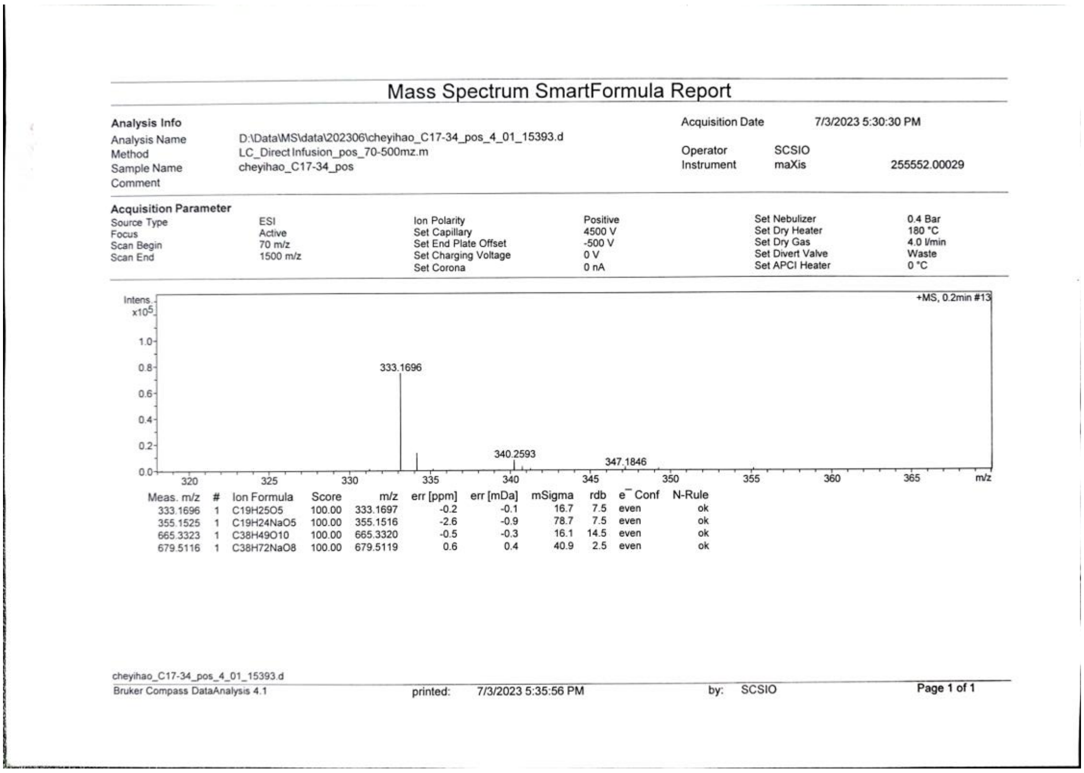

Mass Spectrum SmartFormula Report

Analysis Info

Analysis Name: D:\Data\MS\data\202306\cheyihao_C17-34_pos_4_01_15393.d
Method: LC_Direct Infusion_pos_70-500mz.m
Sample Name: cheyihao_C17-34_pos
Comment:

Acquisition Date: 7/3/2023 5:30:30 PM
Operator: SCSIO
Instrument: maXis 255552.00029

Acquisition Parameter

Source Type	ESI	Ion Polarity	Positive	Set Nebulizer	0.4 Bar
Focus	Active	Set Capillary	4500 V	Set Dry Heater	180 °C
Scan Begin	70 m/z	Set End Plate Offset	-500 V	Set Dry Gas	4.0 l/min
Scan End	1500 m/z	Set Charging Voltage	0 V	Set Divert Valve	Waste
		Set Corona	0 nA	Set APCI Heater	0 °C

Meas. m/z	#	Ion Formula	Score	m/z	err [ppm]	err [mDa]	mSigma	rdb	e⁻ Conf	N-Rule
333.1696	1	C19H25O5	100.00	333.1697	-0.2	-0.1	16.7	7.5	even	ok
355.1525	1	C19H24NaO5	100.00	355.1516	-2.6	-0.9	78.7	7.5	even	ok
665.3323	1	C38H49O10	100.00	665.3320	-0.5	-0.3	16.1	14.5	even	ok
679.5116	1	C38H72NaO8	100.00	679.5119	0.6	0.4	40.9	2.5	even	ok

cheyihao_C17-34_pos_4_01_15393.d
Bruker Compass DataAnalysis 4.1 printed: 7/3/2023 5:35:56 PM by: SCSIO Page 1 of 1

Figure S1. HR-ESI-MS spectra of compound **1**.

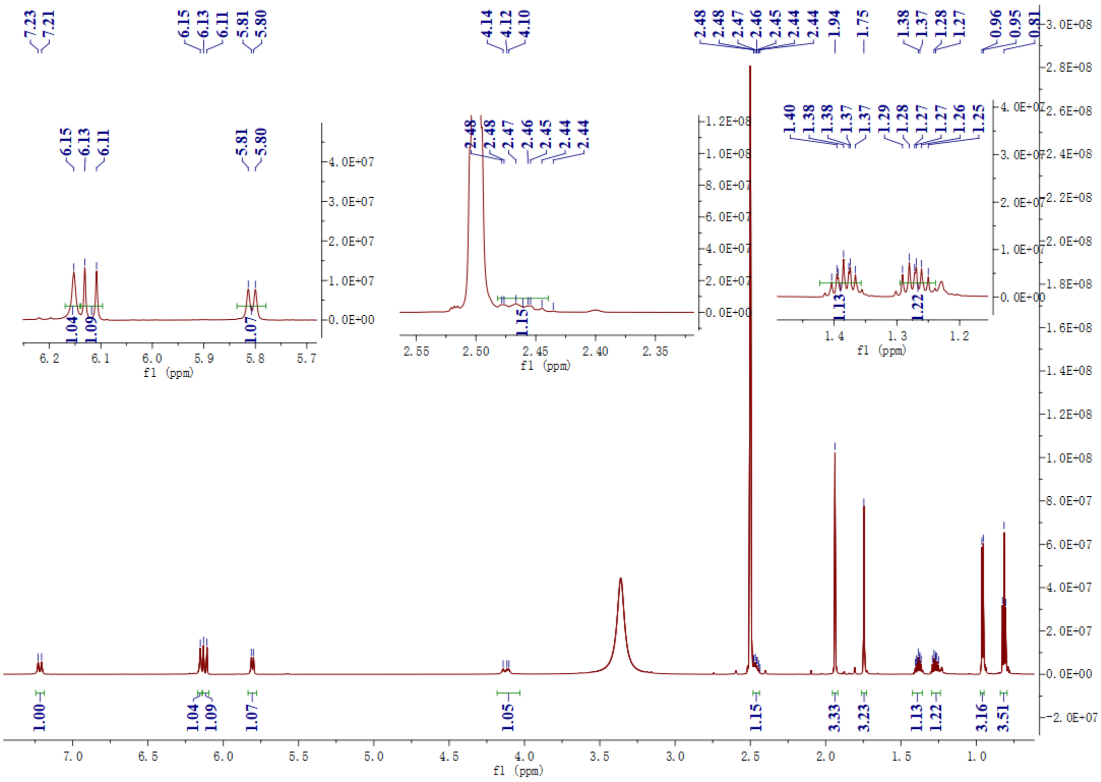

Figure S2. ^{1}H NMR spectra of compound **1**.

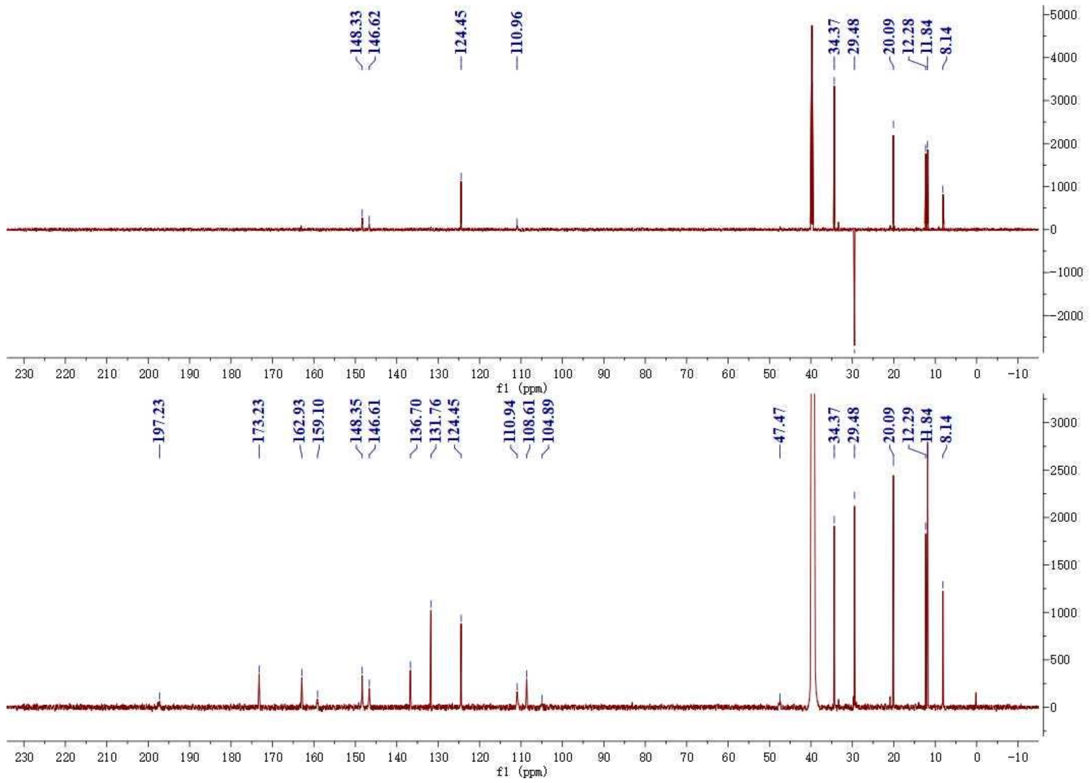

Figure S3. ^{13}C NMR and DEPT 135° spectra of compound **1**.

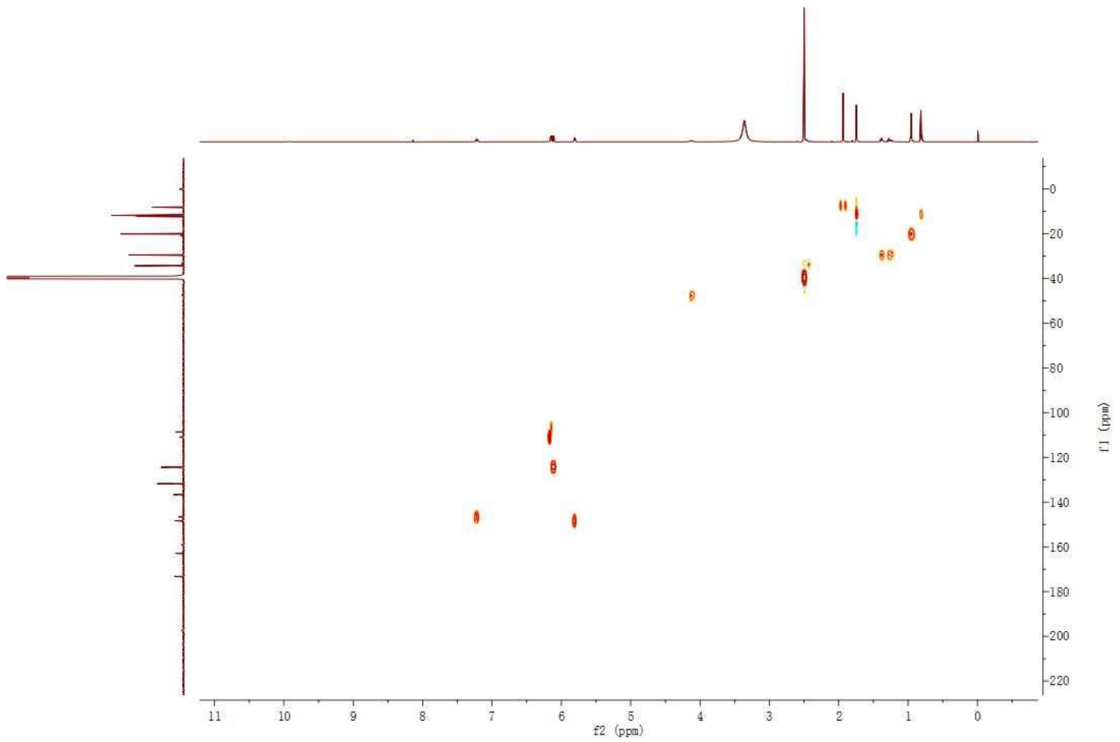

Figure S4. HSQC spectra of compound **1**.

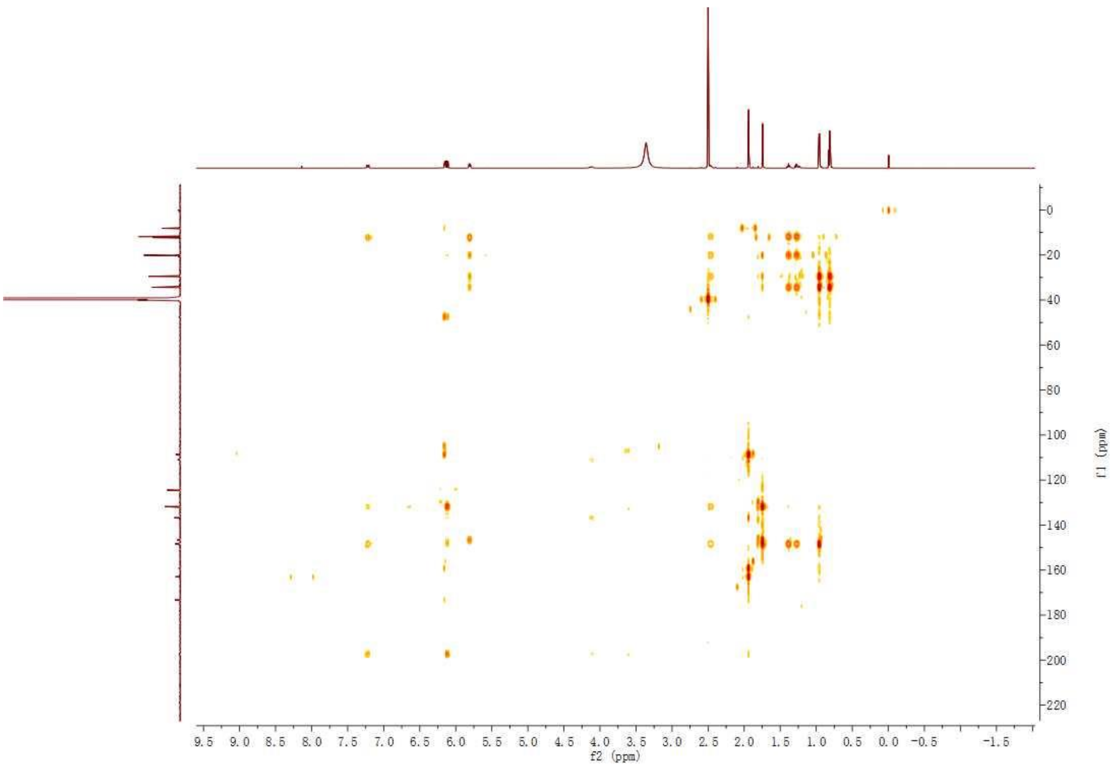

Figure S5. HMBC spectra of compound **1**.

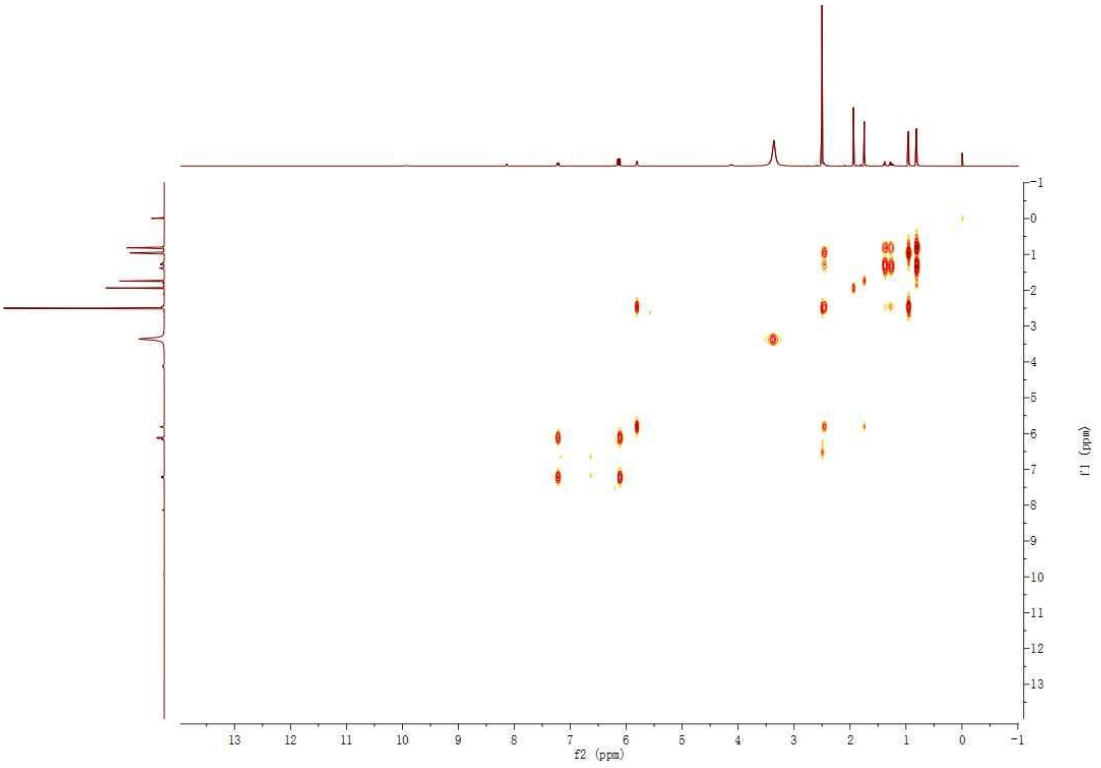

Figure S6. ^{1}H-^{1}H COSY spectra of compound **1**.

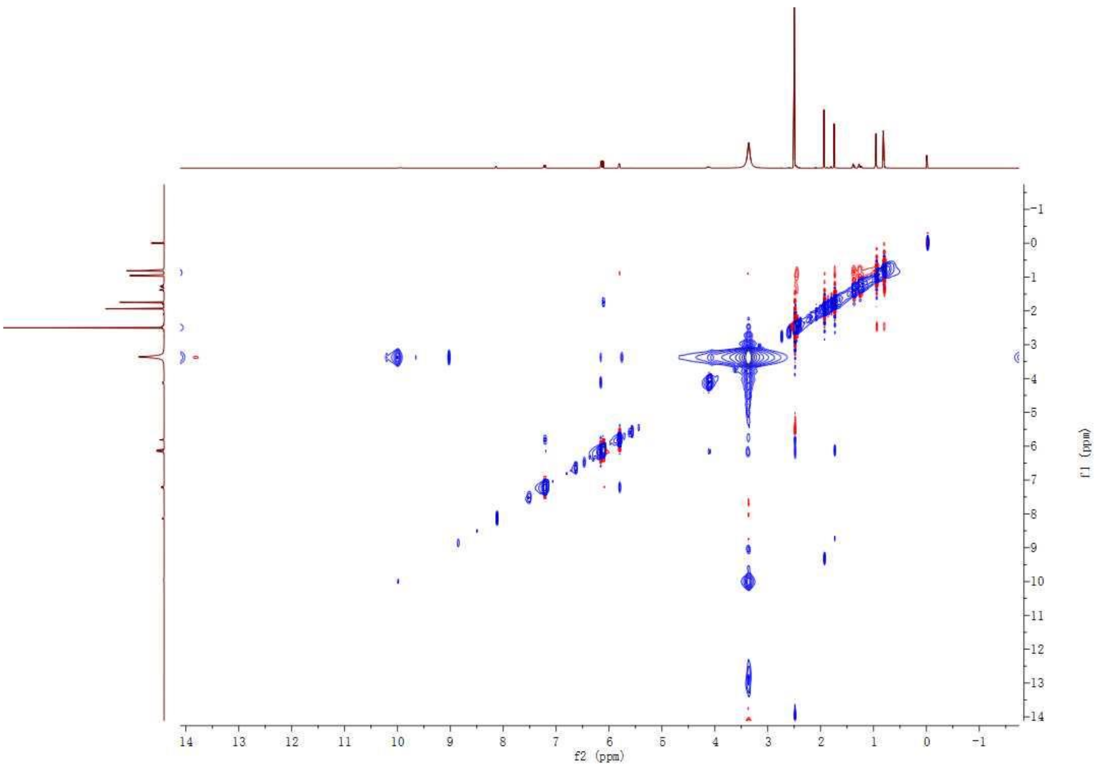

Figure S7. NOESY spectra of compound **1**.

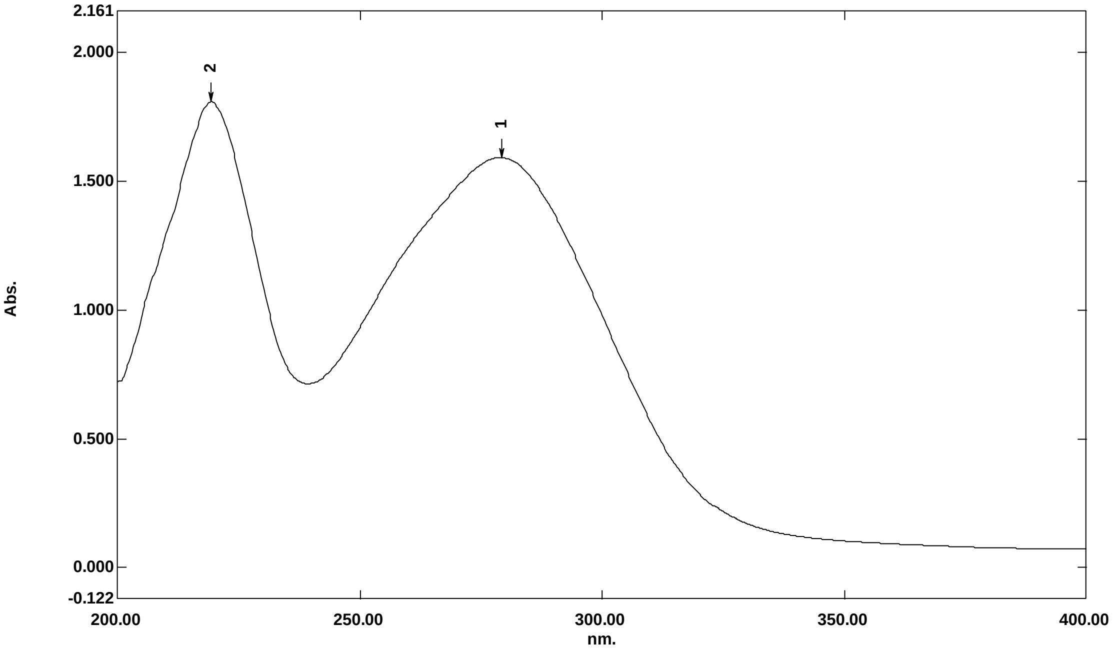

No.	波长(nm)	Abs.
1	279.20	1.592
2	219.20	1.810

Figure S8. UV spectra of compound **1**.

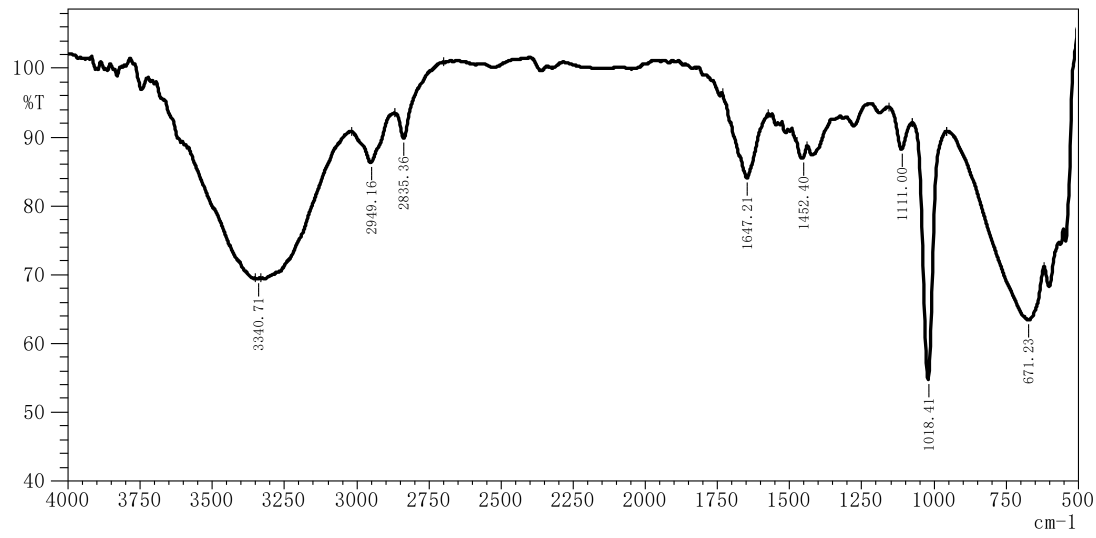

Figure S9. IR spectra of compound **1**.

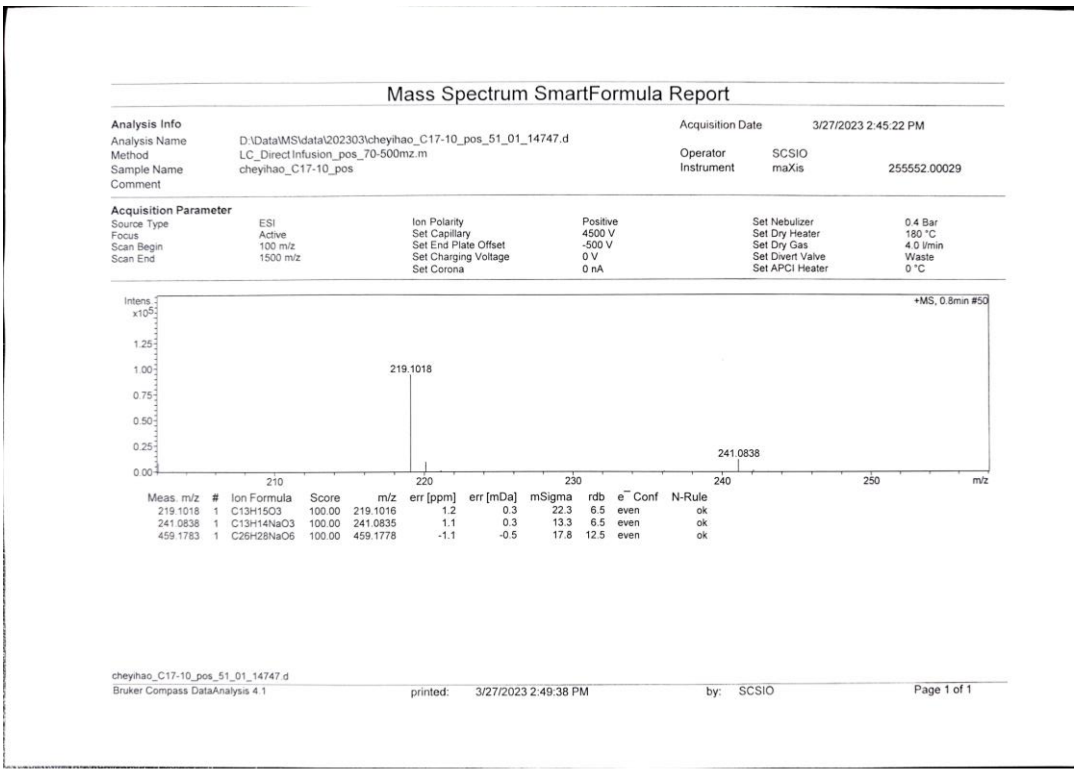

Figure S10. HR-ESI-MS spectra of compound **2**.

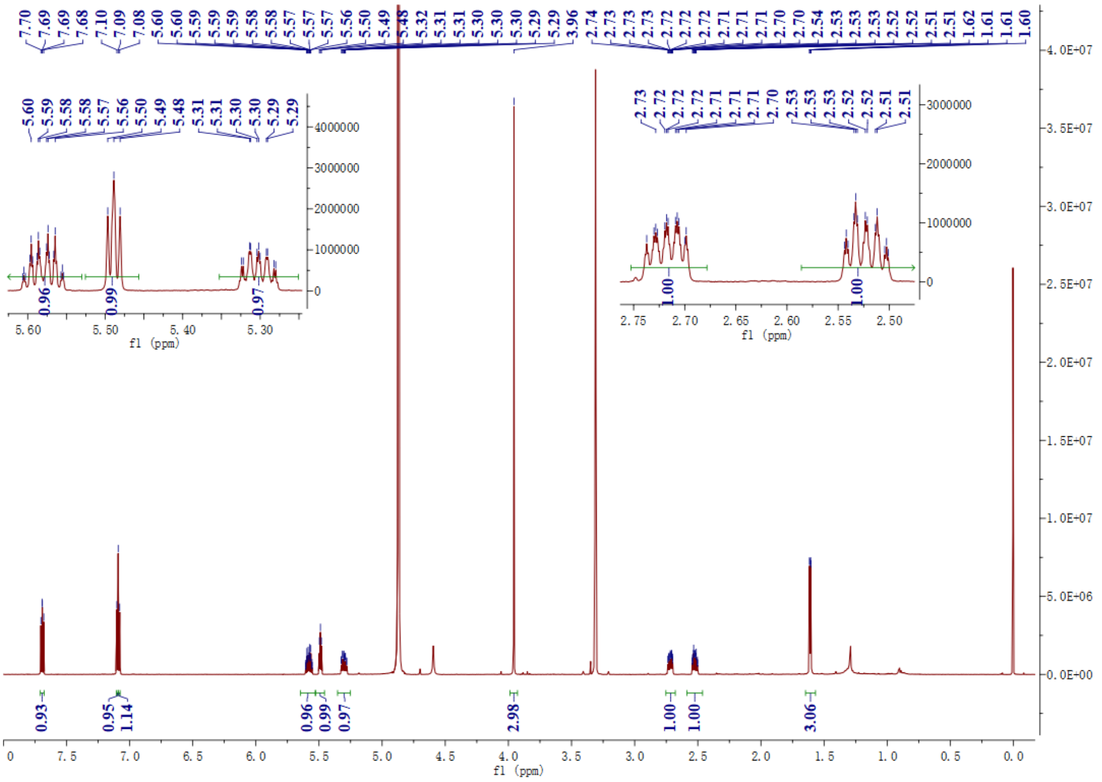

Figure S11. ^{1}H NMR spectra of compound **2**.

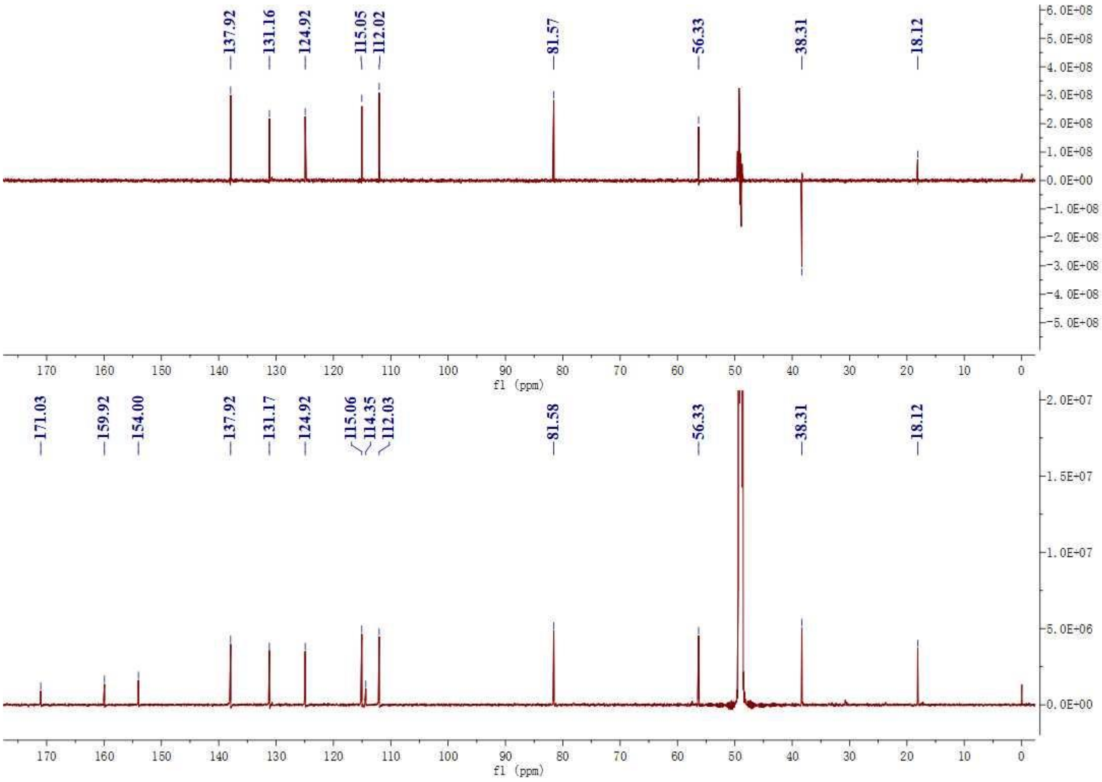

Figure S12. ^{13}C NMR and DEPT 135° spectra of compound **2**.

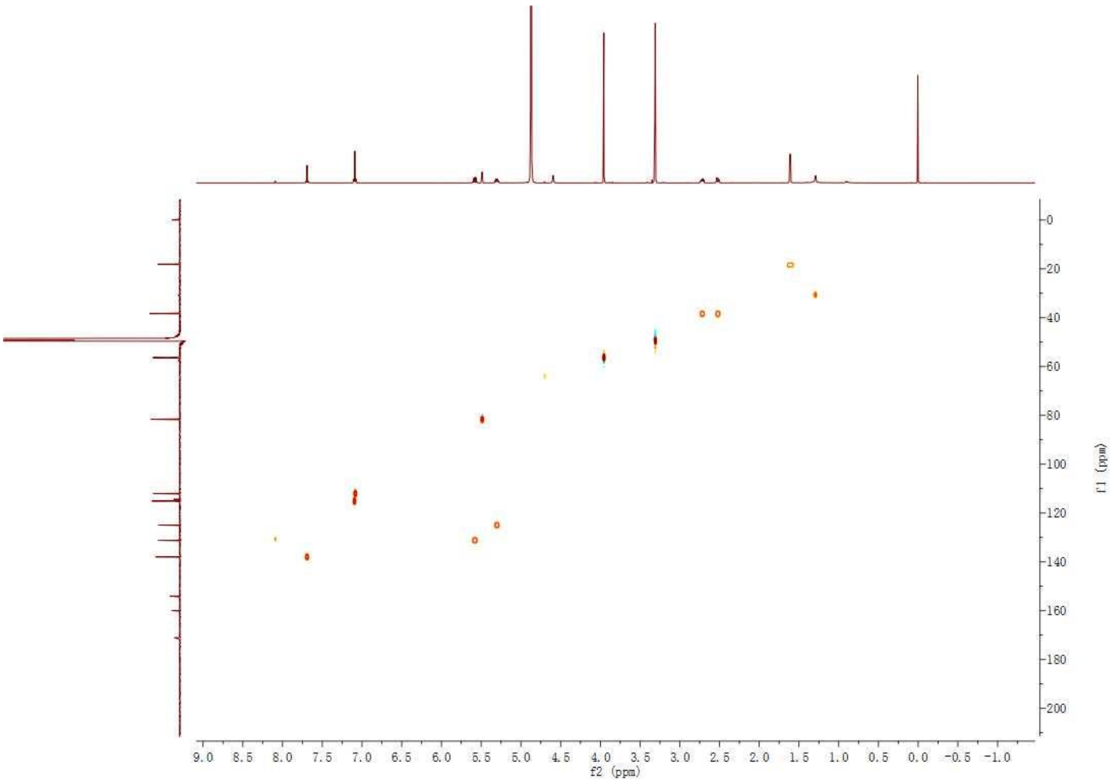

Figure S13. HSQC spectra of compound **2**.

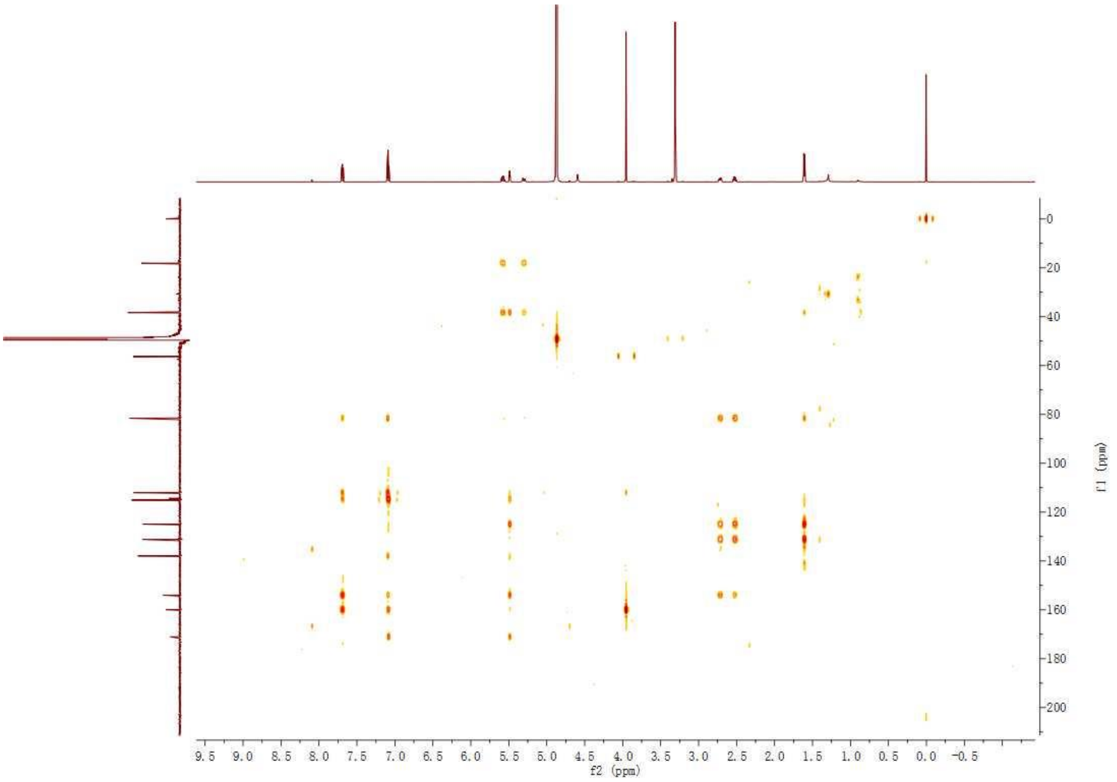

Figure S14. HMBC spectra of compound **2**.

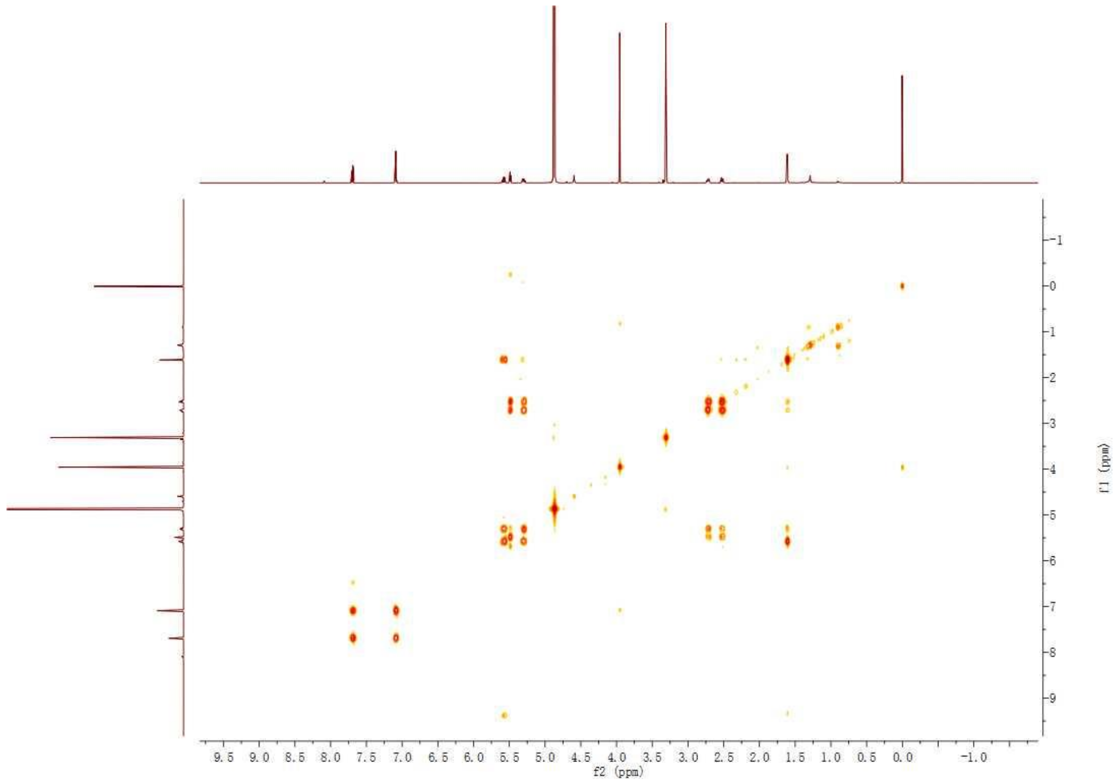

Figure S15. ^{1}H-^{1}H COSY spectra of compound **2**.

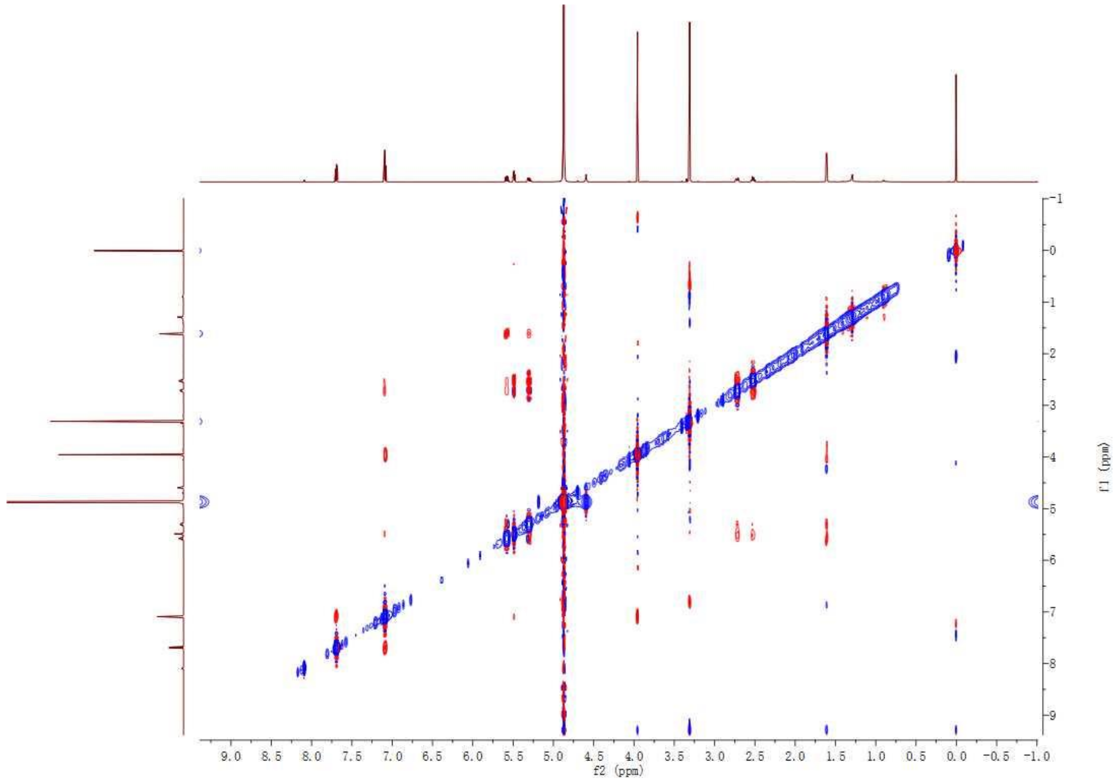

Figure S16. NOESY spectra of compound **2**.

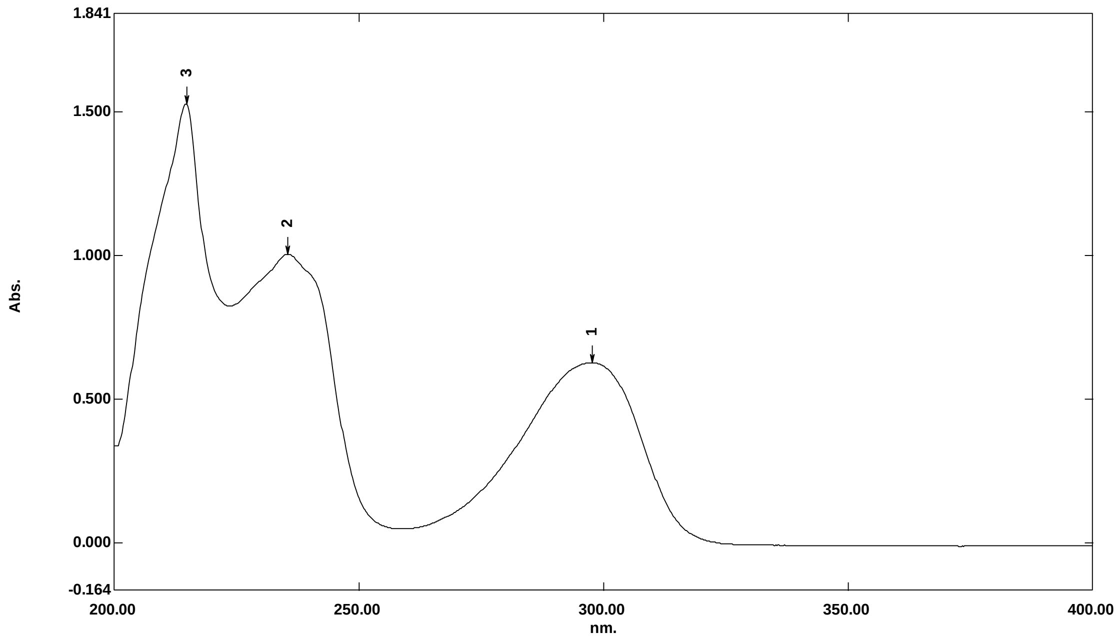

No.	波长(nm)	Abs.
1	297.60	0.627
2	235.40	1.005
3	214.80	1.527

Figure S17. UV spectra of compound **2**.

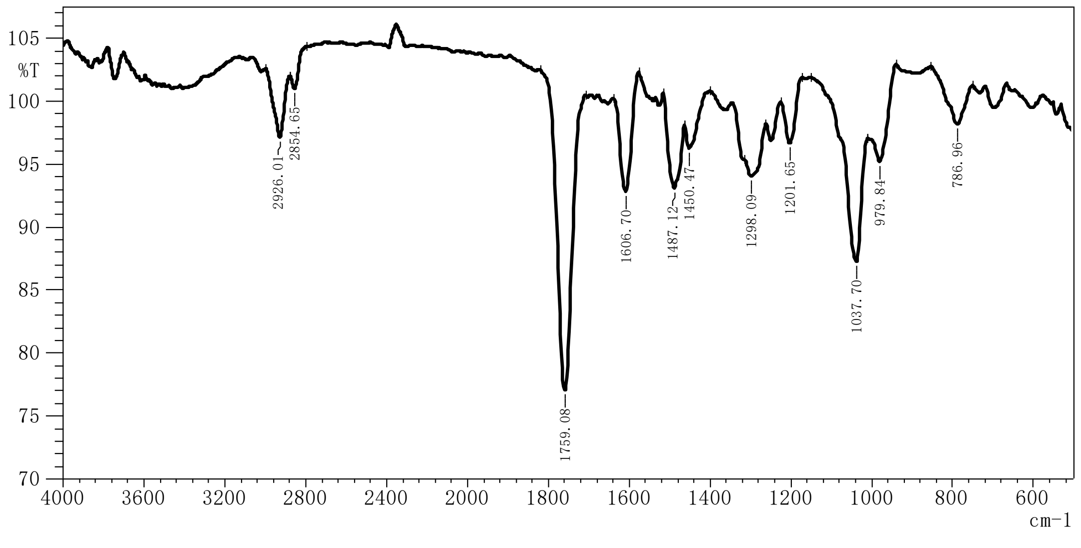

Figure S18. IR spectra of compound **2**.

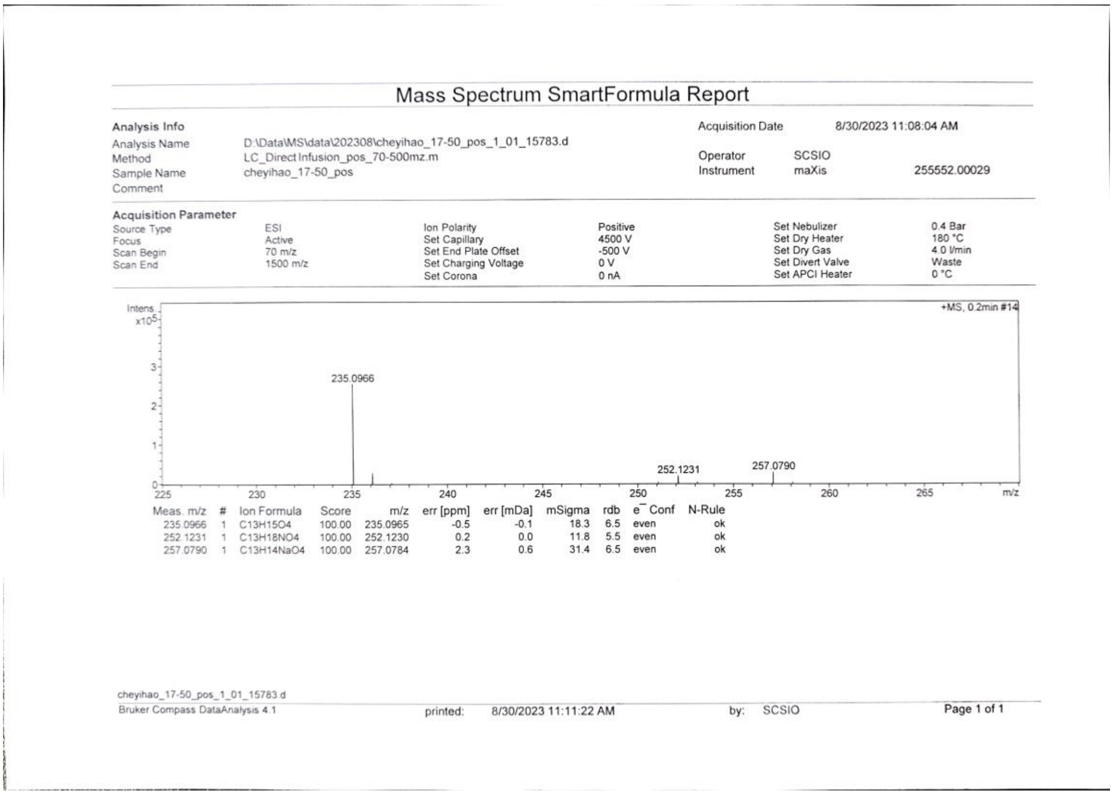

Mass Spectrum SmartFormula Report

Analysis Info		Acquisition Date	8/30/2023 11:08:04 AM
Analysis Name	D:\Data\MS\data\202308\cheyihao_17-50_pos_1_01_15783.d		
Method	LC_Direct Infusion_pos_70-500mz.m	Operator	SCSIO
Sample Name	cheyihao_17-50_pos	Instrument	maXis 255552.00029
Comment			

Acquisition Parameter

Source Type	ESI	Ion Polarity	Positive	Set Nebulizer	0.4 Bar
Focus	Active	Set Capillary	4500 V	Set Dry Heater	180 °C
Scan Begin	70 m/z	Set End Plate Offset	-500 V	Set Dry Gas	4.0 l/min
Scan End	1500 m/z	Set Charging Voltage	0 V	Set Divert Valve	Waste
		Set Corona	0 nA	Set APCI Heater	0 °C

Meas. m/z	#	Ion Formula	Score	m/z	err [ppm]	err [mDa]	mSigma	rdb	e⁻ Conf	N-Rule
235.0966	1	C13H15O4	100.00	235.0965	-0.5	-0.1	18.3	6.5	even	ok
252.1231	1	C13H18NO4	100.00	252.1230	0.2	0.0	11.8	5.5	even	ok
257.0790	1	C13H14NaO4	100.00	257.0784	2.3	0.6	31.4	6.5	even	ok

cheyihao_17-50_pos_1_01_15783.d
Bruker Compass DataAnalysis 4.1 printed: 8/30/2023 11:11:22 AM by: SCSIO Page 1 of 1

Figure S19. HR-ESI-MS spectra of compound **3**.

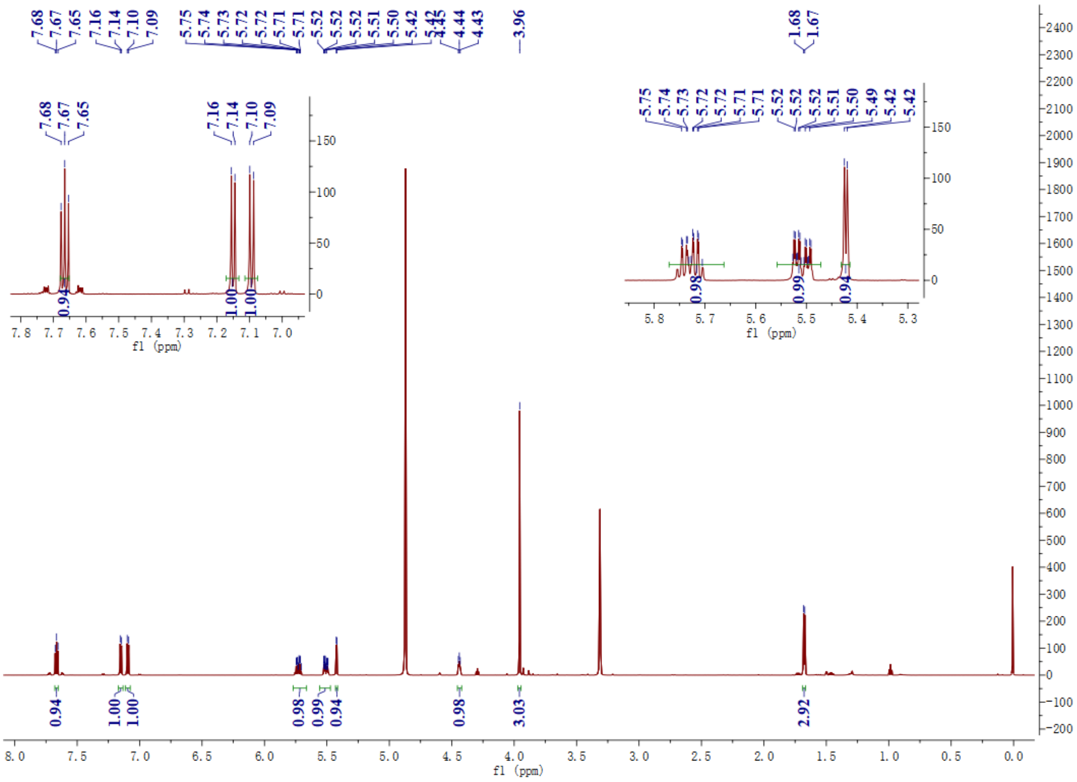

Figure S20. ^{1}H NMR spectra of compound **3**.

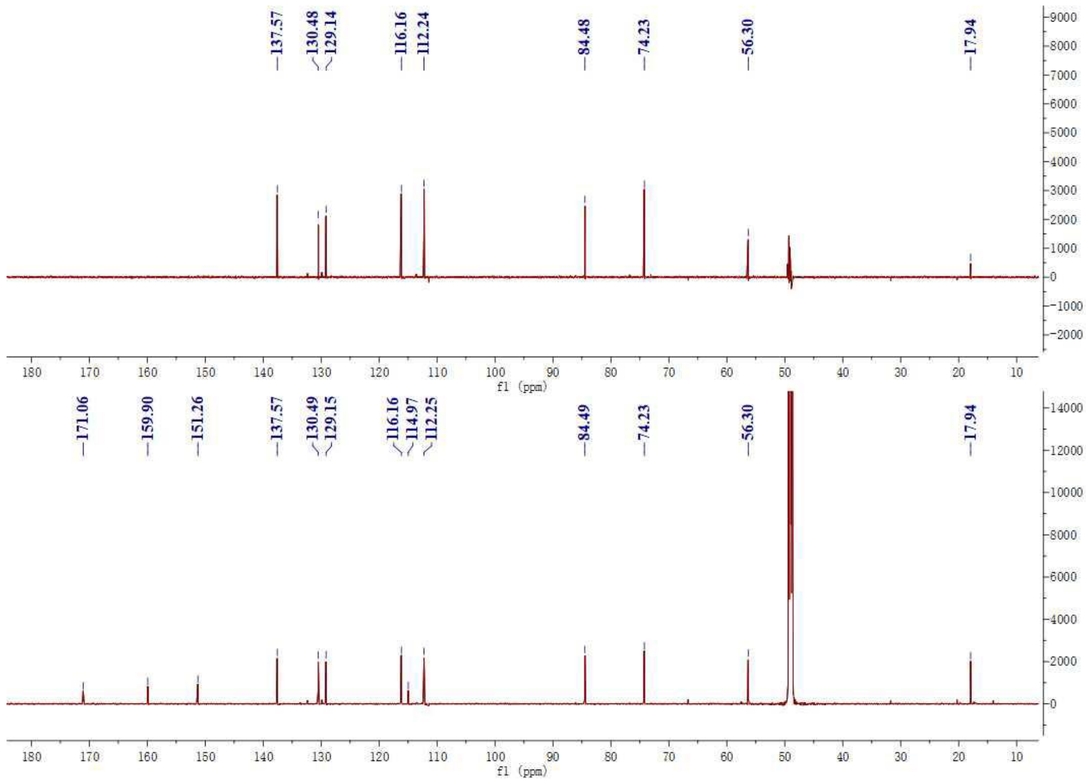

Figure S21. ^{13}C NMR and DEPT 135° spectra of compound **3**.

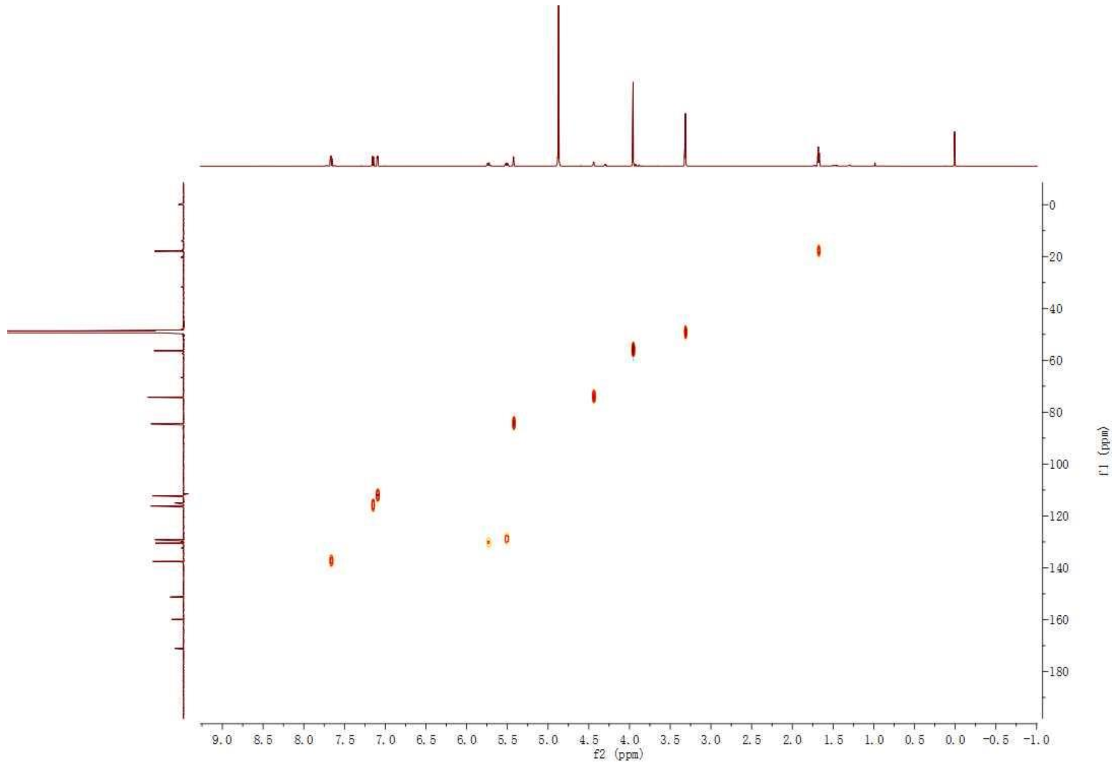

Figure S22. HSQC spectra of compound **3**.

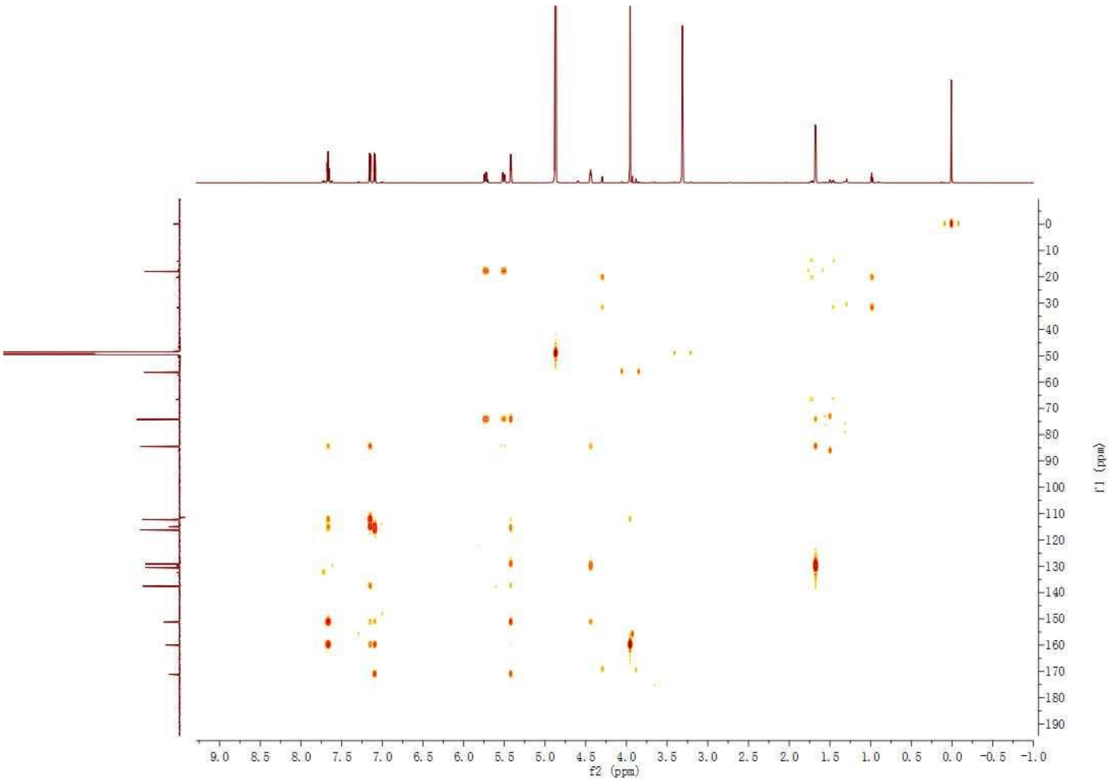

Figure S23. HMBC spectra of compound **3**.

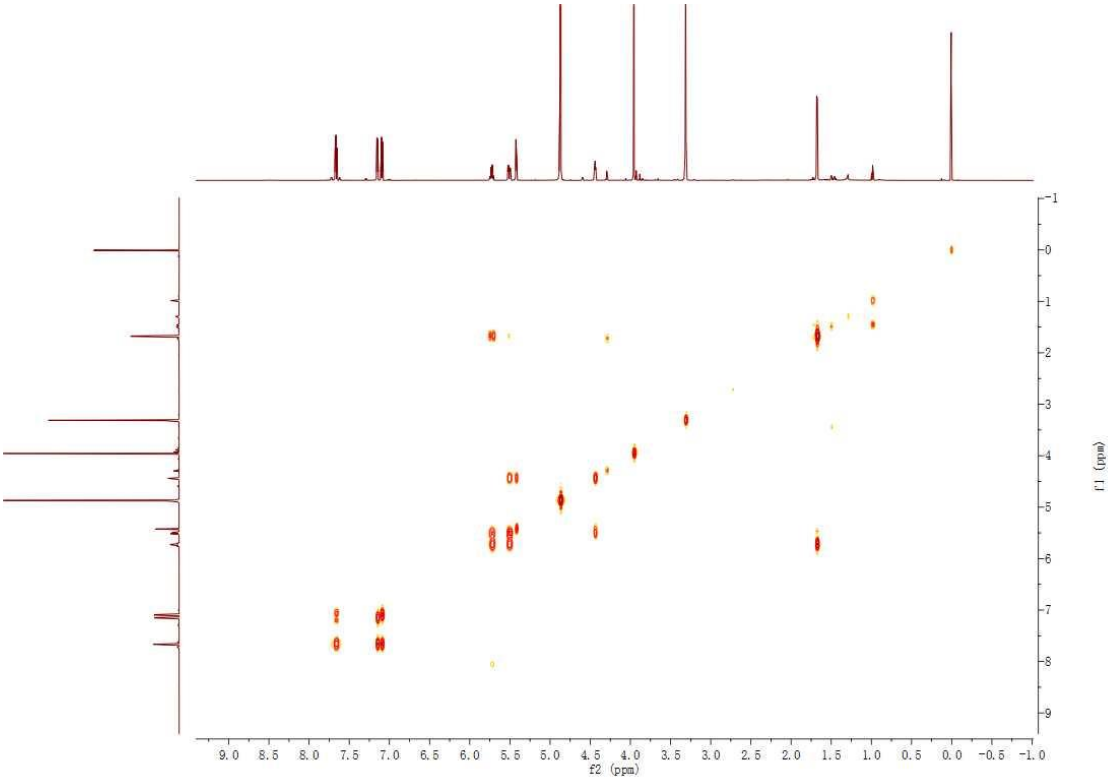

Figure S24. ^{1}H-^{1}H COSY spectra of compound **3**.

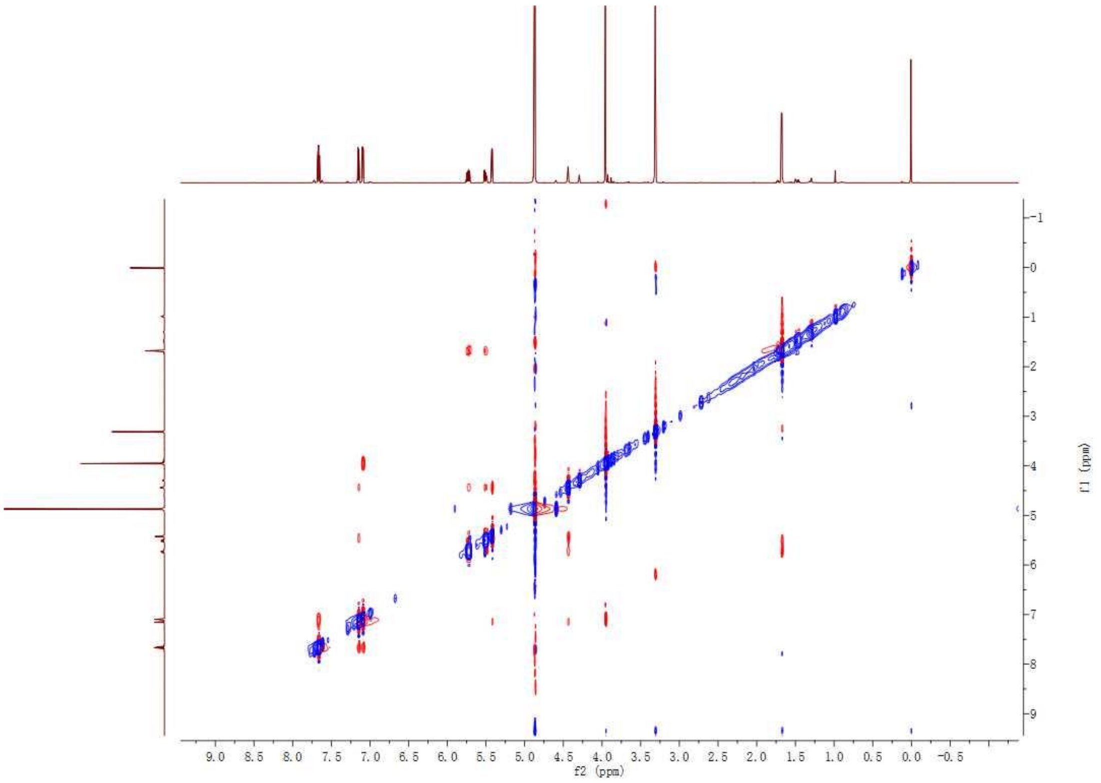

Figure S25. NOESY spectra of compound **3**.

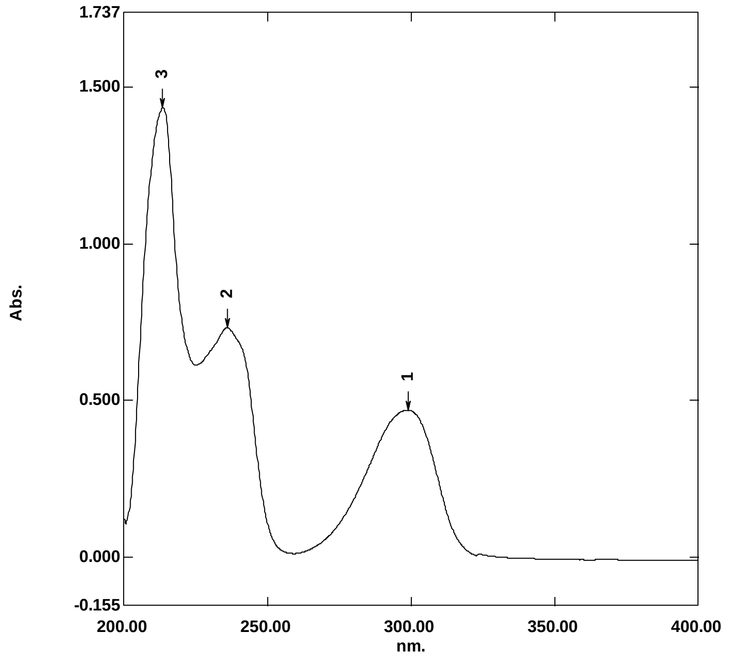

No.	波长(nm)	Abs.
1	298.80	0.468
2	235.80	0.731
3	213.20	1.434

Figure S26. UV spectra of compound **3**.

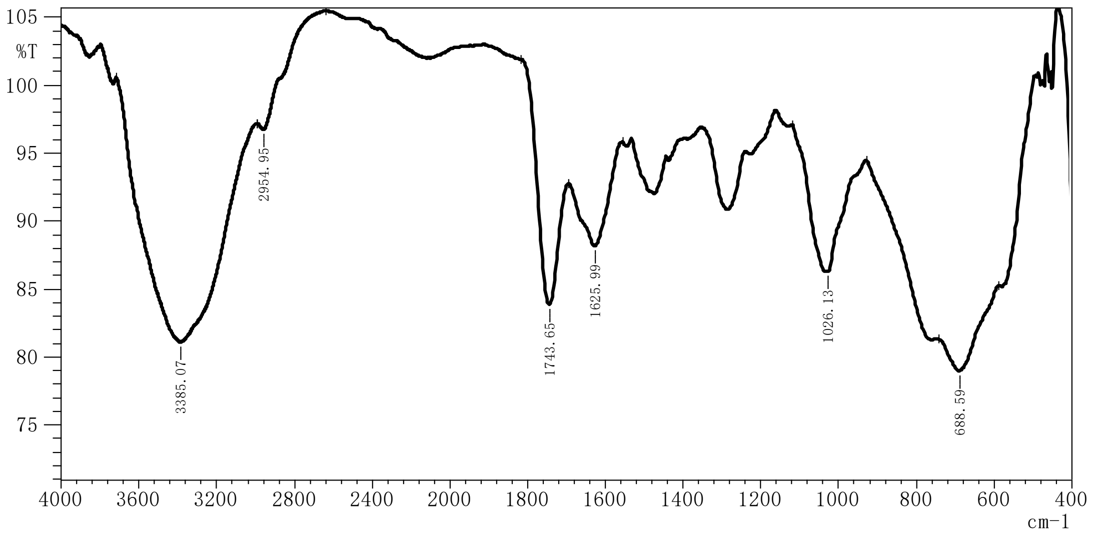

Figure S27. IR spectra of compound **3**.

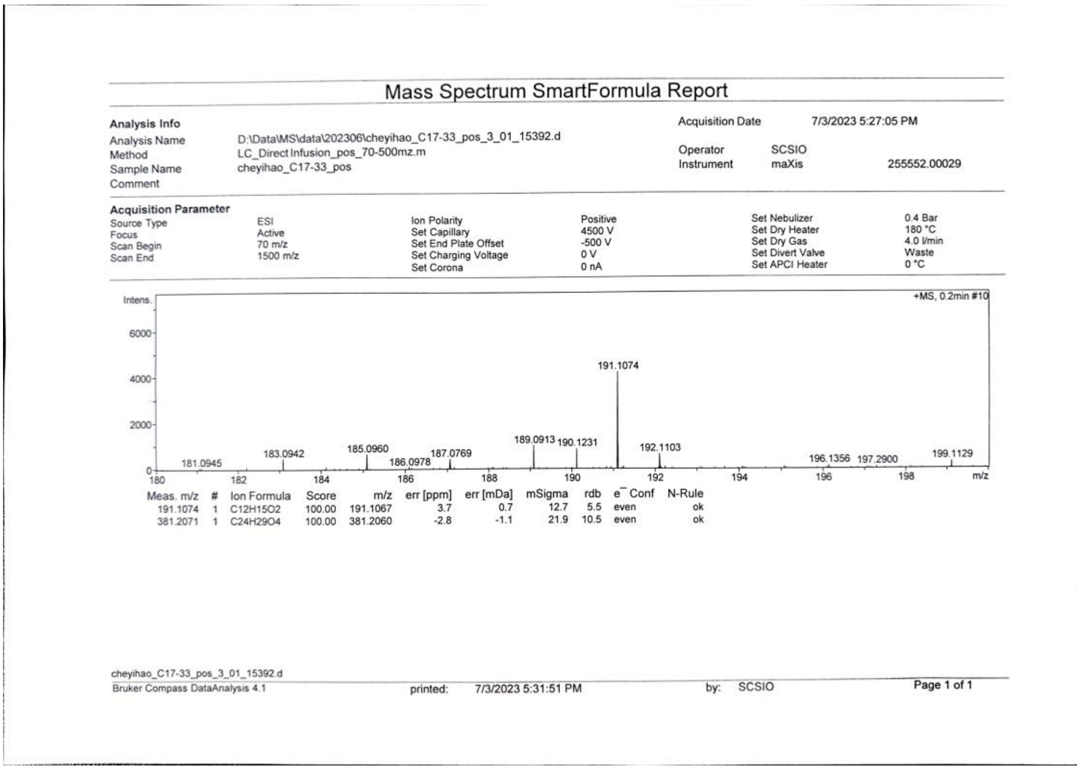

Mass Spectrum SmartFormula Report

**Analysis Info**		Acquisition Date	7/3/2023 5:27:05 PM
Analysis Name	D:\Data\MS\data\202306\cheyihao_C17-33_pos_3_01_15392.d		
Method	LC_Direct Infusion_pos_70-500mz.m	Operator	SCSIO
Sample Name	cheyihao_C17-33_pos	Instrument	maXis 255552.00029
Comment			

**Acquisition Parameter**					
Source Type	ESI	Ion Polarity	Positive	Set Nebulizer	0.4 Bar
Focus	Active	Set Capillary	4500 V	Set Dry Heater	180 °C
Scan Begin	70 m/z	Set End Plate Offset	-500 V	Set Dry Gas	4.0 l/min
Scan End	1500 m/z	Set Charging Voltage	0 V	Set Divert Valve	Waste
		Set Corona	0 nA	Set APCI Heater	0 °C

Meas. m/z	#	Ion Formula	Score	m/z	err [ppm]	err [mDa]	mSigma	rdb	e⁻ Conf	N-Rule
191.1074	1	C12H15O2	100.00	191.1067	3.7	0.7	12.7	5.5	even	ok
381.2071	1	C24H29O4	100.00	381.2060	-2.8	-1.1	21.9	10.5	even	ok

cheyihao_C17-33_pos_3_01_15392.d
Bruker Compass DataAnalysis 4.1 printed: 7/3/2023 5:31:51 PM by: SCSIO Page 1 of 1

Figure S28. HR-ESI-MS spectra of compound **4**.

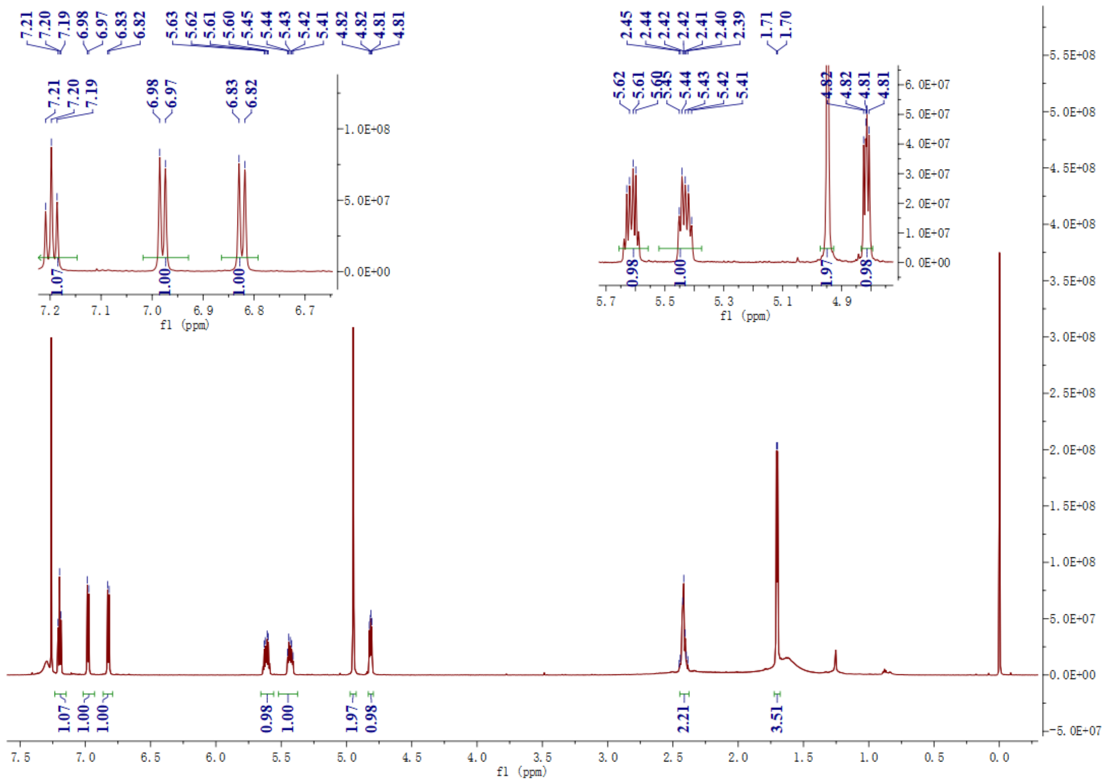

Figure S29. ^{1}H NMR spectra of compound **4**.

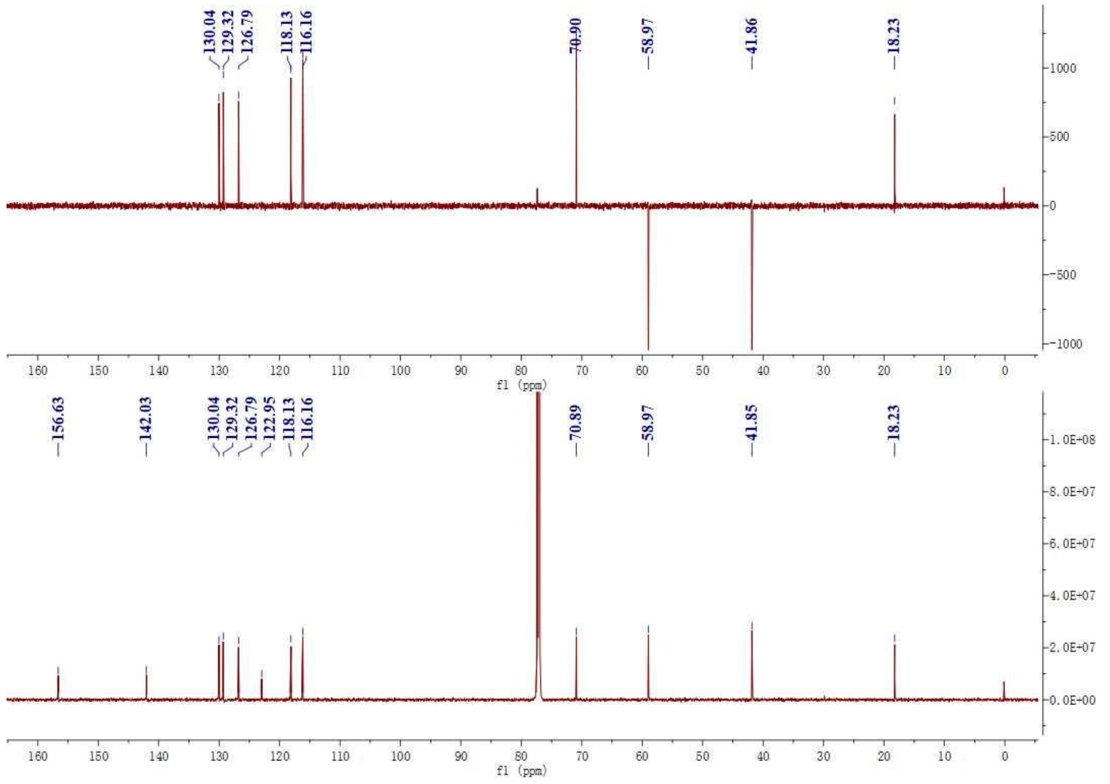

Figure S30. ^{13}C NMR and DEPT 135° spectra of compound **4**.

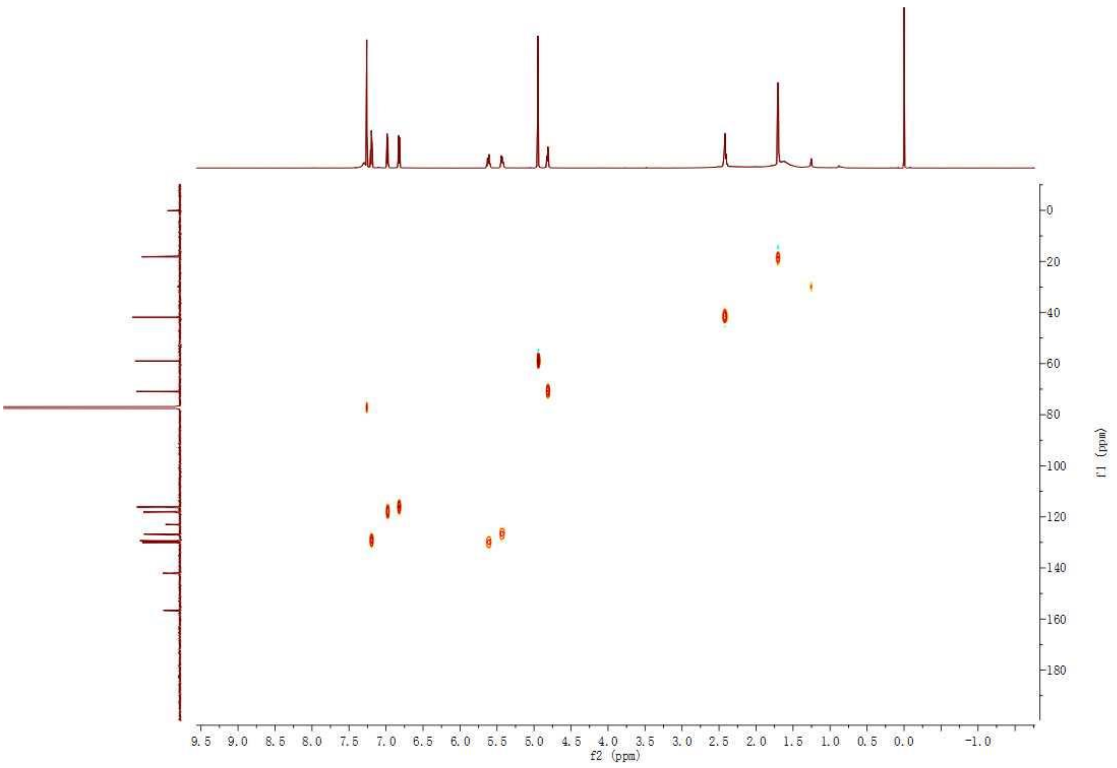

Figure S31. HSQC spectra of compound **4**.

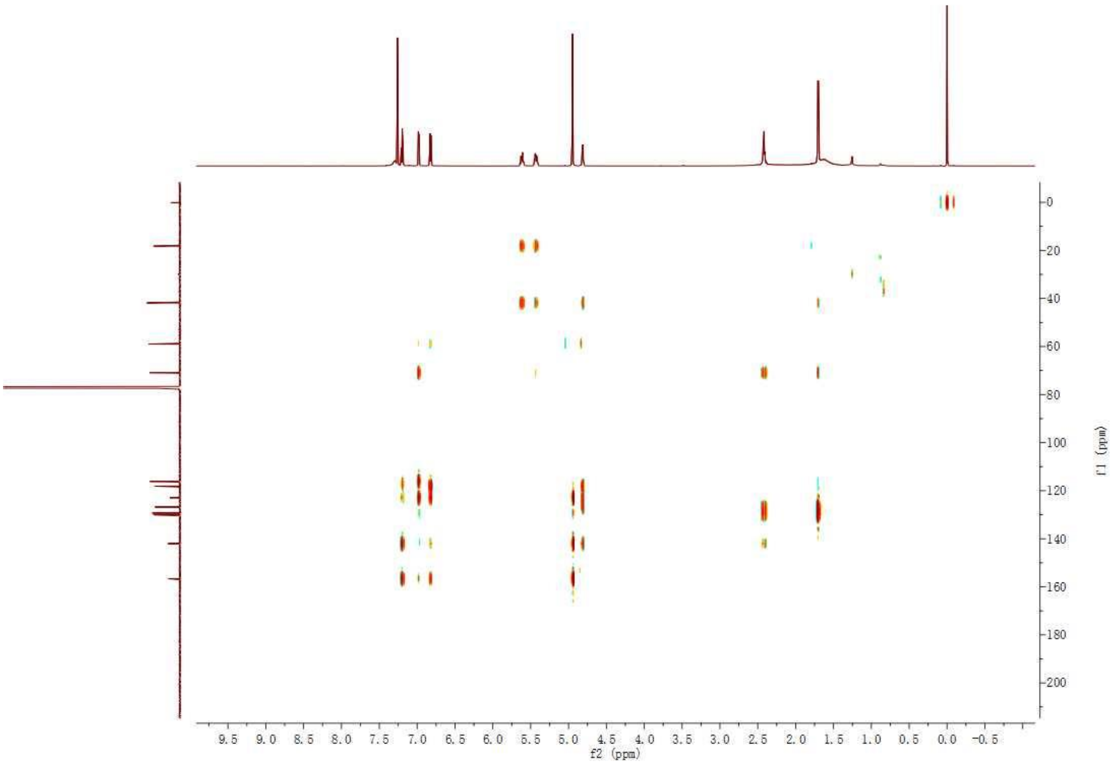

Figure S32. HMBC spectra of compound **4**.

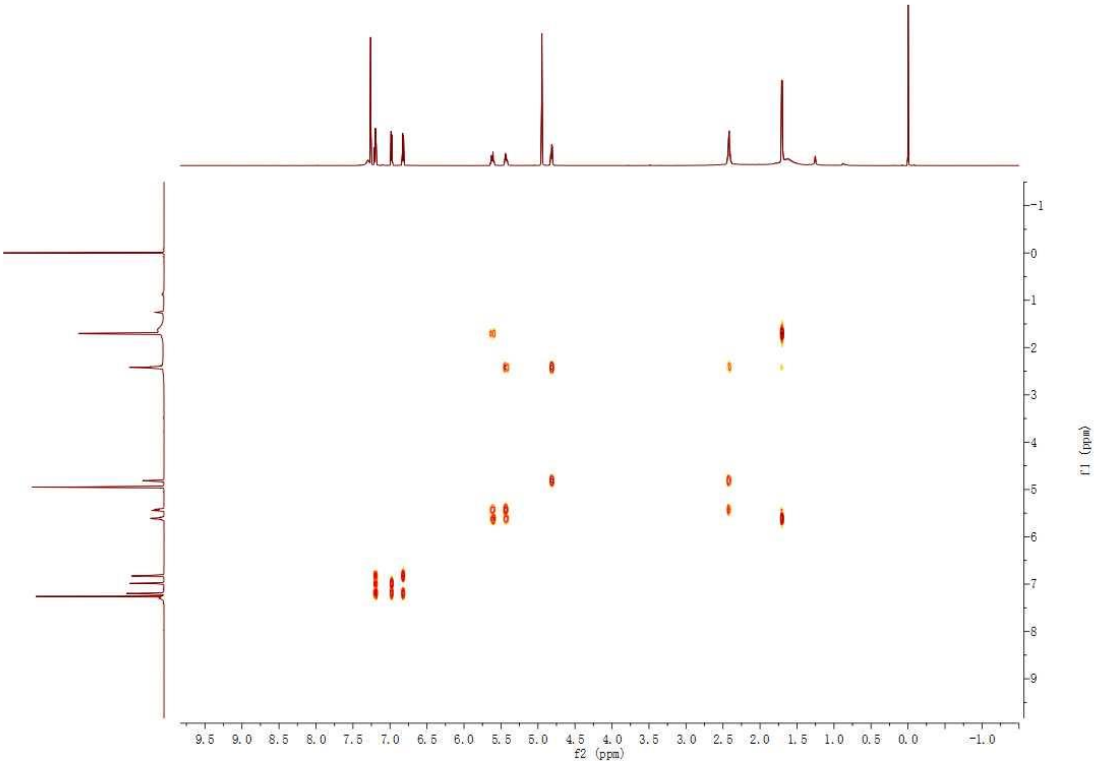

Figure S33. ^{1}H-^{1}H COSY spectra of compound **4**.

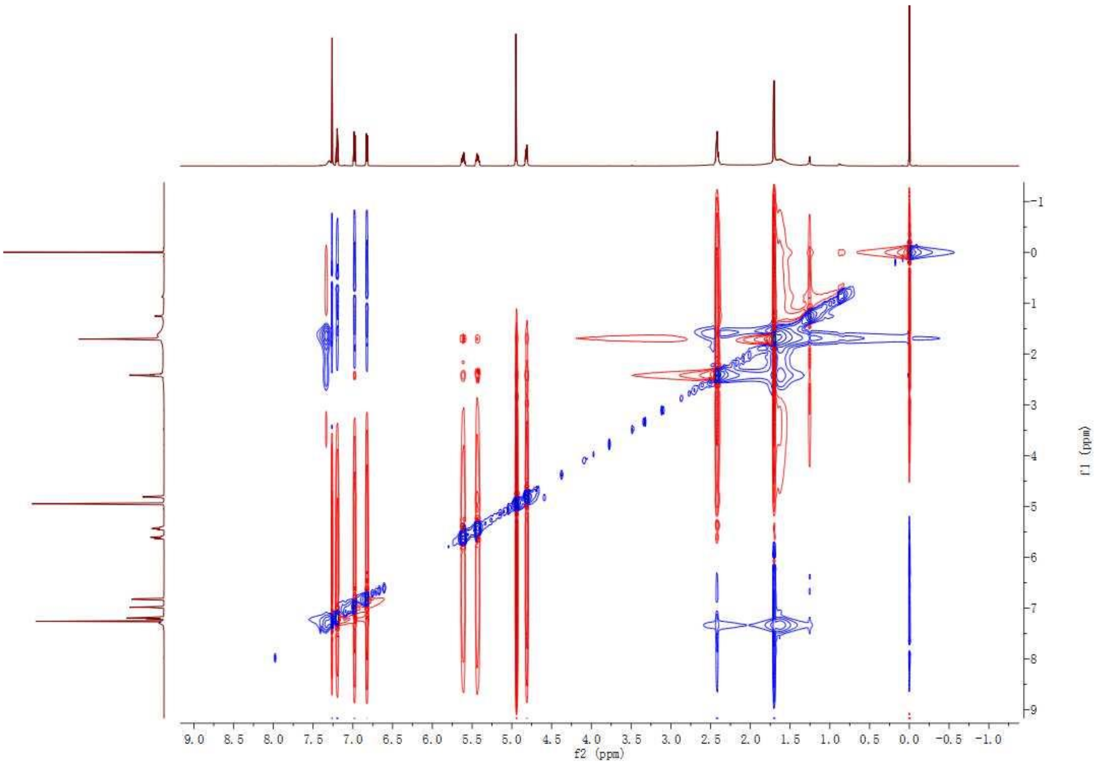

Figure S34. NOESY spectra of compound **4**.

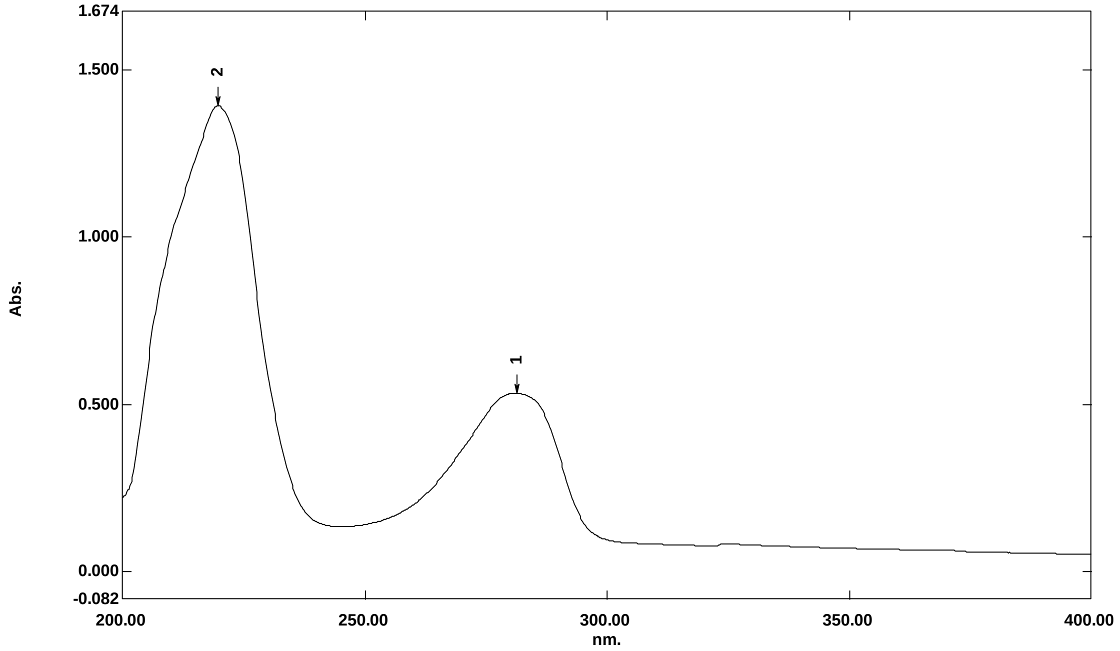

No.	波长(nm)	Abs.
1	281.40	0.533
2	219.60	1.393

Figure S35 UV spectra of compound **4**.

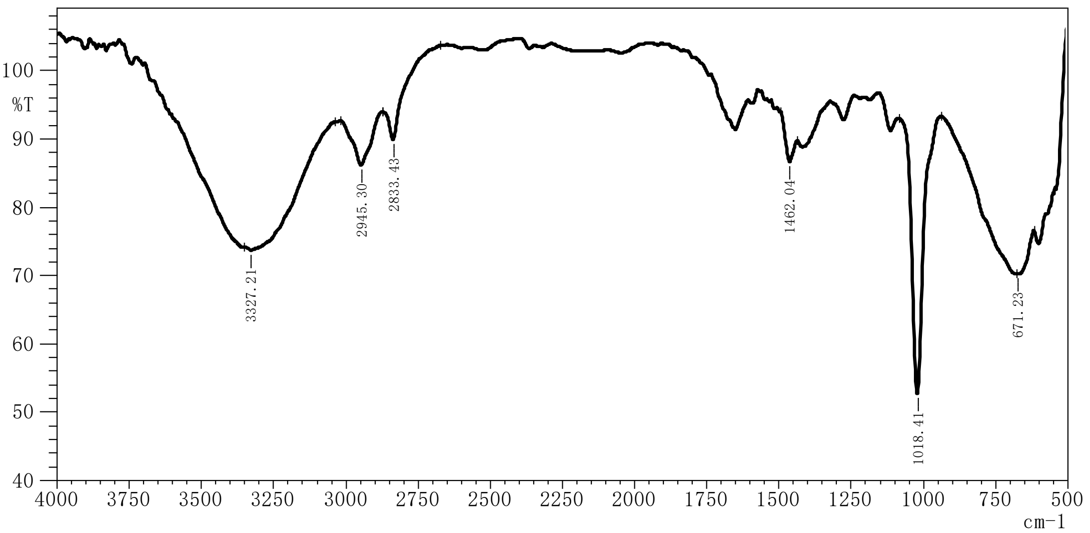

Figure S36. IR spectra of compound **4**.

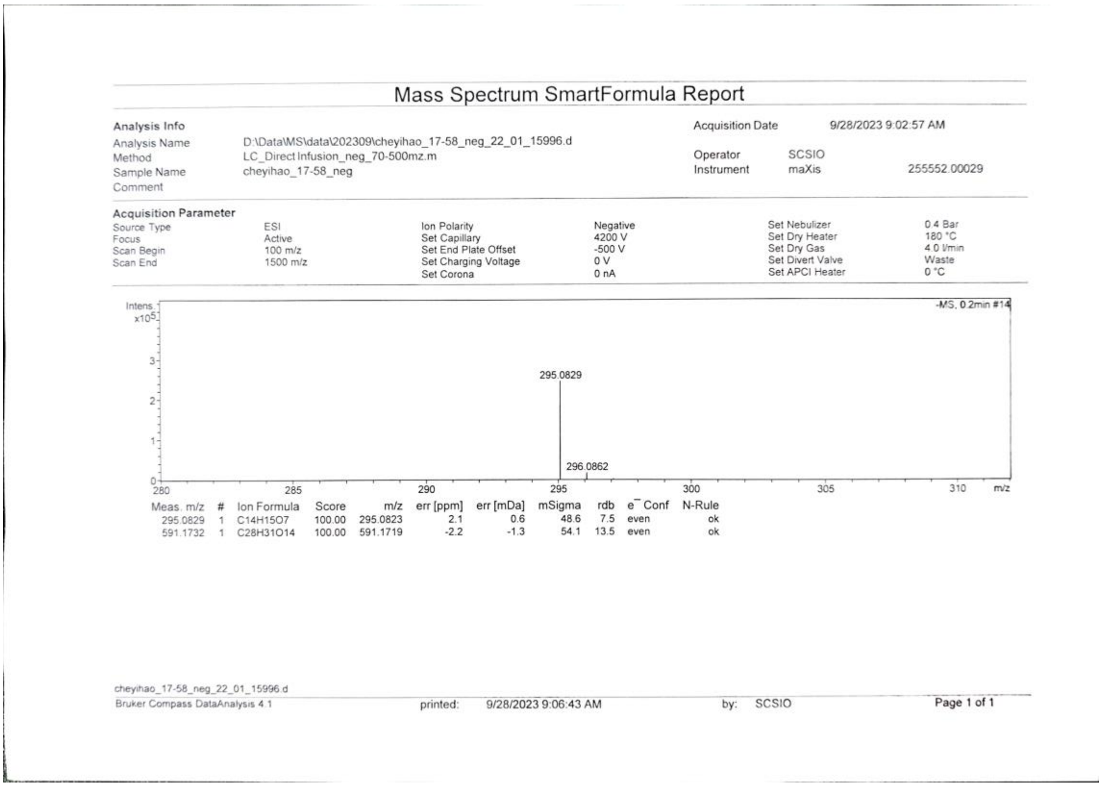

Figure S37. HR-ESI-MS spectra of compound **5**.

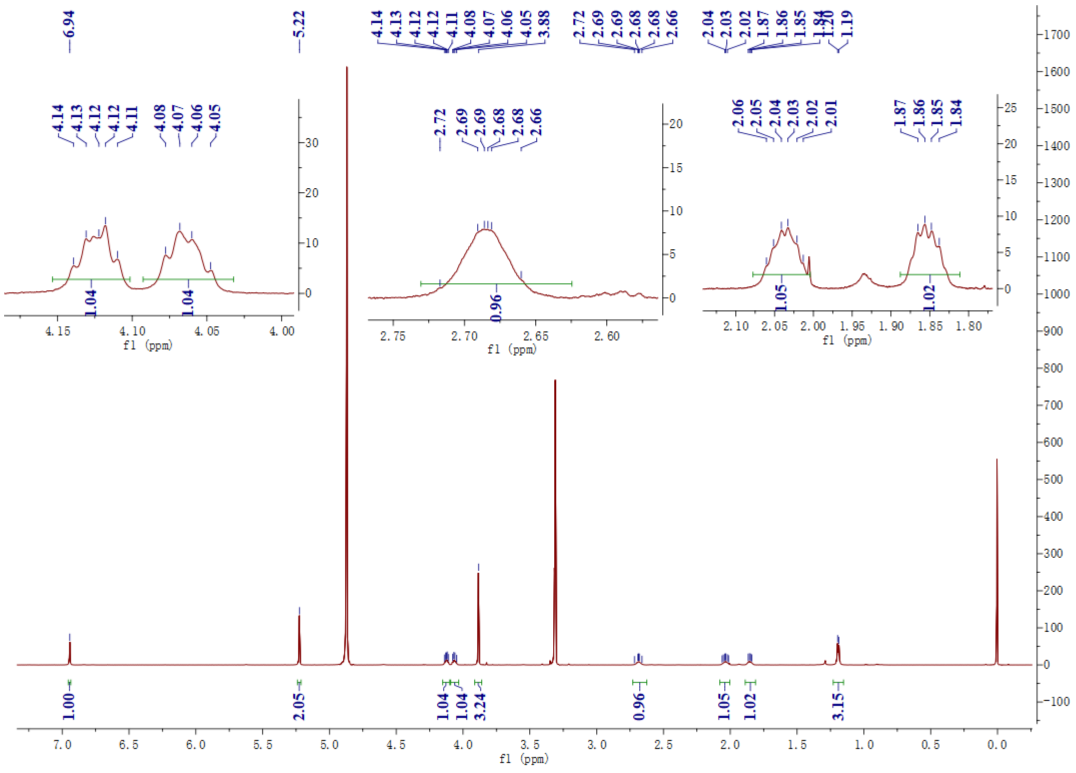

Figure S38. ^{1}H NMR spectra of compound **5**.

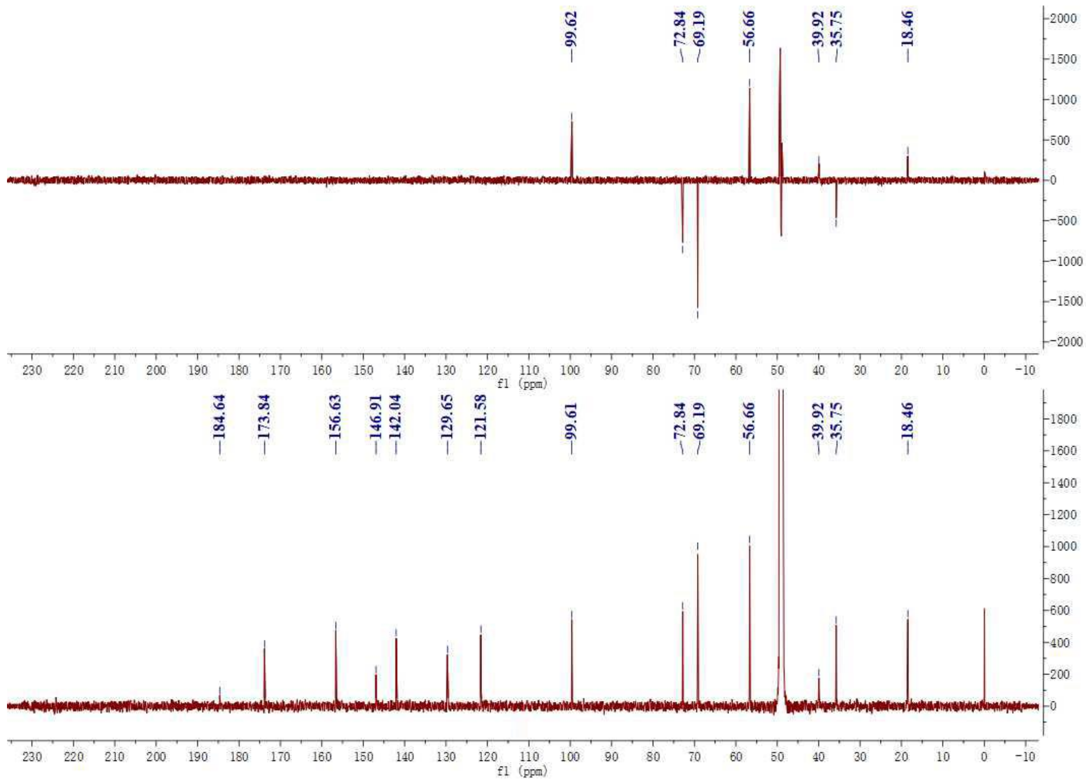

Figure S39. ^{13}C NMR and DEPT 135° spectra of compound **5**.

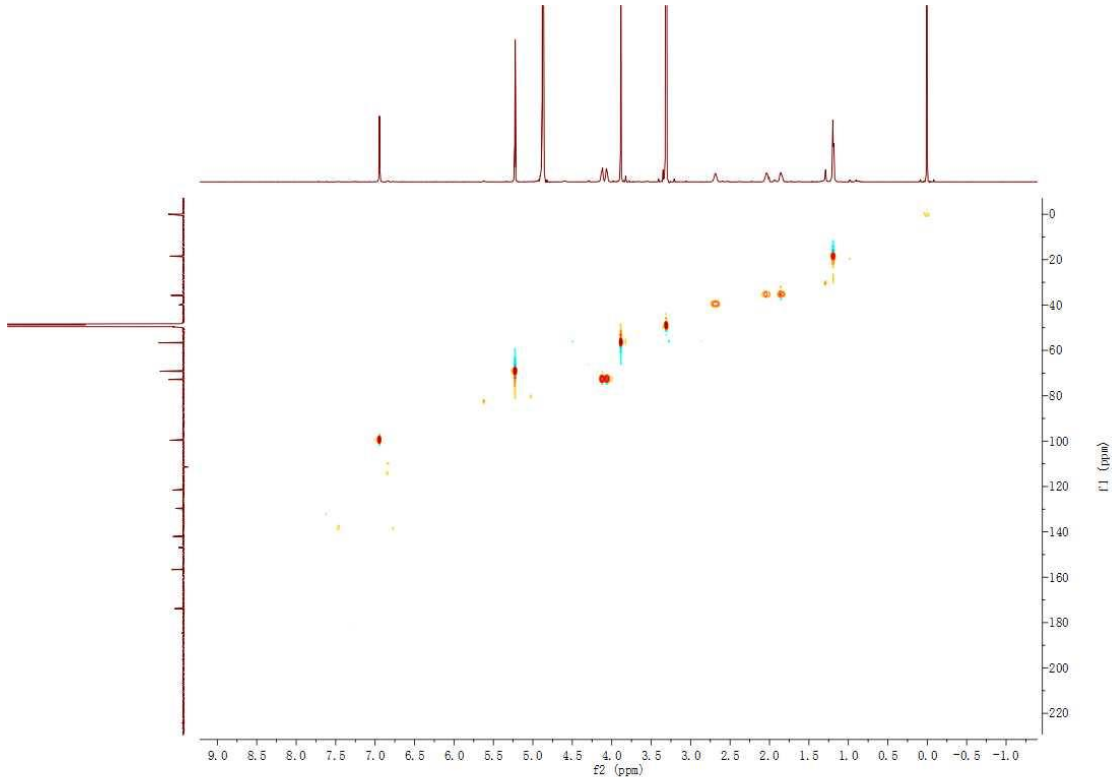

Figure S40. HSQC spectra of compound **5**.

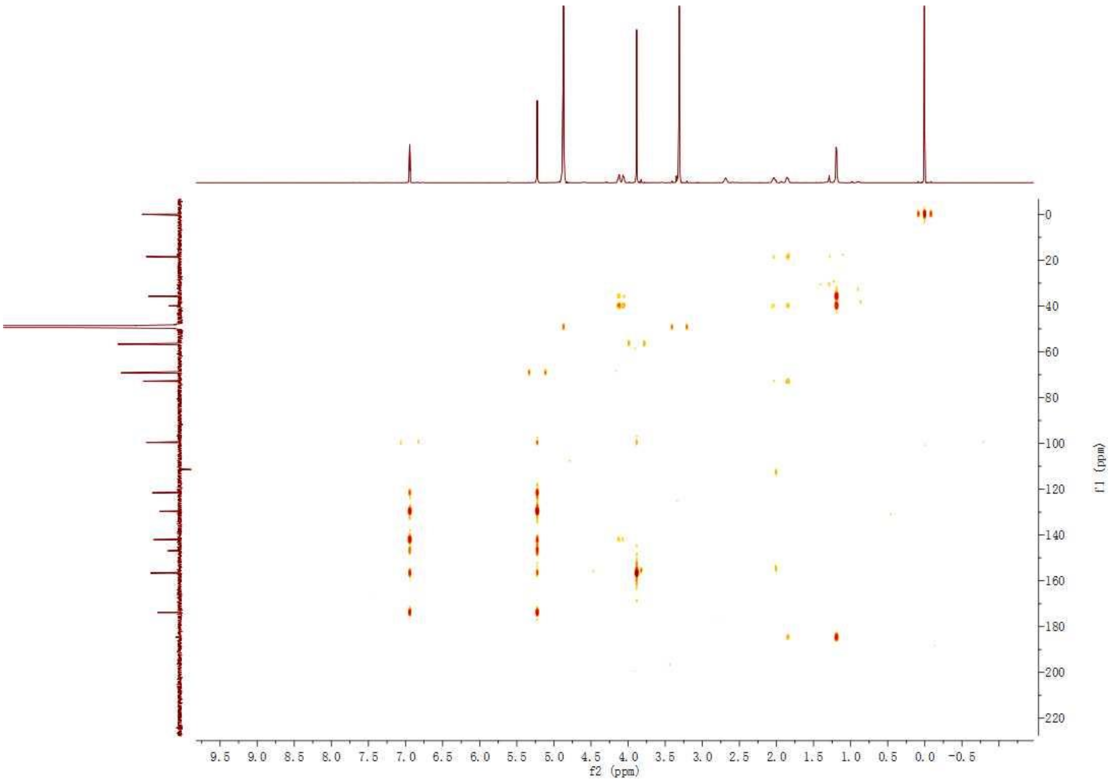

Figure S41. HMBC spectra of compound **5**.

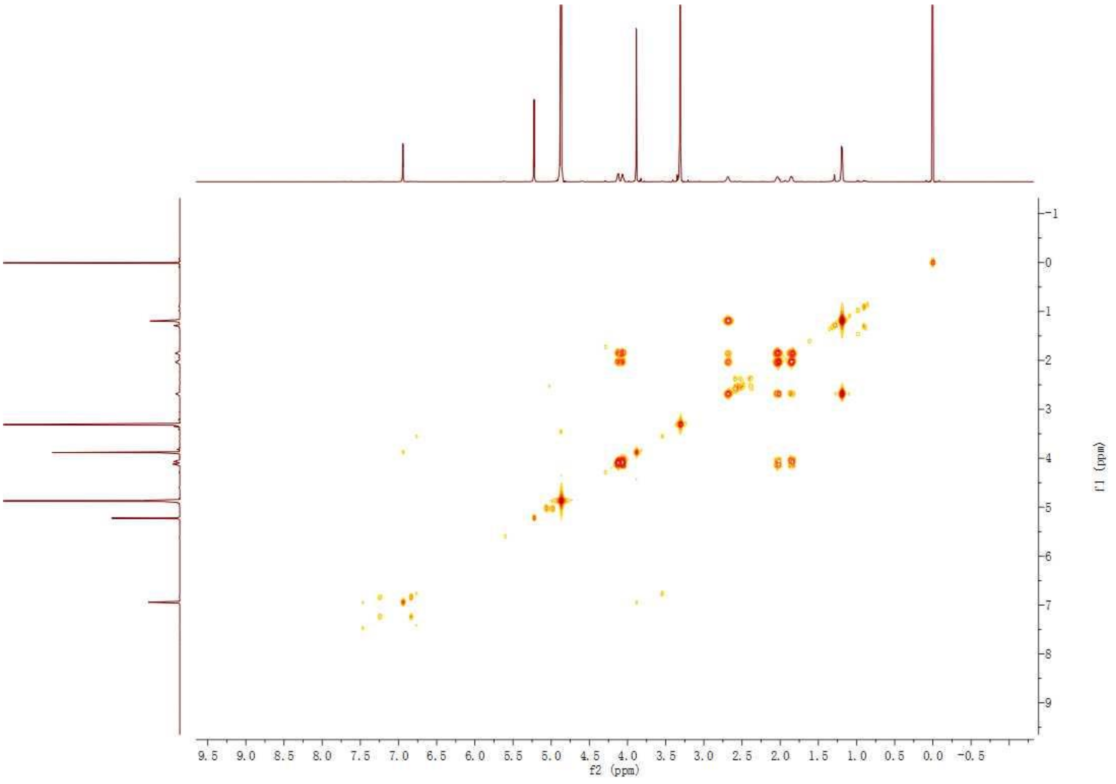

Figure S42. ^{1}H-^{1}H COSY spectra of compound **5**.

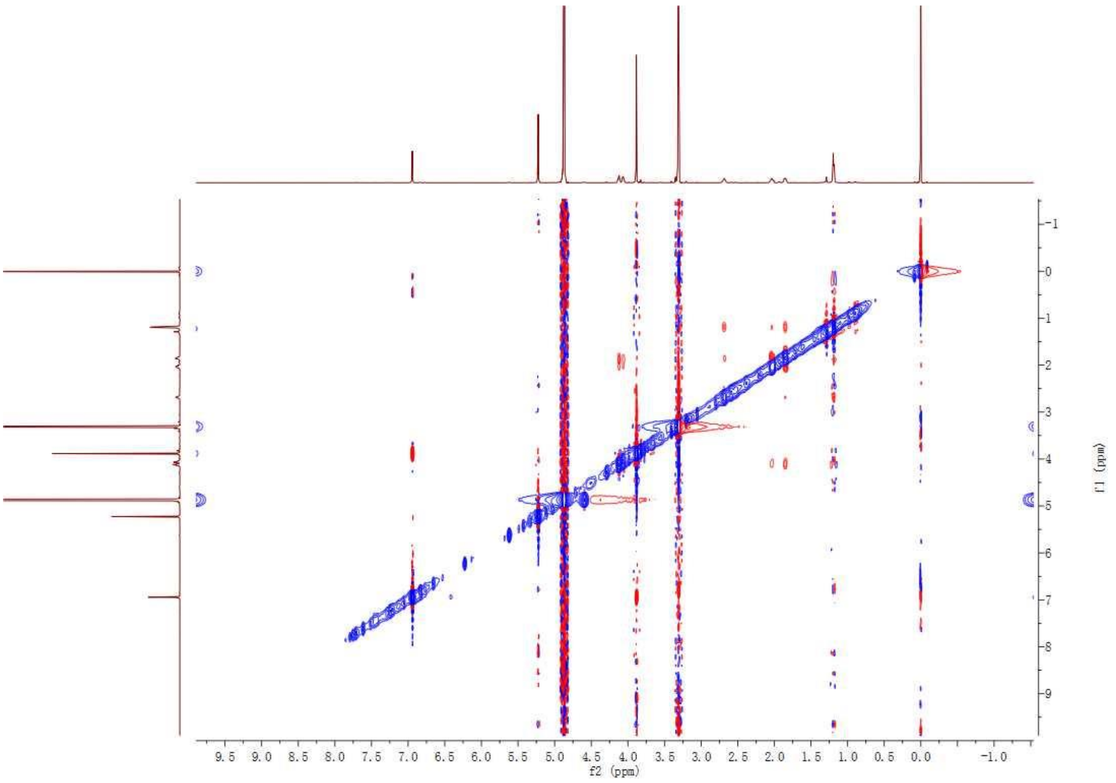

Figure S43. NOESY spectra of compound **5**.

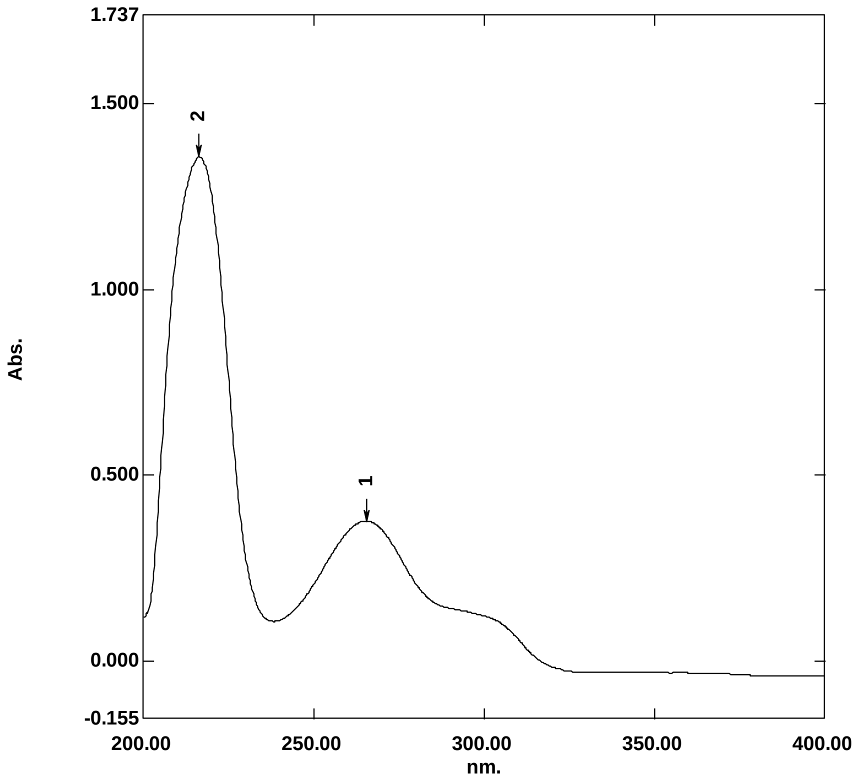

No.	波长(nm)	Abs.
1	265.60	0.377
2	216.20	1.357

Figure S44. UV spectra of compound **5**.

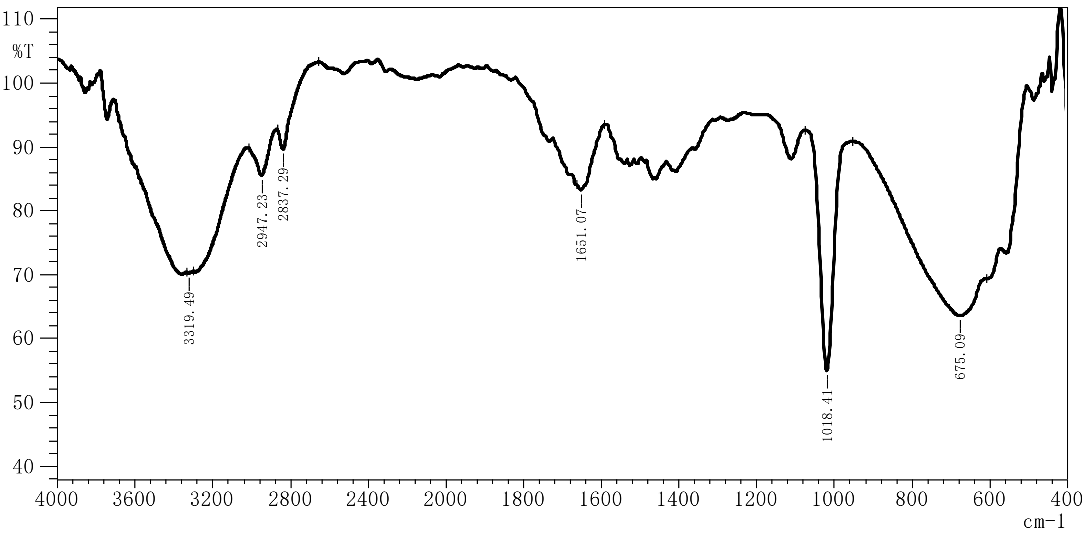

Figure S45. IR spectra of compound **5**.

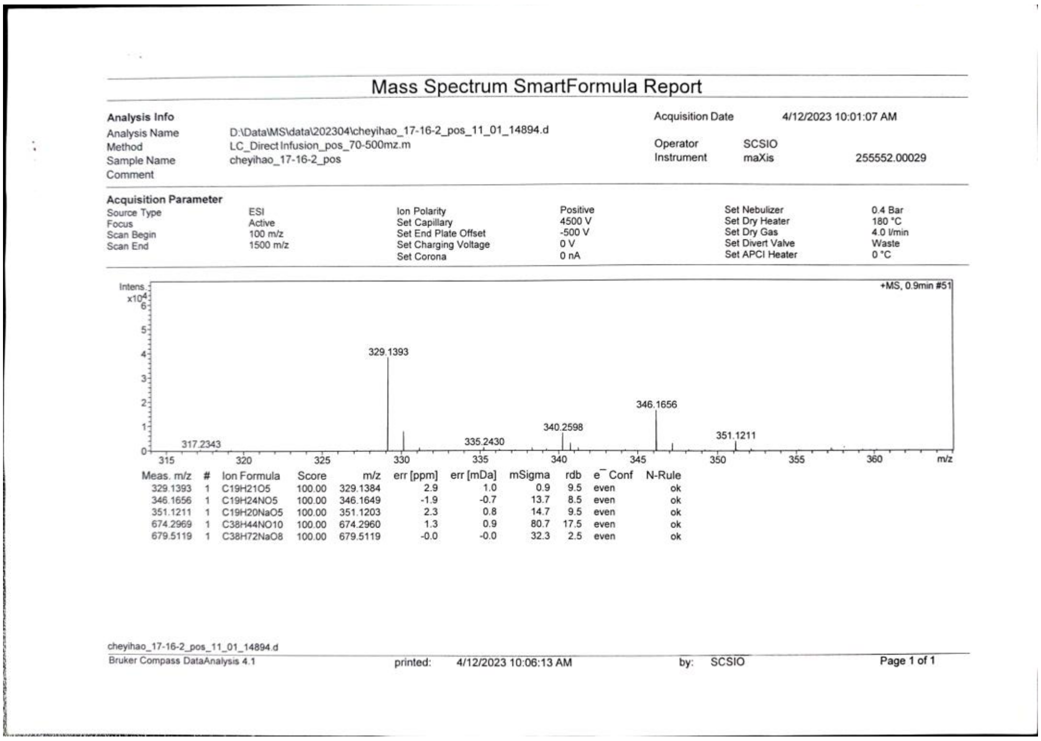

Mass Spectrum SmartFormula Report

Analysis Info

Analysis Name	D:\Data\MS\data\202304\cheyihao_17-16-2_pos_11_01_14894.d	Acquisition Date	4/12/2023 10:01:07 AM
Method	LC_Direct Infusion_pos_70-500mz.m	Operator	SCSIO
Sample Name	cheyihao_17-16-2_pos	Instrument	maXis 255552.00029
Comment			

Acquisition Parameter

Source Type	ESI	Ion Polarity	Positive	Set Nebulizer	0.4 Bar
Focus	Active	Set Capillary	4500 V	Set Dry Heater	180 °C
Scan Begin	100 m/z	Set End Plate Offset	-500 V	Set Dry Gas	4.0 l/min
Scan End	1500 m/z	Set Charging Voltage	0 V	Set Divert Valve	Waste
		Set Corona	0 nA	Set APCI Heater	0 °C

Meas. m/z	#	Ion Formula	Score	m/z	err [ppm]	err [mDa]	mSigma	rdb	e⁻ Conf	N-Rule
329.1393	1	C19H21O5	100.00	329.1384	2.9	1.0	0.9	9.5	even	ok
346.1656	1	C19H24NO5	100.00	346.1649	-1.9	-0.7	13.7	8.5	even	ok
351.1211	1	C19H20NaO5	100.00	351.1203	2.3	0.8	14.7	9.5	even	ok
674.2969	1	C38H44NO10	100.00	674.2960	1.3	0.9	80.7	17.5	even	ok
679.5119	1	C38H72NaO8	100.00	679.5119	-0.0	-0.0	32.3	2.5	even	ok

cheyihao_17-16-2_pos_11_01_14894.d

Bruker Compass DataAnalysis 4.1 printed: 4/12/2023 10:06:13 AM by: SCSIO Page 1 of 1

Figure S46. HR-ESI-MS spectra of compound **6**.

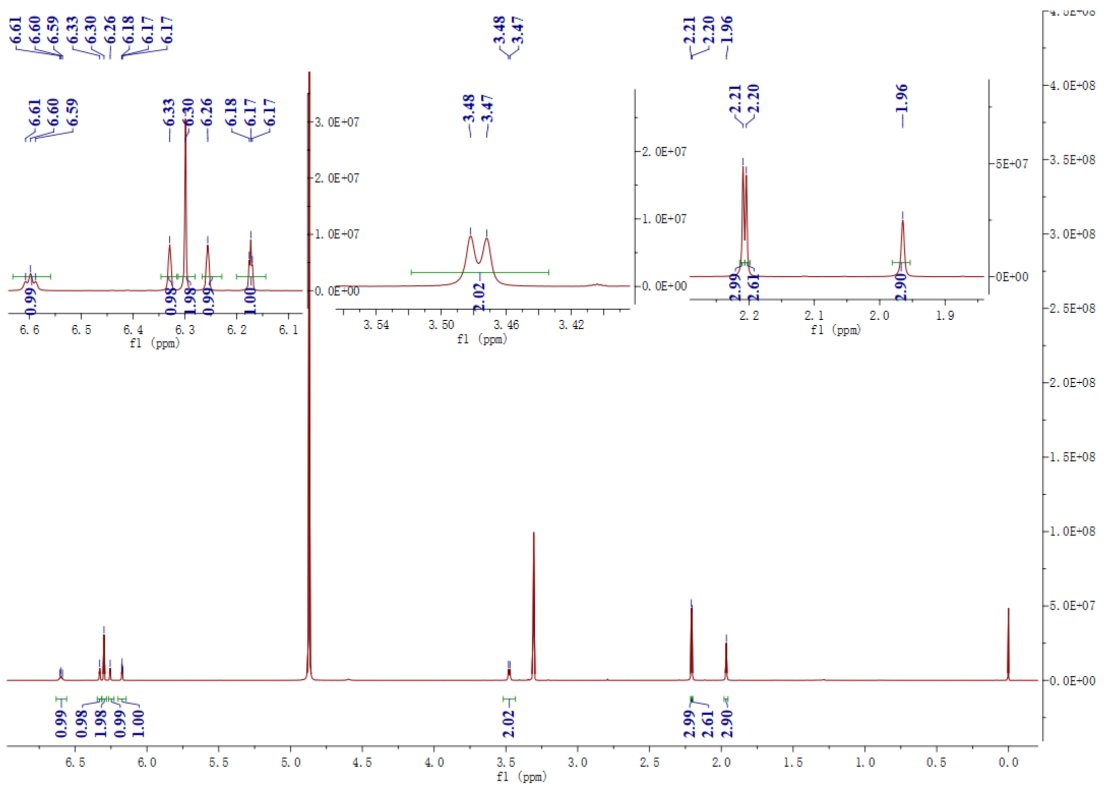

Figure S47. ^{1}H NMR spectra of compound **6**.

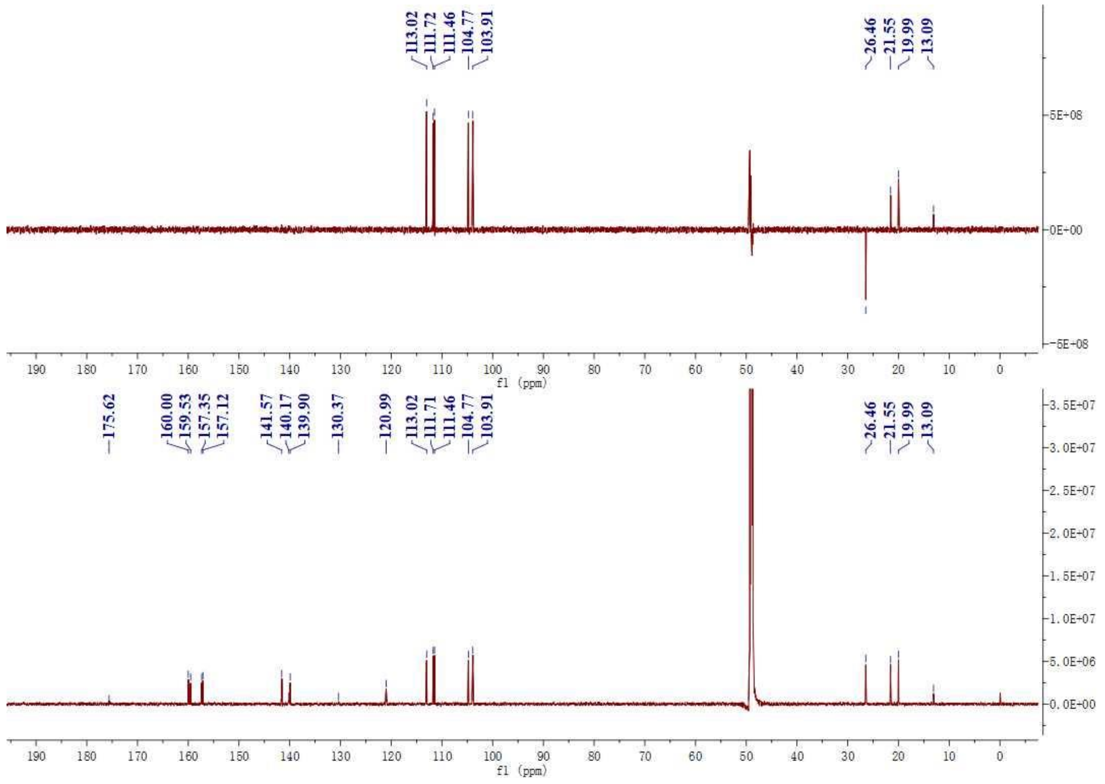

Figure S48. ^{13}C NMR and DEPT 135° spectra of compound **6**.

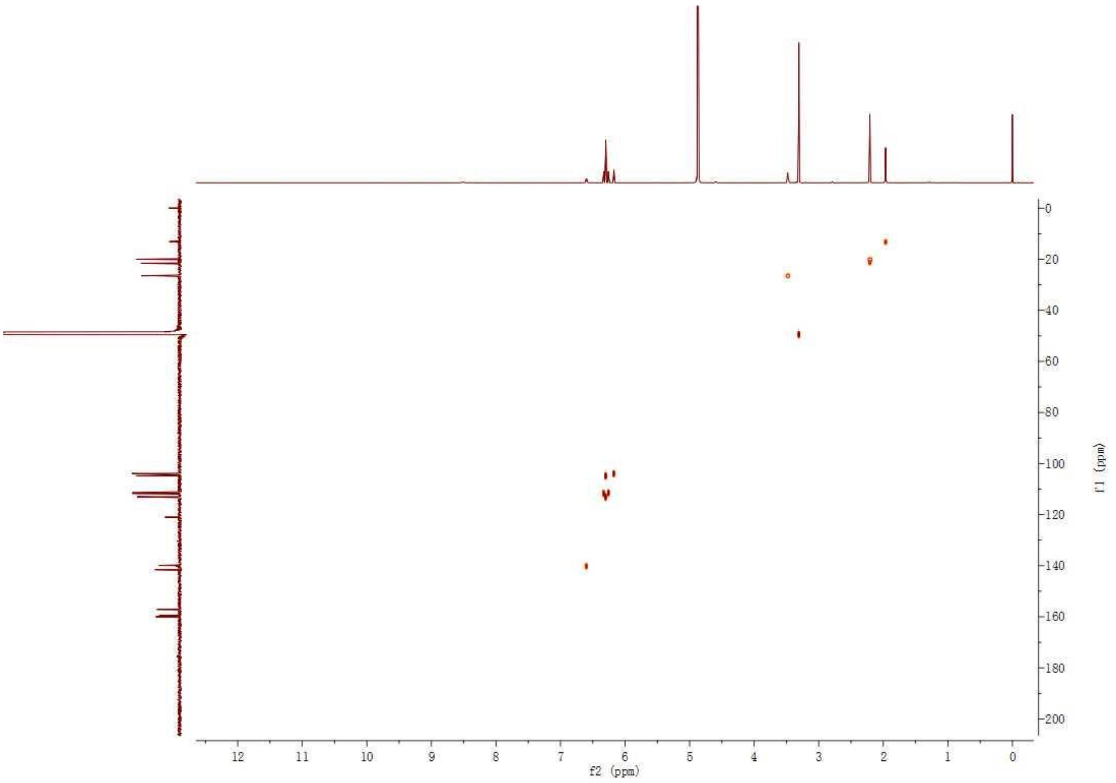

Figure S49. HSQC spectra of compound **6**.

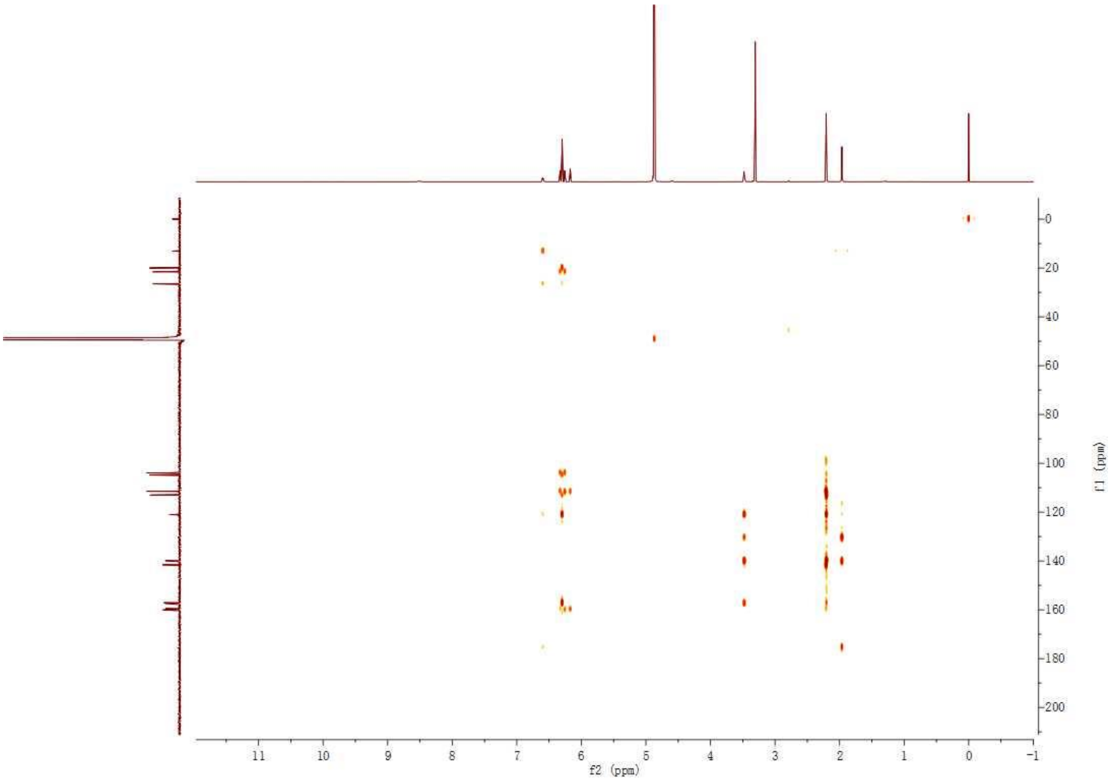

Figure S50. HMBC spectra of compound **6**.

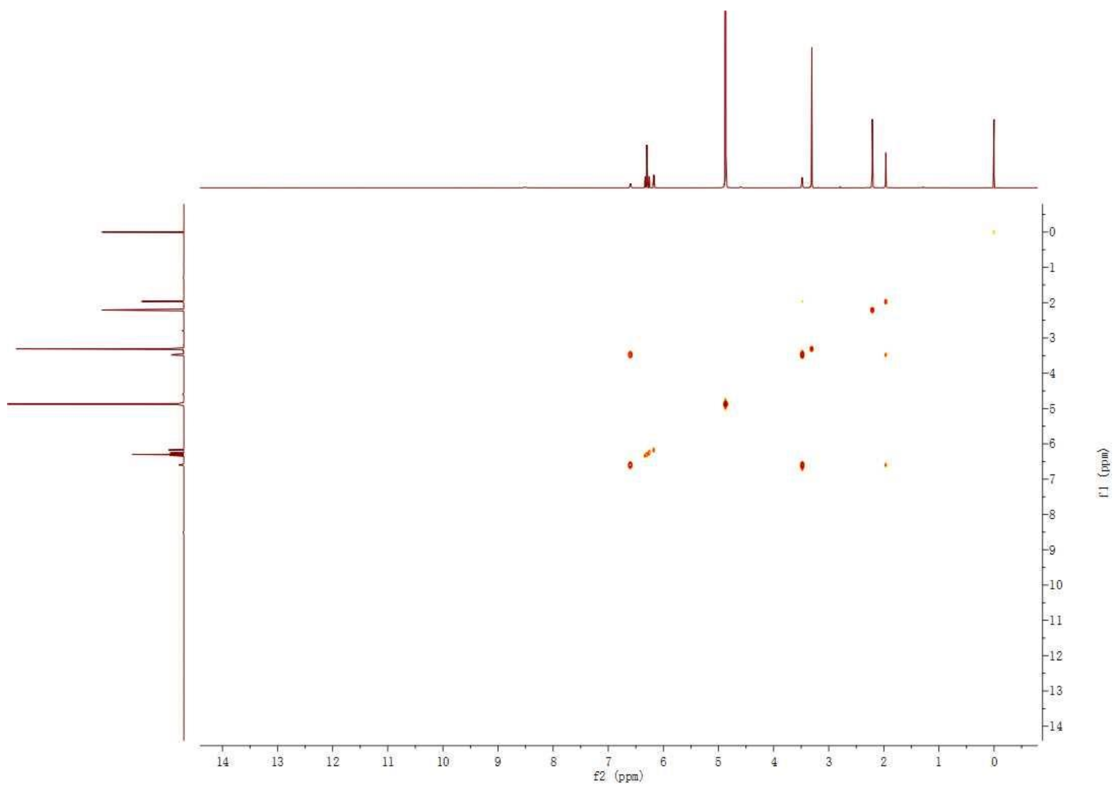

Figure S51. ^{1}H-^{1}H COSY spectra of compound **6**.

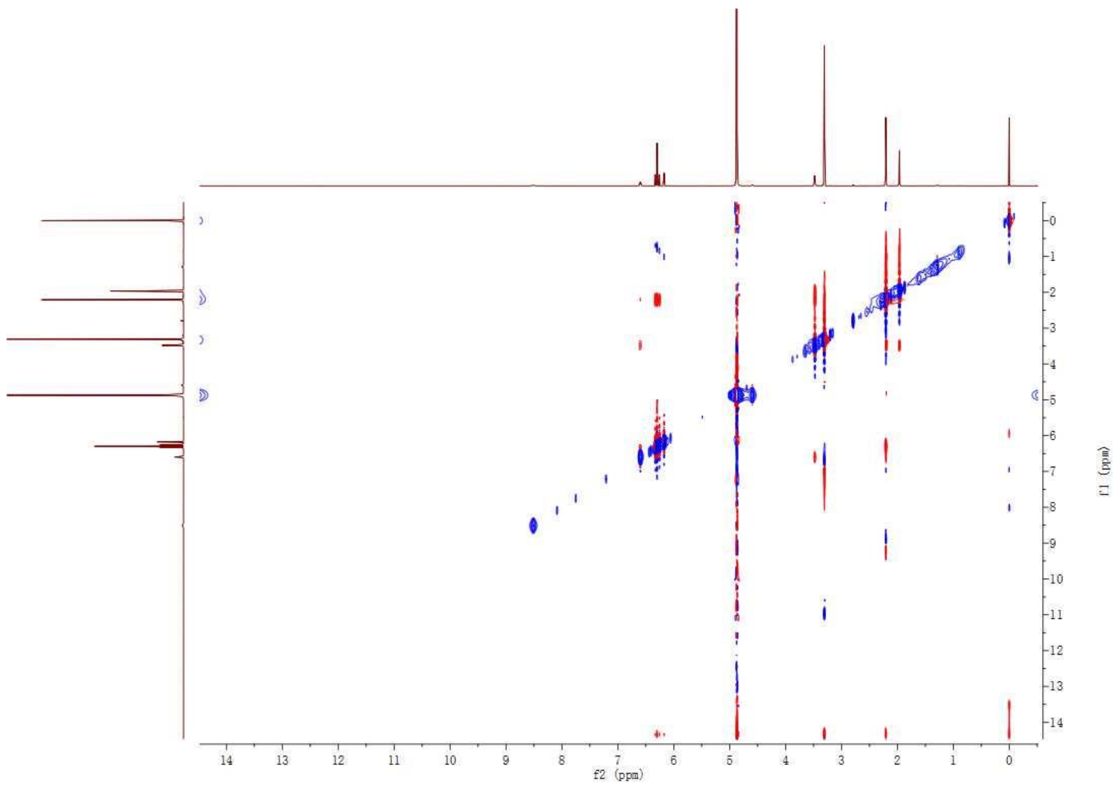

Figure S52. NOESY spectra of compound **6**.

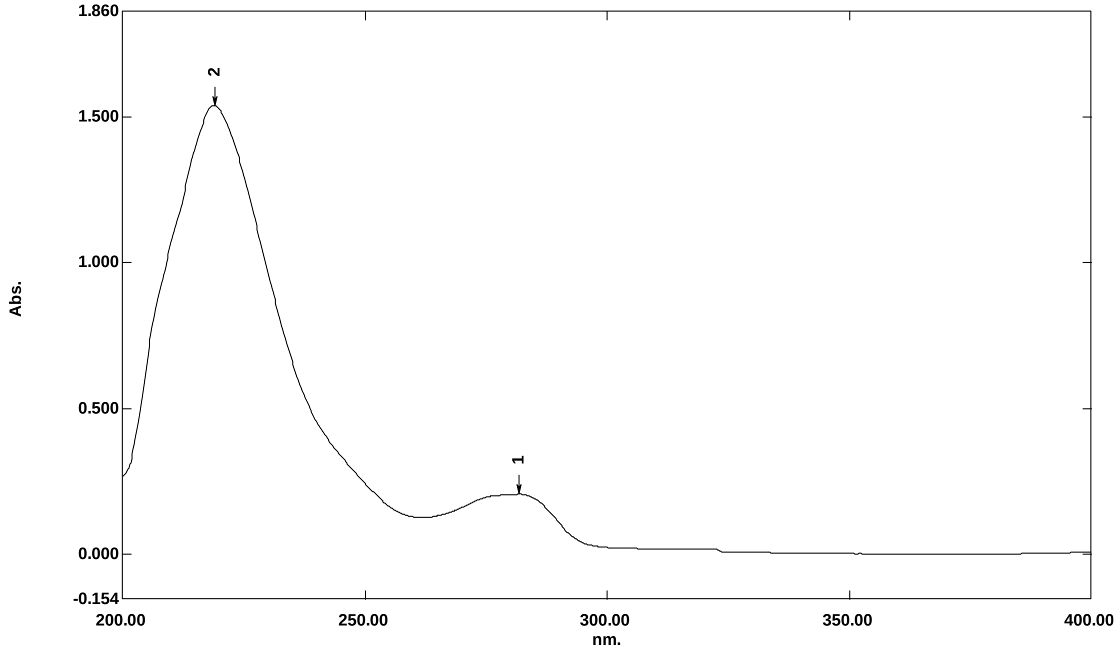

No.	波长(nm)	Abs.
1	281.80	0.206
2	219.00	1.537

Figure S53. UV spectra of compound **6**.

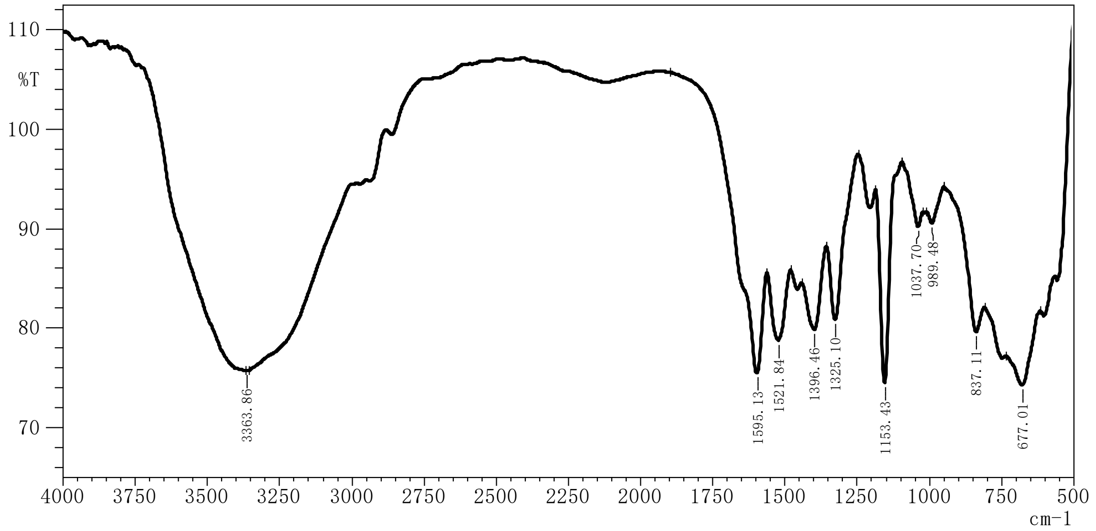

Figure S54. IR spectra of compound **6**.

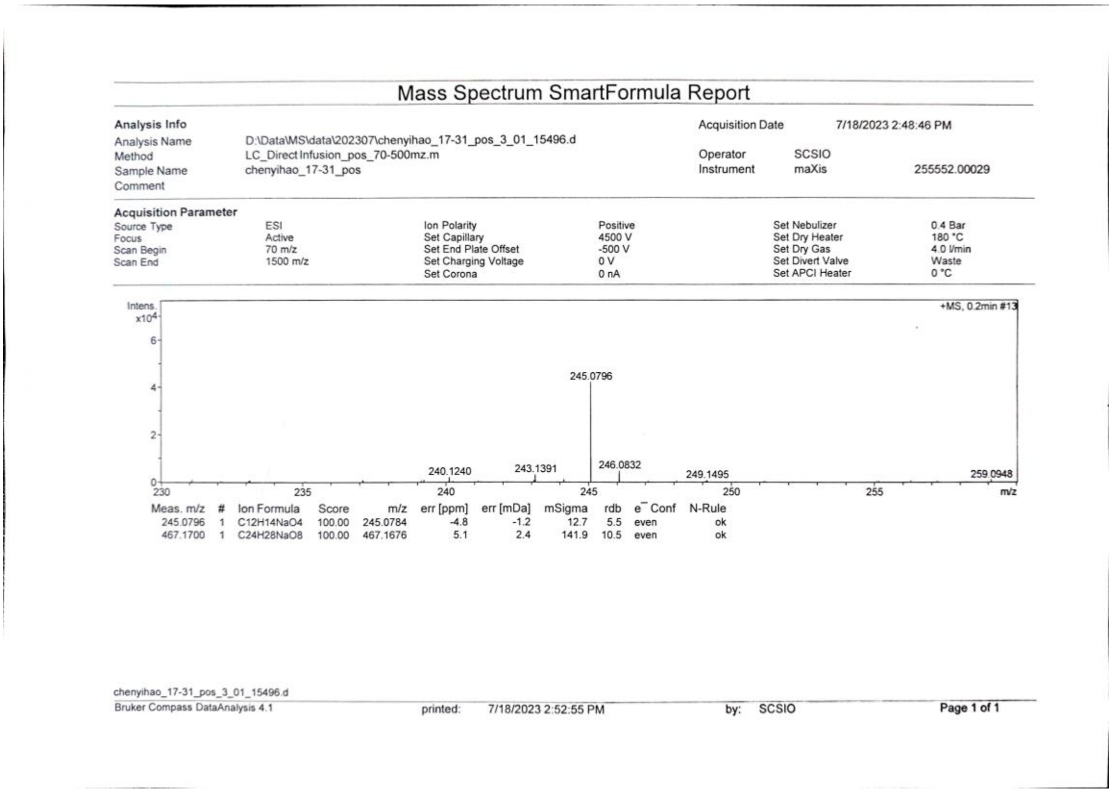

Mass Spectrum SmartFormula Report

Analysis Info			
Analysis Name	D:\Data\MS\data\202307\chenyihao_17-31_pos_3_01_15496.d	Acquisition Date	7/18/2023 2:48:46 PM
Method	LC_Direct Infusion_pos_70-500mz.m	Operator	SCSIO
Sample Name	chenyihao_17-31_pos	Instrument	maXis 255552.00029
Comment			

Acquisition Parameter

Source Type	ESI	Ion Polarity	Positive	Set Nebulizer	0.4 Bar
Focus	Active	Set Capillary	4500 V	Set Dry Heater	180 °C
Scan Begin	70 m/z	Set End Plate Offset	-500 V	Set Dry Gas	4.0 l/min
Scan End	1500 m/z	Set Charging Voltage	0 V	Set Divert Valve	Waste
		Set Corona	0 nA	Set APCI Heater	0 °C

Meas. m/z	#	Ion Formula	Score	m/z	err [ppm]	err [mDa]	mSigma	rdb	e⁻ Conf	N-Rule
245.0796	1	C12H14NaO4	100.00	245.0784	-4.8	-1.2	12.7	5.5	even	ok
467.1700	1	C24H28NaO8	100.00	467.1676	5.1	2.4	141.9	10.5	even	ok

chenyihao_17-31_pos_3_01_15496.d
Bruker Compass DataAnalysis 4.1 printed: 7/18/2023 2:52:55 PM by: SCSIO Page 1 of 1

Figure S55. HR-ESI-MS spectra of compound **7**.

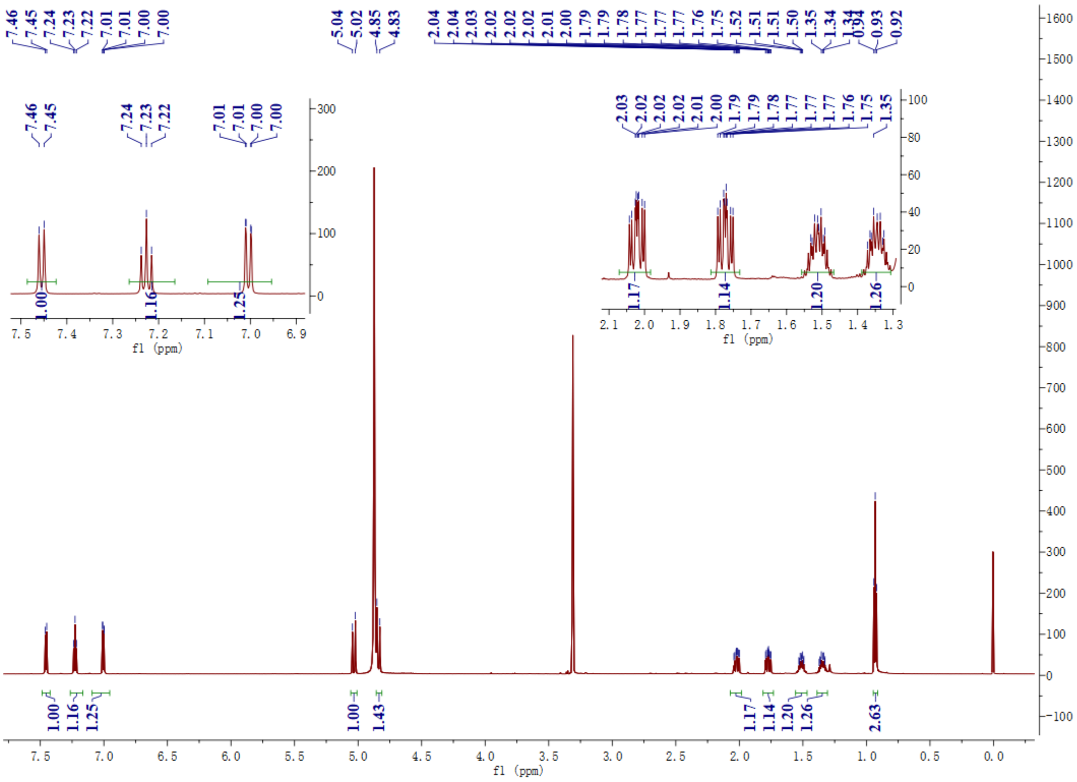

Figure S56. ^{1}H NMR spectra of compound **7**.

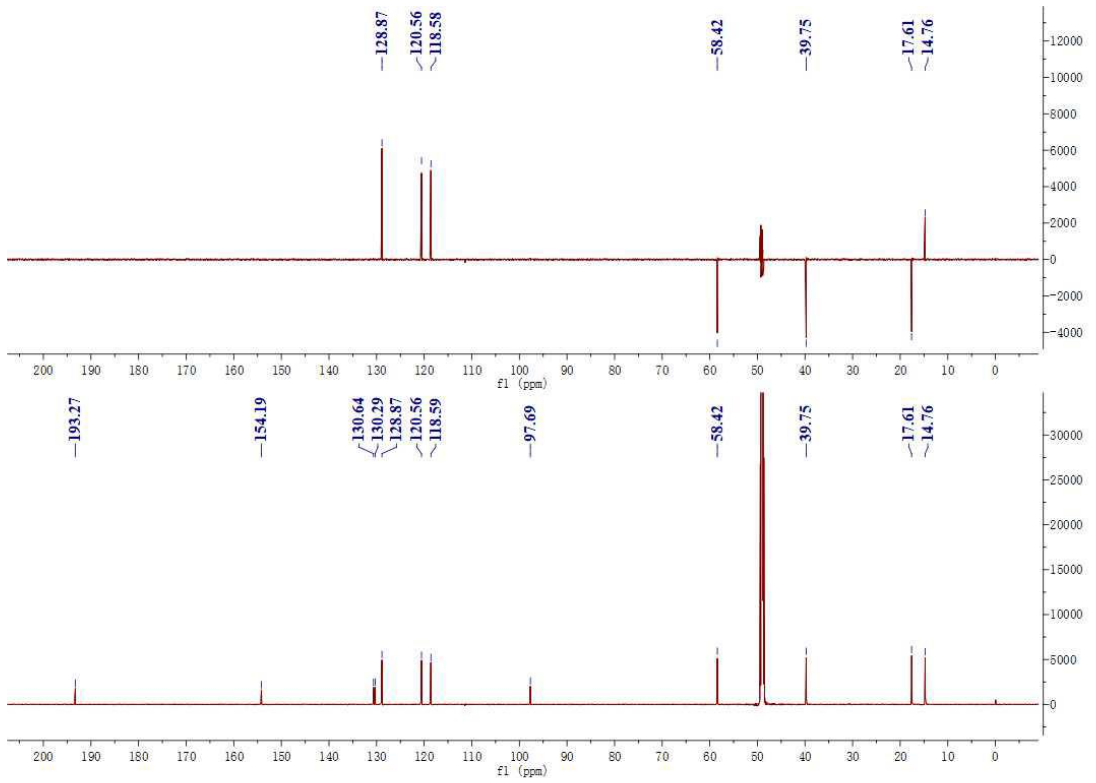

Figure S57. ^{13}C NMR and DEPT 135° spectra of compound **7**.

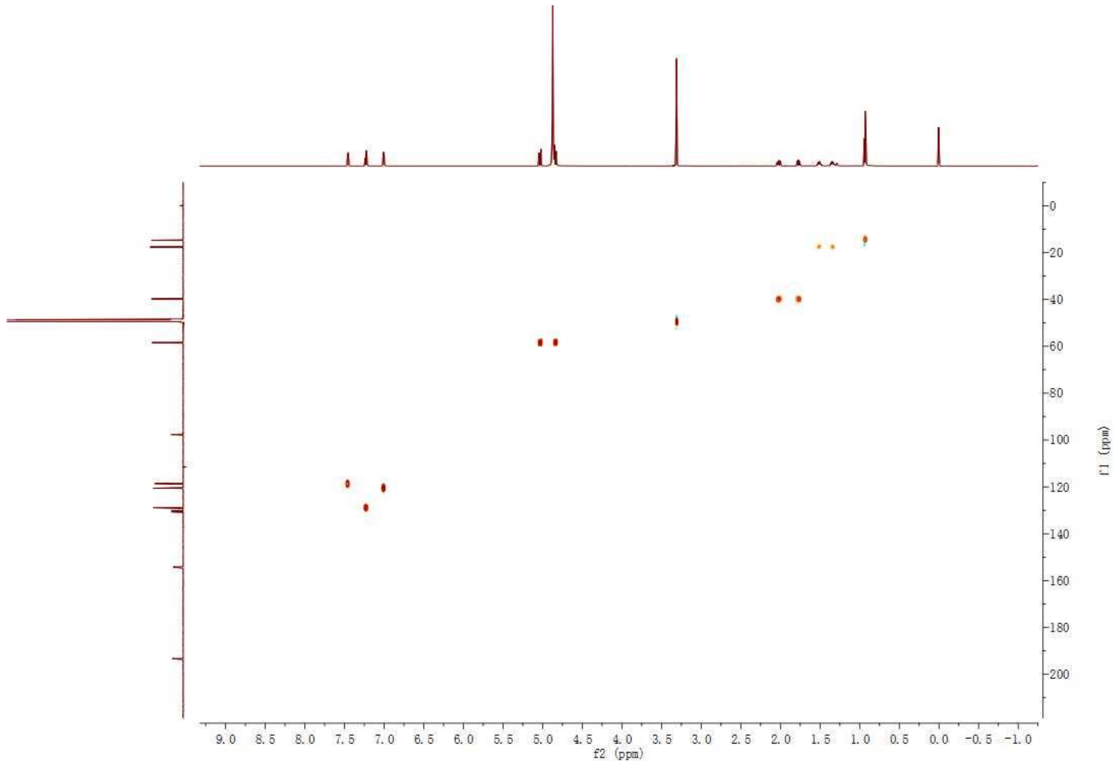

Figure S58. HSQC spectra of compound **7**.

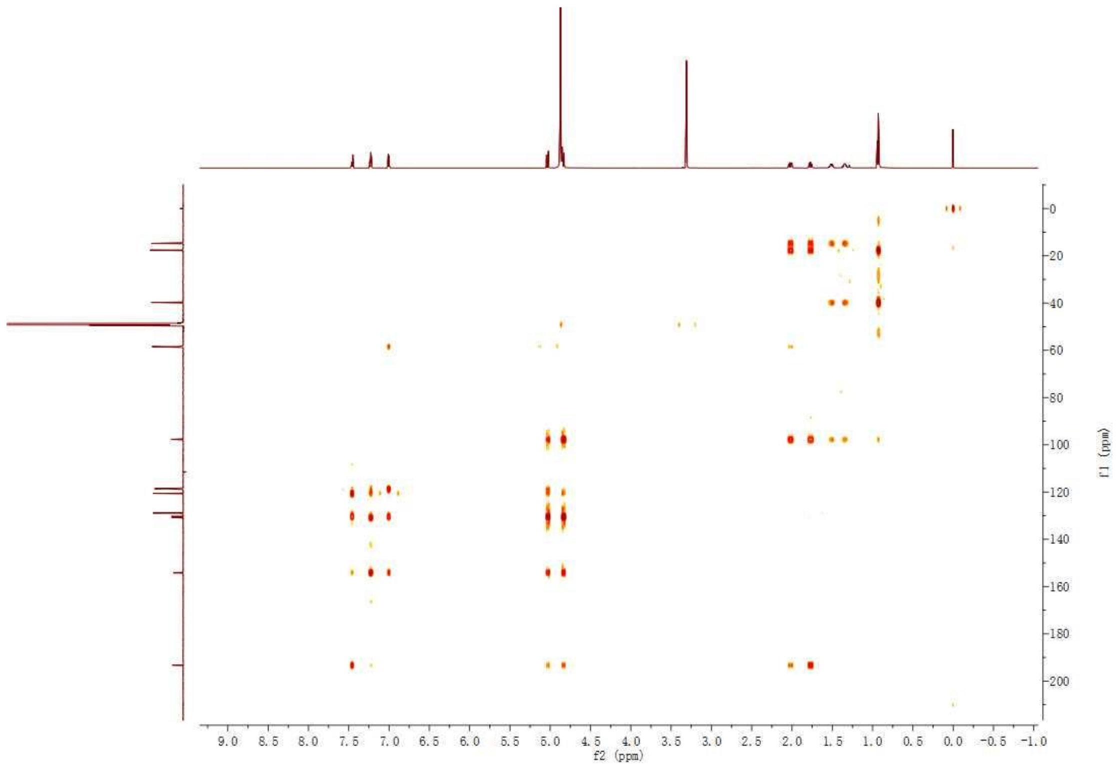

Figure S59. HMBC spectra of compound **7**.

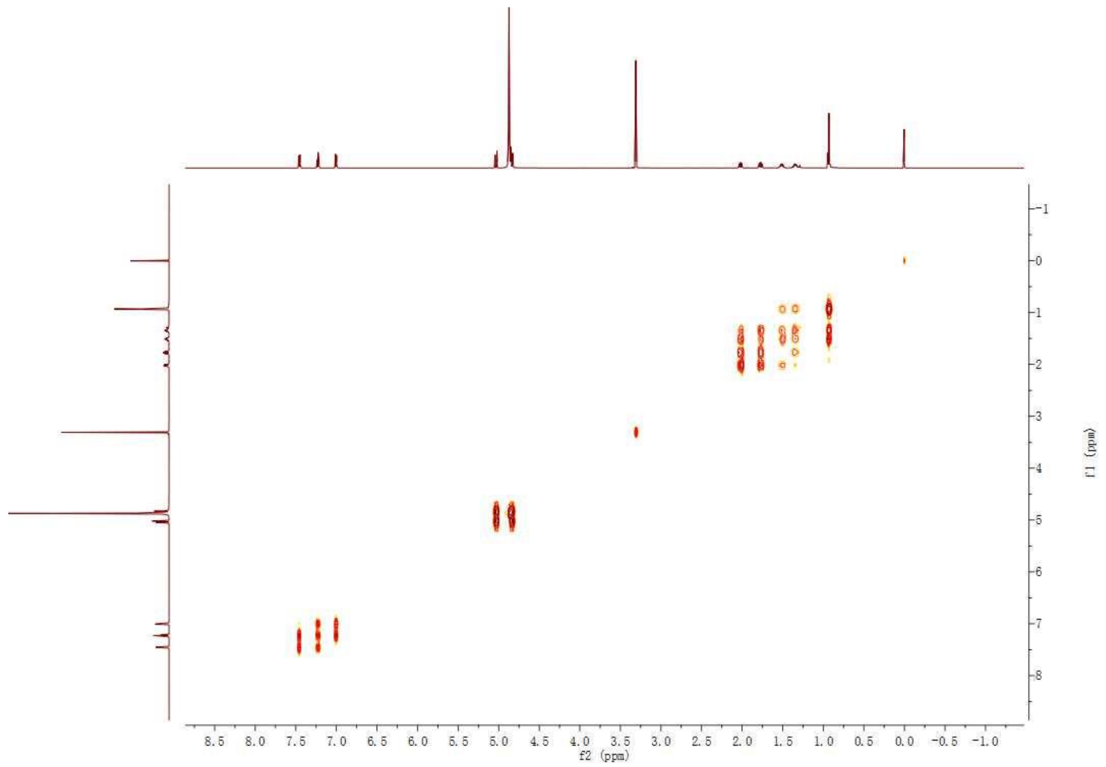

Figure S60. ^{1}H-^{1}H COSY spectra of compound **7**.

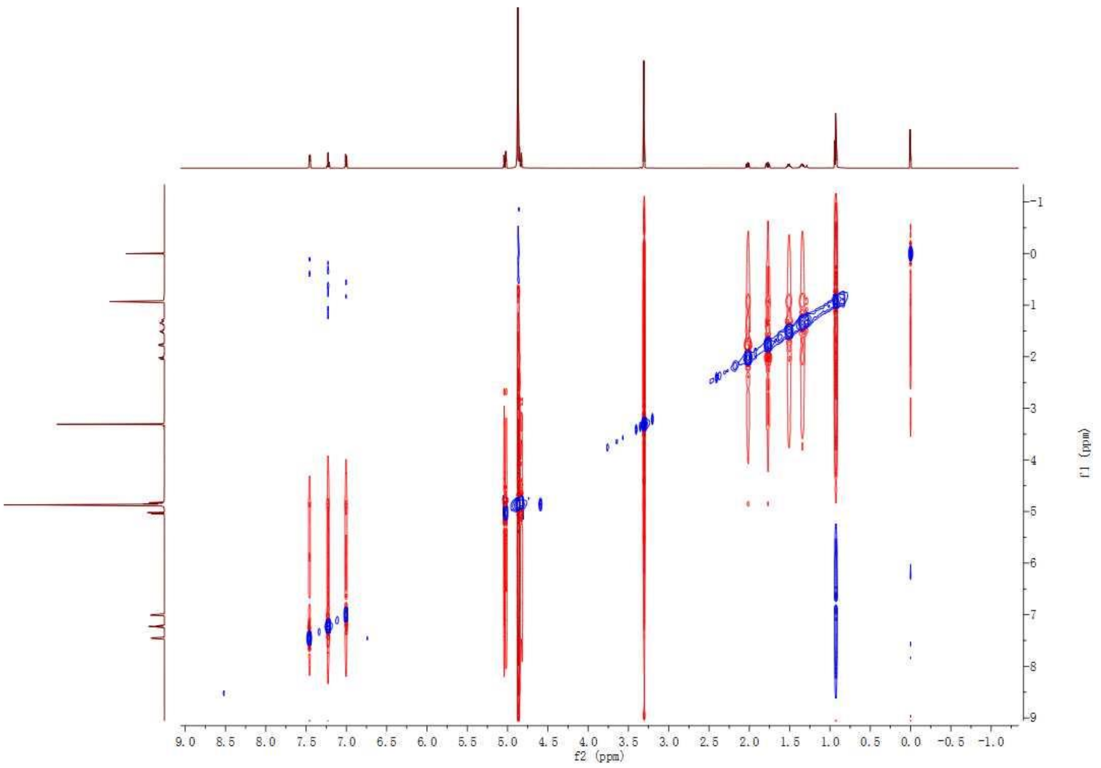

Figure S61. NOESY spectra of compound **7**.

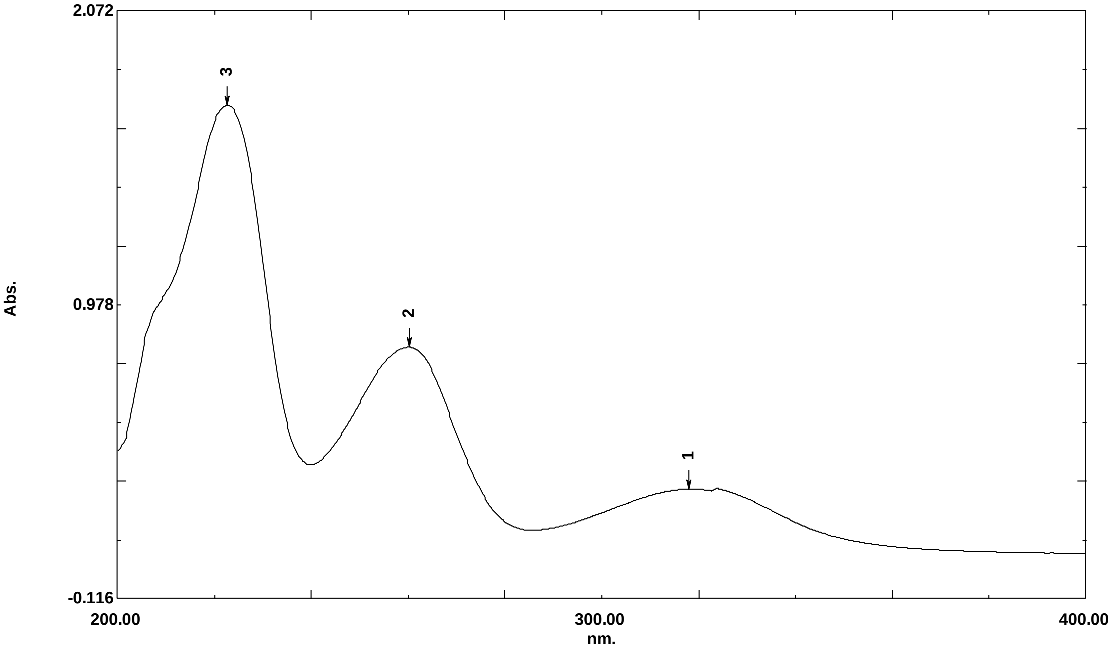

No.	波长(nm)	Abs.
1	318.00	0.292
2	260.20	0.820
3	222.60	1.722

Figure S62. UV spectra of compound **7**.

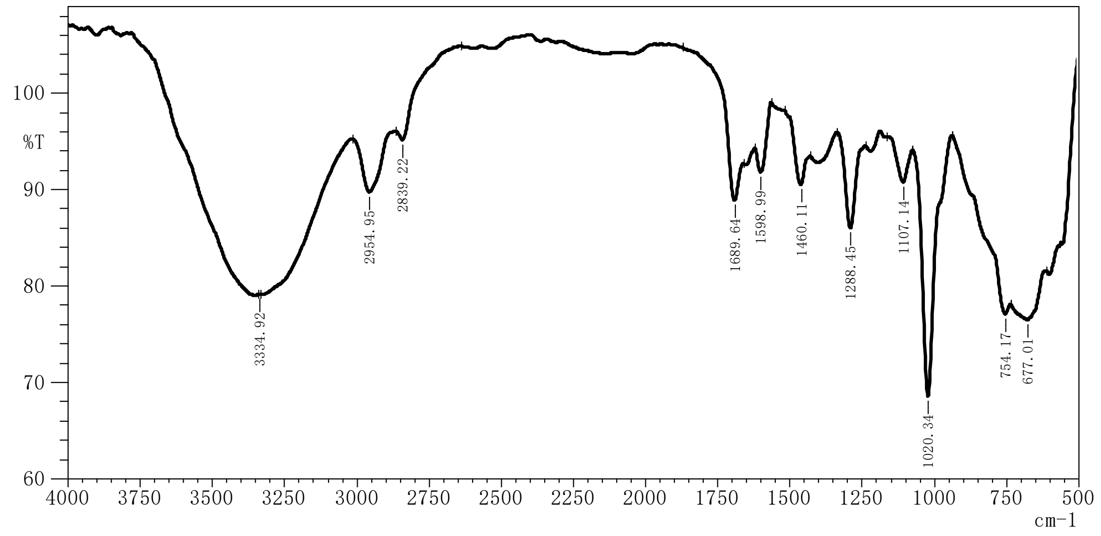

Figure S63. IR spectra of compound **7**.

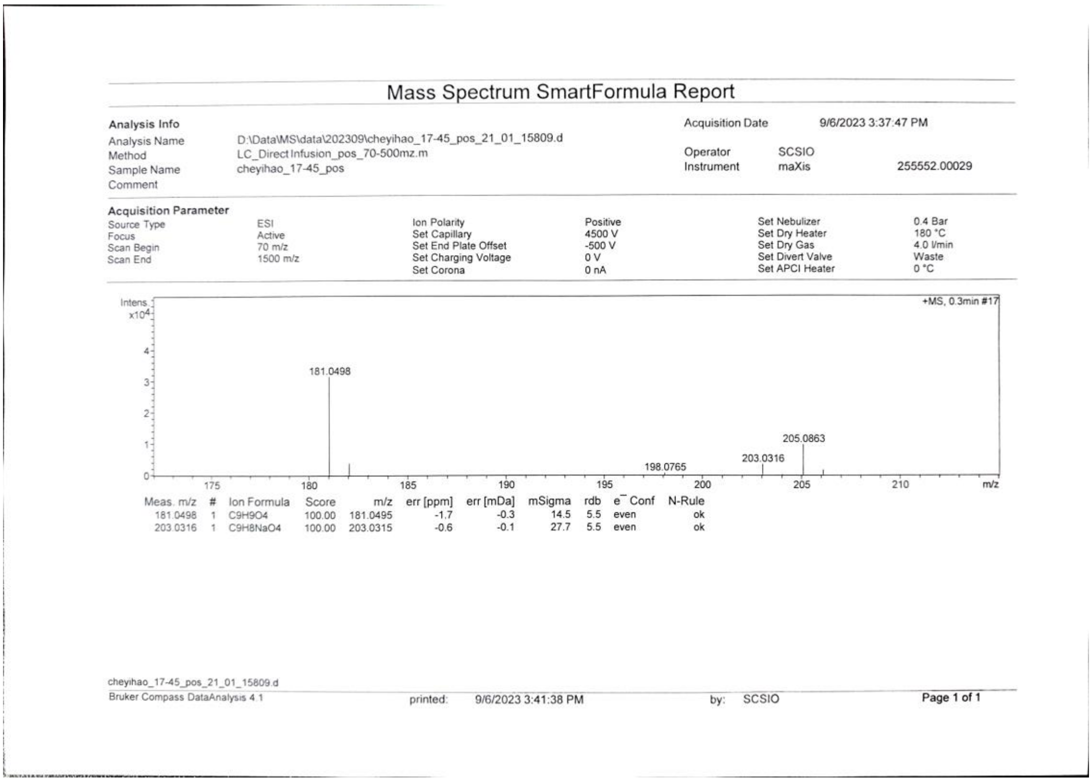

Mass Spectrum SmartFormula Report

Analysis Info

Analysis Name D:\Data\MS\data\202309\cheyihao_17-45_pos_21_01_15809.d
Method LC_Direct Infusion_pos_70-500mz.m
Sample Name cheyihao_17-45_pos
Comment

Acquisition Date 9/6/2023 3:37:47 PM
Operator SCSIO
Instrument maXis 255552.00029

Acquisition Parameter

Source Type	ESI	Ion Polarity	Positive	Set Nebulizer	0.4 Bar
Focus	Active	Set Capillary	4500 V	Set Dry Heater	180 °C
Scan Begin	70 m/z	Set End Plate Offset	-500 V	Set Dry Gas	4.0 l/min
Scan End	1500 m/z	Set Charging Voltage	0 V	Set Divert Valve	Waste
		Set Corona	0 nA	Set APCI Heater	0 °C

Meas. m/z	#	Ion Formula	Score	m/z	err [ppm]	err [mDa]	mSigma	rdb	e⁻ Conf	N-Rule
181.0498	1	C9H9O4	100.00	181.0495	-1.7	-0.3	14.5	5.5	even	ok
203.0316	1	C9H8NaO4	100.00	203.0315	-0.6	-0.1	27.7	5.5	even	ok

cheyihao_17-45_pos_21_01_15809.d
Bruker Compass DataAnalysis 4.1 printed: 9/6/2023 3:41:38 PM by: SCSIO Page 1 of 1

Figure S64. HR-ESI-MS spectra of compound **8**.

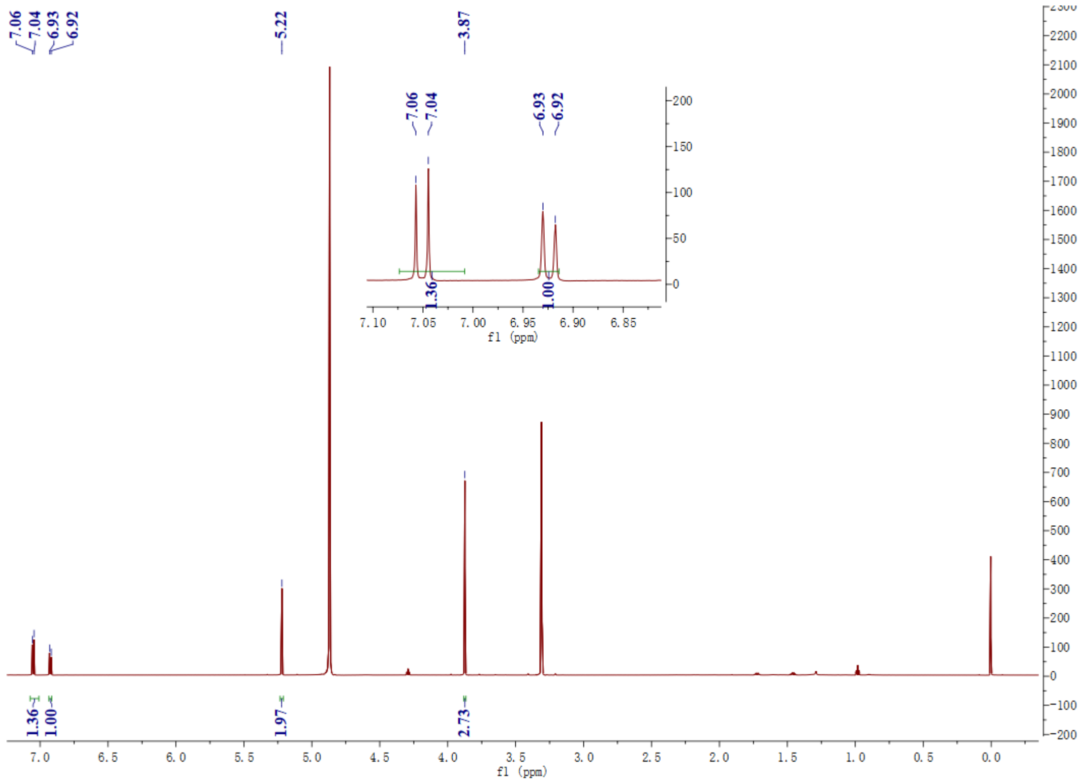

Figure S65. ^{1}H NMR spectra of compound **8**.

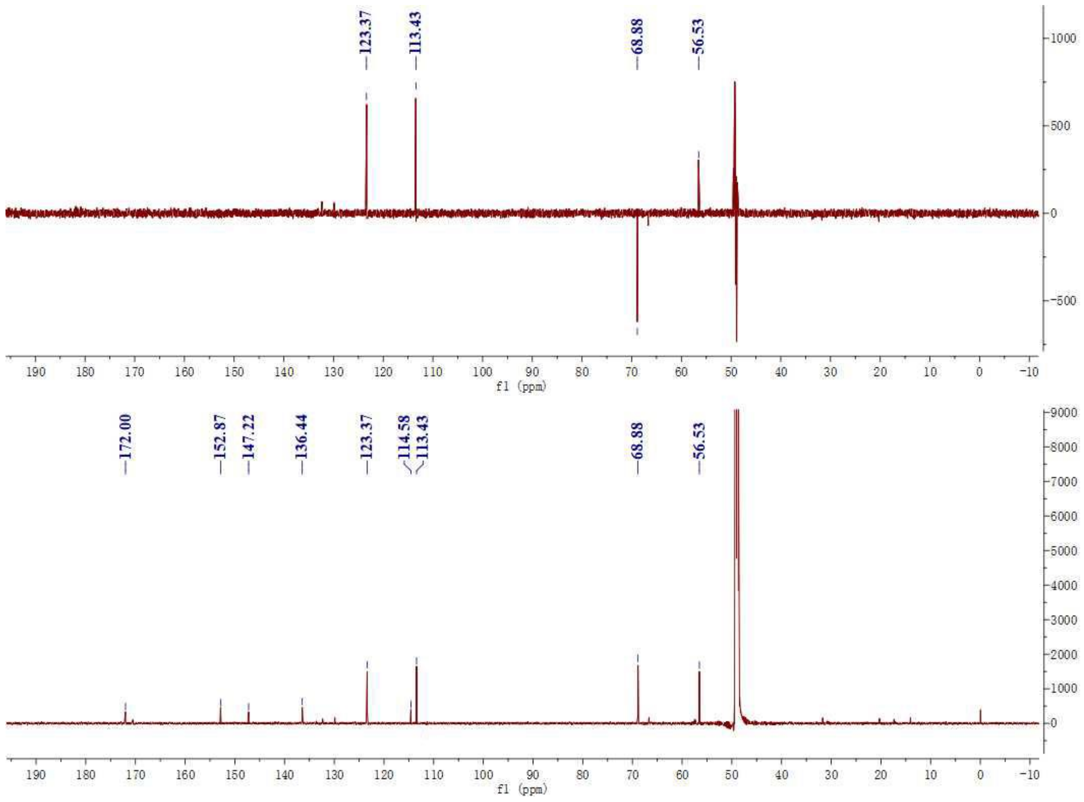

Figure S66. ^{13}C NMR and DEPT 135° spectra of compound **8**.

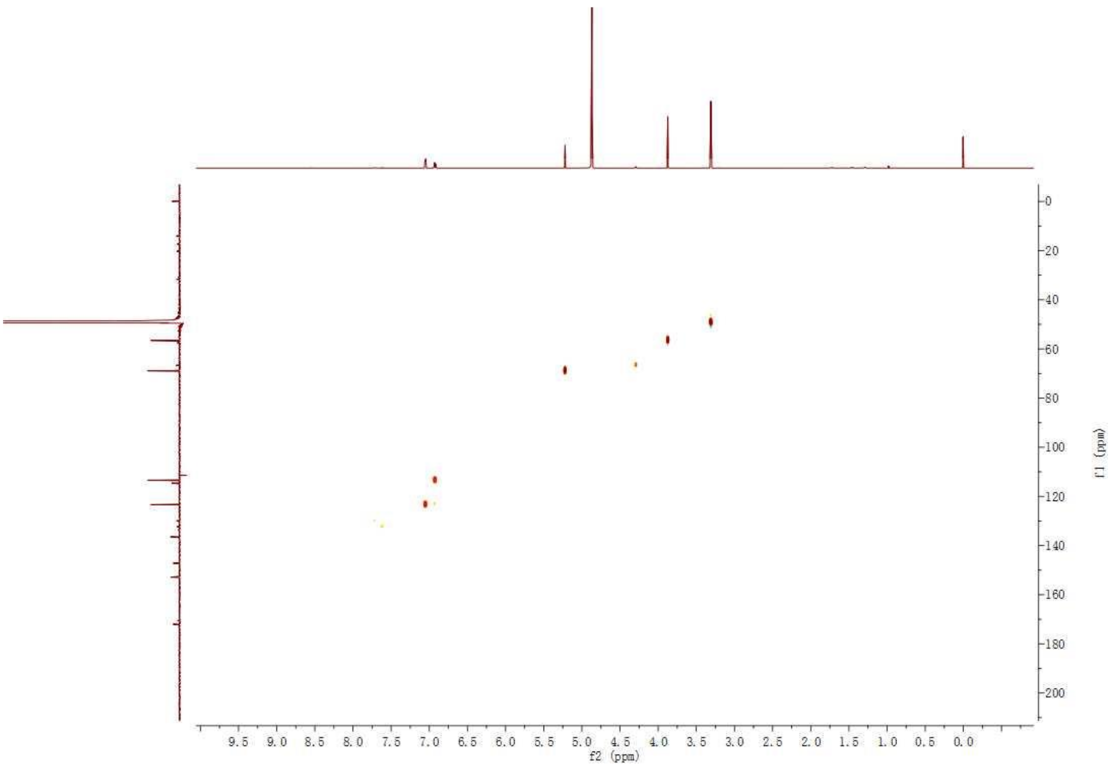

Figure S67. HSQC spectra of compound **8**.

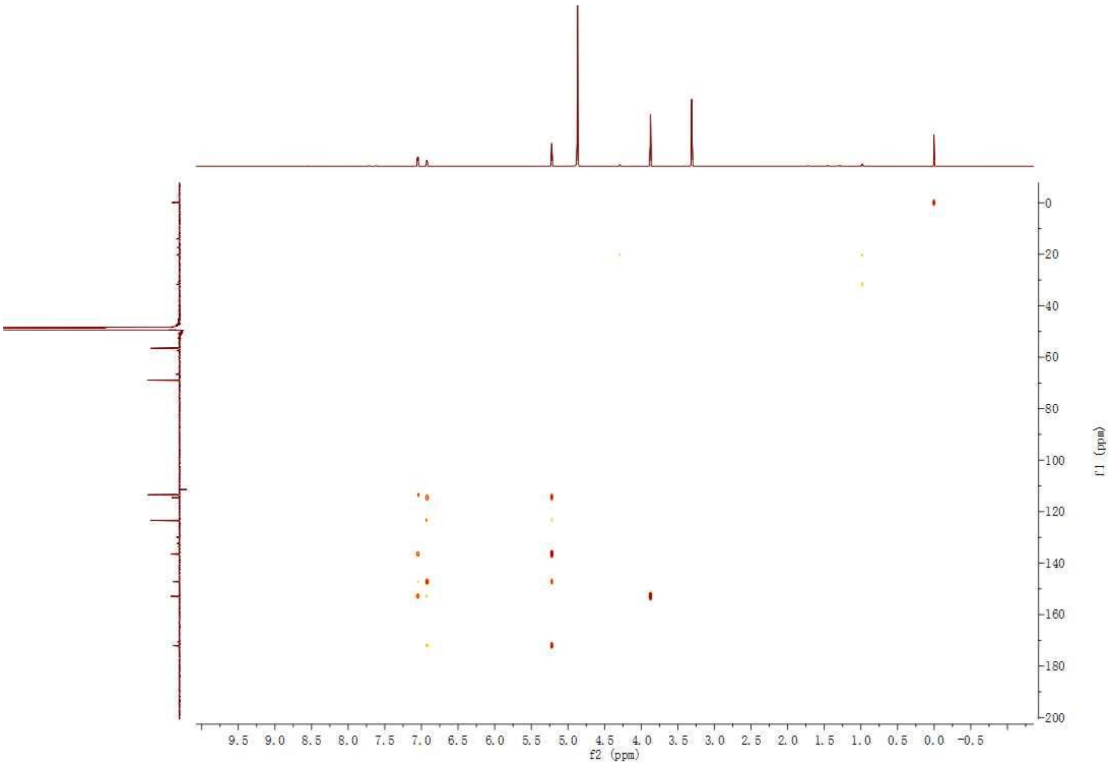

Figure S68. HMBC spectra of compound **8**.

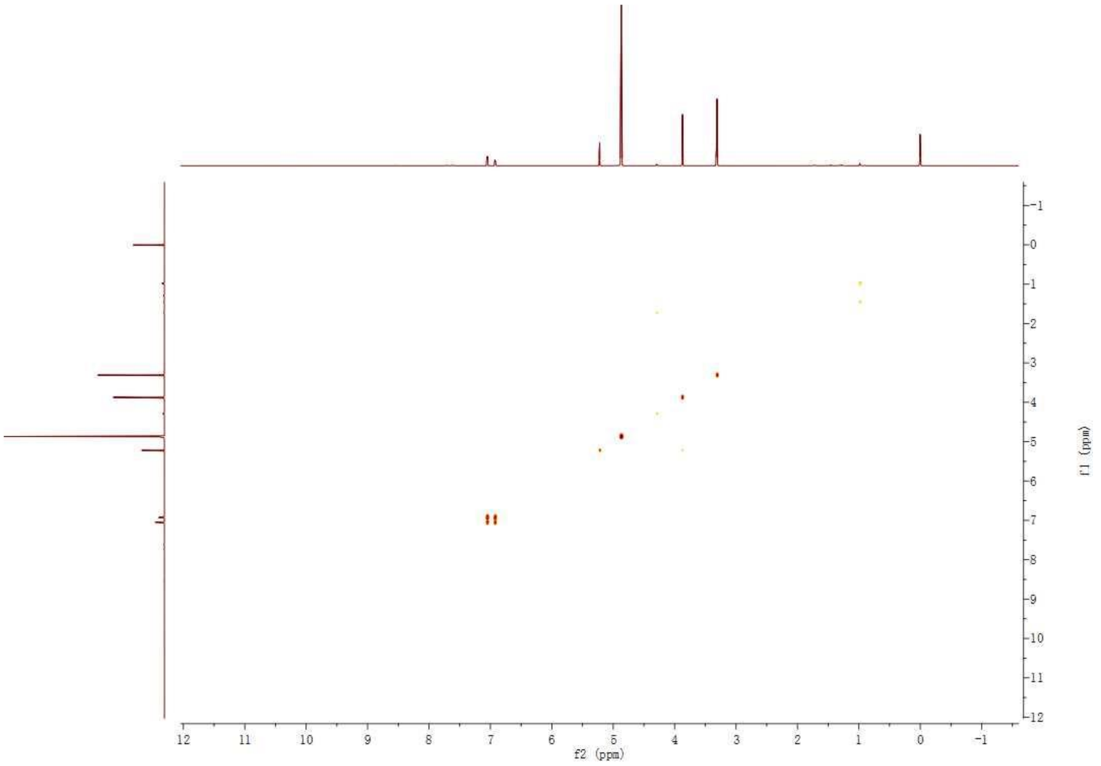

Figure S69. ^{1}H-^{1}H COSY spectra of compound **8**.

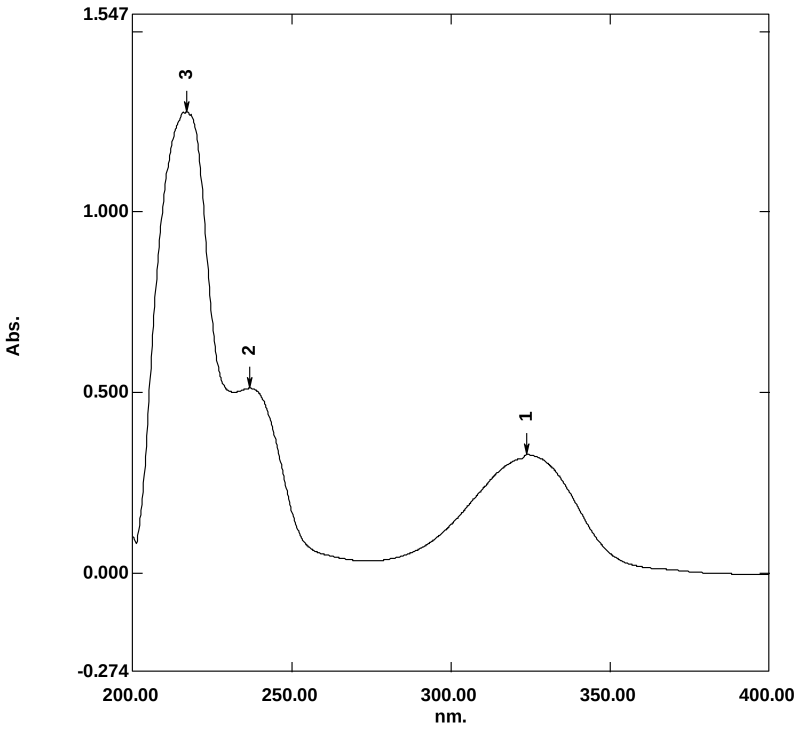

No.	波长(nm)	Abs.
1	323.60	0.328
2	236.80	0.511
3	216.80	1.278

Figure S70. UV spectra of compound **8**.

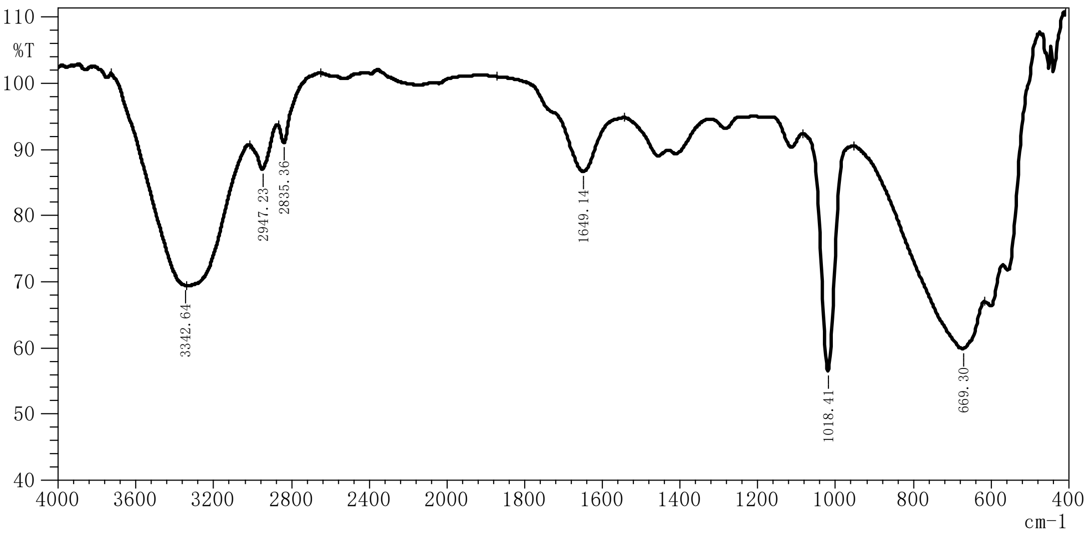

Figure S71. IR spectra of compound **8**.

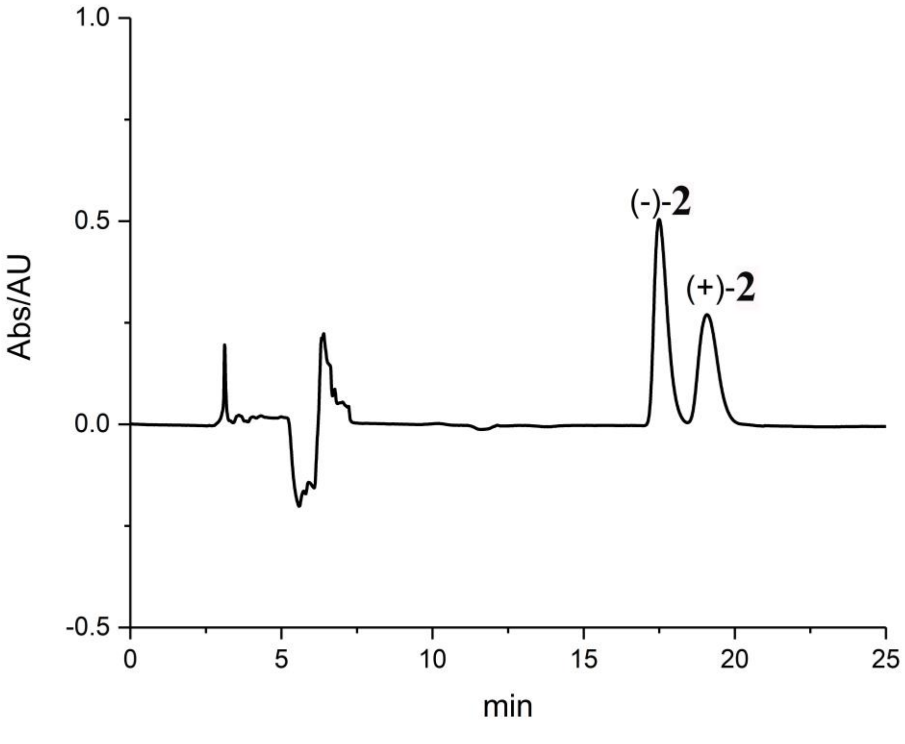

Figure S72. The chiral HPLC separation profile of compound **2**.

ECD calculation of 2.

Random conformational searches were conducted using Spartan'14 software (v1.1.4,

Wavefunction, Irvine, CA, USA) with the Molecular Merck force field (MMFF). Conformers that had the Boltzmann population greater than 5% were further optimized using Gaussian09 software (D.01, Pittsburgh, PA, USA) at the B3LYP/6-31G (d) level in the presence of methanol, utilizing the IEFPCM model to account for solvent effects. The optimized stable conformers were then selected for additional electronic circular dichroism (ECD) calculations, which were performed at the B3LYP/6-311G (d, p) level in methanol. The overall ECD data were weighted using the Boltzmann population, and SpecDis 1.70.1 software was employed to generate ECD curves and enantiomeric ECD curves. A half-bandwidth of 0.3 eV was utilized in SpecDis software (1.70.1, SpecDis, Berlin, Germany), and the contributions of each conformer, calculated using the Boltzmann distribution after UV correction, were taken into account to produce the ECD curves.

Table S1. Energies at B3LYP/6–311g (d, p) level of 3*R*-**2** and 3*S*-**2**.

Configuration	Conformer	E (Hartree)	E (kcal/mol)	Population (%)
3*R*-**2**	1	-729.512111128	-457776.144853931	22.86%
3*R*-**2**	2	-729.512945888	-457776.668674179	55.34%
3*R*-**2**	3	-729.512065627	-457776.116301599	21.79%
3*R*-**2**	4	-729.503679858	-457770.854147694	0.00%
3*R*-**2**	5	-729.504825534	-457771.573070840	0.01%
3*S*-**2**	1	-729.512109438	-457776.143793439	22.82%
3*S*-**2**	2	-729.512945925	-457776.668697397	55.35%
3*S*-**2**	3	-729.504696194	-457771.491908697	0.01%
3*S*-**2**	4	-729.512065627	-457776.116301599	21.79%
3*S*-**2**	5	-729.505957028	-457772.283094640	0.03%

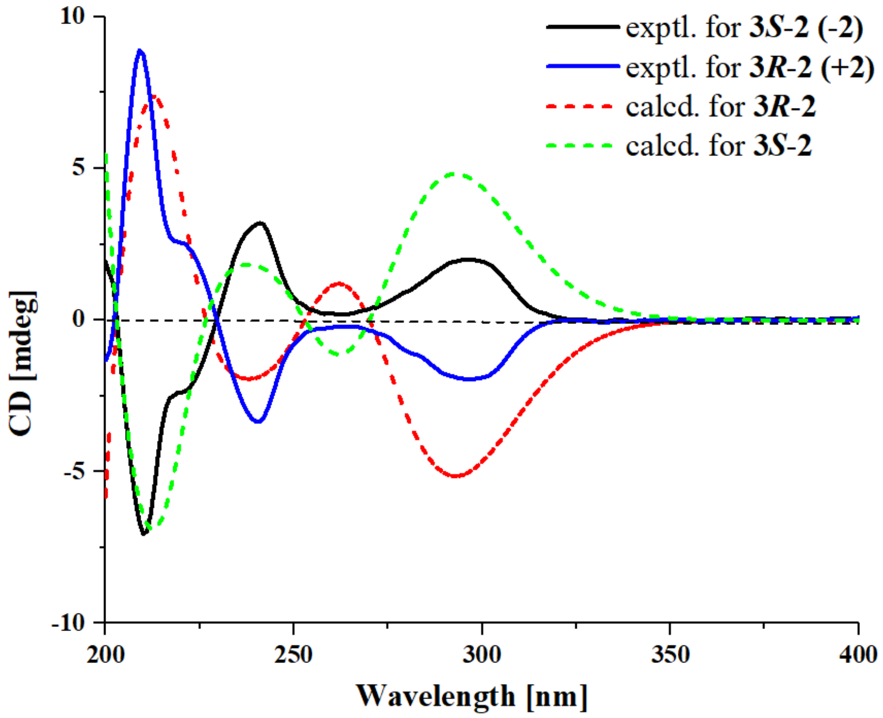

Figure S73. Comparison between the experimental ECD and the calculated ECD spectra for compound (+)-**2** and (−)-**2**.

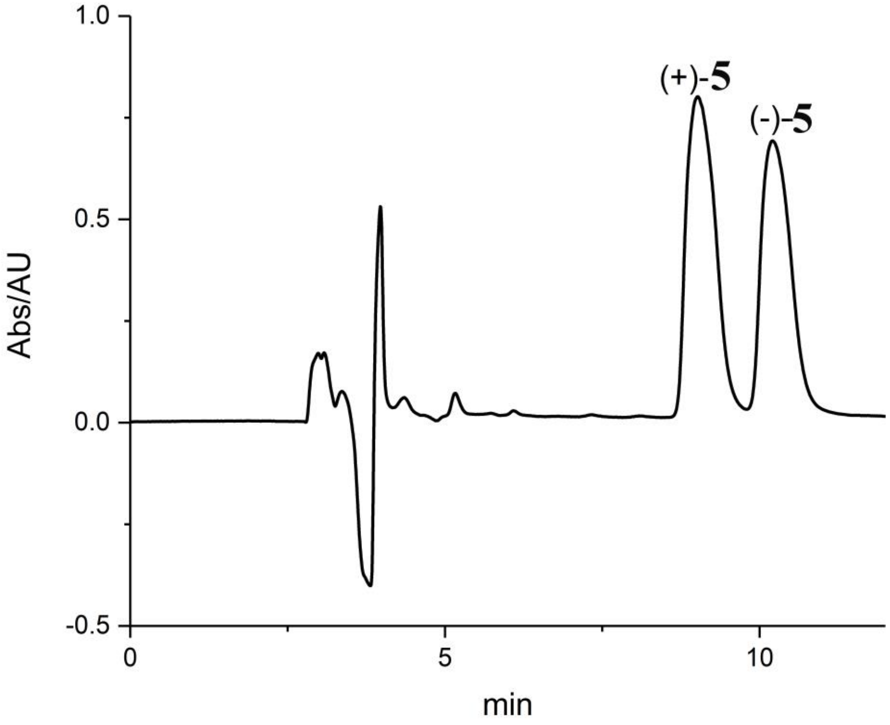

Figure S74. The chiral HPLC separation profile of compound **5**.

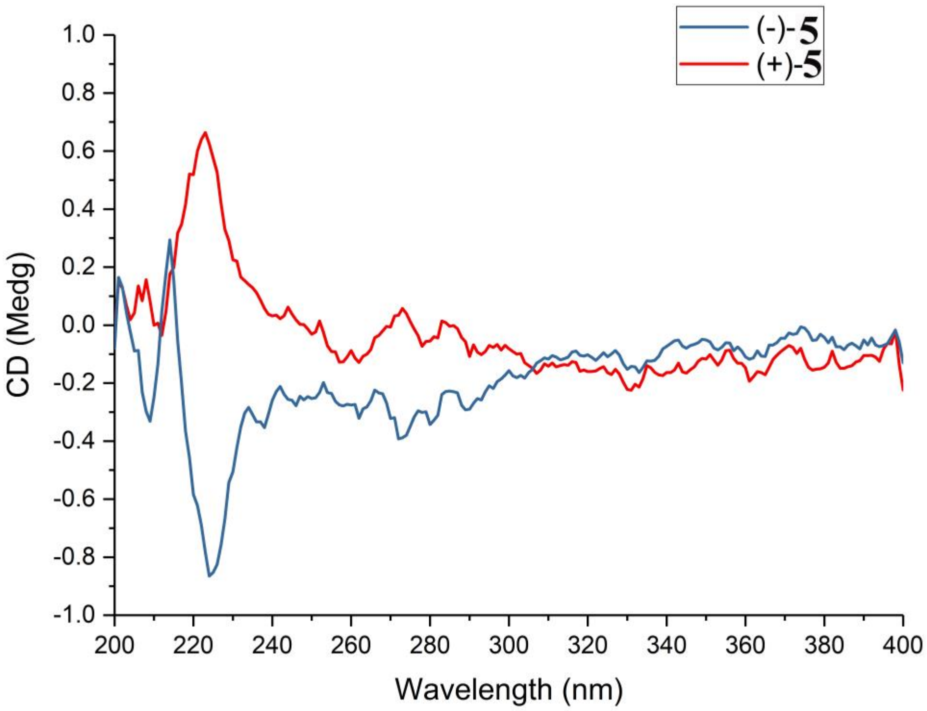

Figure S75. The experiment CD of compounds (+)-**5 and** (−)-**5**.

Computational methods of 3.

The conformational search was implemented in xtb software package using molecular dynamic simulations with the method of 100 ps/400 K/GNF0.[1] The obtained 2000 conformers were sequentially optimized on with semi-empirical level of GNF0-xTB and GNF2-xTB, and the conformers were sorted by Molclus 1.9.9.9 program.[2] The conformers within an energy window of 5 kcal/mol were subjected to further re-optimization and frequency calculations using the DFT method at B3LYP-D3(BJ)/6-31G* (IEFPCM, MeOH) level of theory. The conformers with population above 1% were selected for NMR calculation. The magnetic shielding tensors of the lower-energy conformers were computed at mPW1PW91/6-31+G** (IEFPCM, MeOH) level of theory using GIAO method, while the magnetic shielding tensors of the standard substance TMS were also calculated under the same theoretical level. Additionally, the lower-energy conformers for **3b** were further selected for TDDFT calculation at CAM-B3LYP/def2TZVP (IEFPCM, MeOH) level of theory with 12 excited states per molecule respectively. The calculated ECD curves were generated by using SpecDis[3] with a σ of 0.25 eV. The contribution of each conformer was weighted using Boltzmann averaging. All the DFT/TDDFT calculations were performed using Gaussian 16 program.

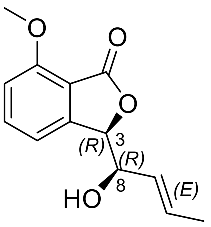

3a

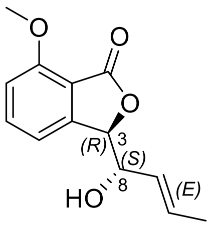

3b

Figure S76. Two computational configurations of 3*R**, 8*R** (**3a**) and 3*R**, 8*S** (**3b**).

Computational data of **3a**.

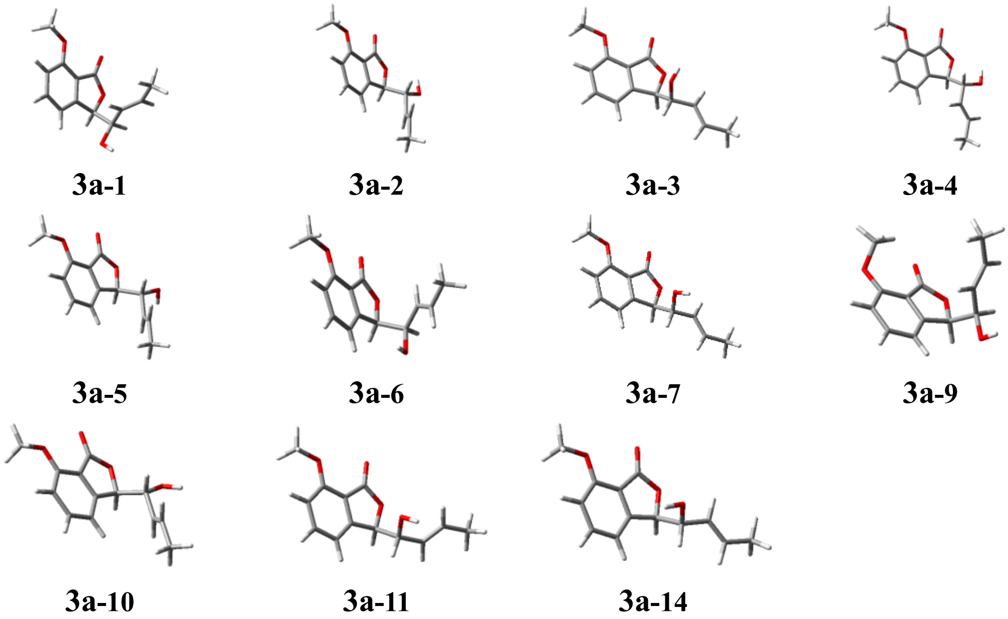

Figure S77. Optimized geometries of 11 dominant conformers of **3a** at the B3LYP-GD3BJ/6-31G(d) level of theory in methanol with the IEFPCM solvent.

Table S2. Conformational analysis of the optimized **3a** at the B3LYP/6-31G(d) of theory in methanol with the IEFPCM solvent.

Conformers	*ΔG* (kcal/mol) [a]	Population [b]
**3a-1**	0.0000	34.73%
**3a-2**	0.0628	31.23%
**3a-3**	1.0015	6.40%
**3a-4**	0.9714	6.73%
**3a-5**	1.7457	1.82%
**3a-6**	1.1370	5.08%
**3a-7**	1.2839	3.97%

**3a-9**	1.9020	1.40%
**3a-10**	1.1992	4.58%
**3a-11**	1.7978	1.66%
**3a-14**	1.5801	2.40%

[a] The relative Gibbs free energy; [b] The Boltzmann distribution of each conformer.

Table S3. Cartesian coordinates of **3a-1**.

C	2.446878	1.19207	0.937064	O	-2.84101	1.983387	-0.12671
C	1.534764	2.251945	1.013945	H	3.377263	1.257513	1.486763
C	0.316372	2.236757	0.330263	H	1.791401	3.109807	1.628605
C	0.039805	1.114378	-0.44055	H	-0.38947	3.054581	0.407751
C	0.930106	0.046858	-0.52945	H	-1.34688	1.483894	-2.07163
C	2.156869	0.0621	0.158851	H	4.690014	-1.95554	0.468072
C	-1.17548	0.772013	-1.2576	H	4.845536	-0.17733	0.390246
O	2.965046	-1.01075	0.020317	H	4.056605	-0.94861	1.801289
C	4.212106	-1.00858	0.718578	H	-3.2245	0.182484	-1.11507
C	0.345898	-0.98059	-1.40522	H	-1.69339	0.202881	1.568369
O	-0.87002	-0.50995	-1.84422	H	-3.47254	-1.81753	0.098018
C	-2.47925	0.637042	-0.44645	H	-3.66319	-2.57129	2.54698
C	-2.31513	-0.21477	0.777046	H	-2.22373	-3.27074	1.815014
C	-2.85926	-1.42687	0.911538	H	-2.06962	-1.85172	2.873266
O	0.75929	-2.06411	-1.75555	H	-3.56001	1.939028	0.523729
C	-2.691	-2.32003	2.103289				

Table S4. Cartesian coordinates of **3a-2**.

C	-2.08063	-1.9543	-0.16304	O	2.72987	2.301846	0.268611
C	-0.82048	-2.41171	-0.56479	H	-2.87698	-2.67122	-0.00854
C	0.253096	-1.54408	-0.78261	H	-0.68057	-3.47881	-0.71
C	0.022861	-0.18783	-0.58439	H	1.222382	-1.91681	-1.08892
C	-1.22727	0.289701	-0.19164	H	1.3537	1.114771	-1.71808
C	-2.30728	-0.58414	0.0311	H	-4.371	-1.62621	1.443433
C	0.952802	0.986889	-0.70755	H	-5.4176	-0.2722	0.930193
O	-3.48187	-0.0404	0.412657	H	-4.84997	-1.46283	-0.27508
C	-4.58881	-0.91669	0.637821	H	1.683527	0.822346	1.304908
C	-1.16681	1.754203	-0.0881	H	3.70067	0.146497	-0.94079
O	0.112632	2.139071	-0.43212	H	2.830238	-1.21721	1.665679
C	2.113685	1.016447	0.310698	H	3.902278	-3.132	0.32108
C	3.150901	-0.01424	-0.01307	H	4.928935	-1.94398	-0.51096
C	3.39736	-1.08631	0.743172	H	5.134285	-2.26057	1.227009

O	-2.00327	2.574491	0.216255	H	2.007535	2.949491	0.343236
C	4.396189	-2.15651	0.42133				

Table S5. Cartesian coordinates of **3a-3**.

C	2.976397	1.468943	-0.00042	O	-1.52605	0.080069	1.741599
C	1.997384	2.452207	-0.18758	H	4.002001	1.772787	0.166574
C	0.655132	2.132763	-0.40931	H	2.300444	3.494663	-0.15907
C	0.322177	0.784852	-0.43638	H	-0.08816	2.90958	-0.55397
C	1.278876	-0.20954	-0.25326	H	-1.4432	0.336968	-1.61793
C	2.630583	0.110109	-0.03045	H	5.375313	-1.56268	0.467141
C	-1.01377	0.123256	-0.63379	H	5.294515	-0.05284	-0.48492
O	3.494339	-0.91326	0.136412	H	4.996074	-0.01793	1.281112
C	4.871152	-0.6021	0.36299	H	-2.17622	1.529416	0.489542
C	0.618236	-1.51944	-0.33361	H	-3.35841	-1.30534	0.115873
O	-0.72067	-1.29583	-0.57974	H	-4.5036	1.539539	0.089068
C	-2.05074	0.442039	0.463174	H	-6.26121	0.184981	-1.21365
C	-3.37323	-0.2169	0.184839	H	-5.77773	-1.26885	-0.31347
C	-4.51728	0.450512	0.019498	H	-6.57941	0.084658	0.514386
O	1.051775	-2.6448	-0.22657	H	-1.44794	-0.88875	1.747412
C	-5.84954	-0.17765	-0.26264				

Table S6. Cartesian coordinates of **3a-4**.

C	-2.36142	-1.90864	0.004026	O	2.790936	1.963972	0.366555
C	-1.12026	-2.50662	-0.24146	H	-3.22881	-2.53811	0.156727
C	0.04052	-1.75627	-0.4476	H	-1.06605	-3.5907	-0.27373
C	-0.08042	-0.37241	-0.39904	H	0.98997	-2.23963	-0.64534
C	-1.31028	0.243086	-0.16926	H	1.465515	0.675299	-1.52668
C	-2.47857	-0.51163	0.041648	H	-4.73561	-1.22768	1.355445
C	0.956531	0.704981	-0.55853	H	-5.61019	0.146711	0.621454
O	-3.6257	0.163657	0.260007	H	-5.05663	-1.20572	-0.40689
C	-4.82243	-0.59067	0.468667	H	1.47951	0.828737	1.514343
C	-1.13171	1.700677	-0.22552	H	2.489251	-1.32513	0.973242
O	0.197581	1.941819	-0.50279	H	4.569335	0.484251	-0.35574
C	2.024109	0.784962	0.556784	H	5.357893	-1.88564	-0.99416
C	2.91992	-0.42146	0.54682	H	5.976373	-1.45896	0.596593
C	4.159951	-0.43742	0.053339	H	4.557586	-2.52035	0.460092
O	-1.91662	2.61176	-0.08868	H	2.151033	2.689737	0.271925
C	5.052588	-1.64234	0.032097				

Table S7. Cartesian coordinates of **3a-5**.

C	-2.07381	-1.93595	-0.21689	O	2.79046	2.295818	0.267598
C	-0.81637	-2.37272	-0.64936	H	-2.86481	-2.66175	-0.07701
C	0.250089	-1.49273	-0.84943	H	-0.67289	-3.43343	-0.83317
C	0.017111	-0.14451	-0.60136	H	1.216365	-1.85242	-1.1794
C	-1.23114	0.311996	-0.18099	H	1.302599	1.20125	-1.72368
C	-2.304	-0.57428	0.02488	H	-4.34168	-1.67356	1.429619
C	0.936485	1.04608	-0.70165	H	-5.40412	-0.31111	0.974546
O	-3.47764	-0.04961	0.437511	H	-4.84618	-1.45913	-0.2757
C	-4.57521	-0.94004	0.650161	H	1.733033	0.911508	1.285253
C	-1.1754	1.774747	-0.03933	H	3.557642	-0.0634	-1.02097
O	0.094316	2.177162	-0.37403	H	2.872669	-1.11296	1.775763
C	2.134743	1.026317	0.273562	H	3.770079	-3.19081	0.552638
C	3.083492	-0.09589	-0.03764	H	4.75585	-2.1321	-0.47948
C	3.36325	-1.09571	0.80162	H	5.100543	-2.28009	1.258868
O	-2.02006	2.578856	0.291378	H	3.274155	2.374099	-0.57179
C	4.29912	-2.22964	0.510565				

Table S8. Cartesian coordinates of **3a-6**.

C	-2.18164	-1.65297	-0.37307	O	3.042882	-0.61973	-1.34835
C	-1.16822	-2.21964	-1.15336	H	-3.03267	-2.2607	-0.09317
C	-0.04236	-1.49337	-1.55293	H	-1.2695	-3.25669	-1.45901
C	0.039238	-0.17008	-1.13523	H	0.715594	-1.94398	-2.18373
C	-0.96545	0.417759	-0.36941	H	1.307569	1.058778	-2.40246
C	-2.1	-0.31126	0.029919	H	-4.79071	0.311991	1.746964
C	1.121205	0.852405	-1.34455	H	-4.75776	-0.7426	0.304833
O	-3.02648	0.331788	0.769152	H	-3.92049	-1.24732	1.806175
C	-4.19	-0.39426	1.174305	H	3.148941	1.33366	-0.86823
C	-0.61111	1.82434	-0.12769	H	1.76661	1.061016	1.338076
O	0.602031	2.057629	-0.73957	H	3.433774	-1.48237	0.968971
C	2.473713	0.491682	-0.67151	H	2.193798	-1.89597	3.187134
C	2.330846	0.291625	0.814418	H	3.664532	-0.97299	3.474367
C	2.844879	-0.73305	1.4975	H	2.09542	-0.13914	3.43232
O	-1.18945	2.705542	0.468212	H	2.581741	-1.41381	-1.02878
C	2.687442	-0.93846	2.975085				

Table S9. Cartesian coordinates of **3a-7**.

C	3.003501	1.414724	0.004183	O	-1.42239	0.241616	1.637785
C	2.058832	2.422326	-0.22655	H	4.033153	1.69204	0.191543
C	0.71391	2.137252	-0.47602	H	2.391801	3.455927	-0.21018
C	0.343802	0.798768	-0.48792	H	-0.00407	2.9314	-0.65153
C	1.265395	-0.21925	-0.26385	H	-1.43077	0.398009	-1.6802

C	2.618869	0.06597	-0.00971	H	4.941577	-0.10125	1.369837
C	-1.00691	0.167827	-0.69785	H	5.299485	-1.67339	0.600101
O	3.448497	-0.97849	0.200072	H	5.29017	-0.18292	-0.38519
C	4.826181	-0.70156	0.460832	H	-2.21865	1.617733	0.280984
C	0.566562	-1.51078	-0.34768	H	-3.25985	-1.29328	0.255972
O	-0.74774	-1.2545	-0.65509	H	-4.5399	1.48454	-0.00502
C	-2.03627	0.536061	0.380673	H	-5.70374	-1.39847	-0.07917
C	-3.32952	-0.2073	0.211072	H	-6.53305	-0.00085	0.642358
C	-4.50837	0.394145	0.030959	H	-6.28134	-0.06206	-1.0983
O	0.966963	-2.64601	-0.20921	H	-2.11756	0.311344	2.311293
C	-5.82097	-0.31147	-0.13329				

Table S10. Cartesian coordinates of **3a-9**.

C	2.444755	1.407626	1.034184	O	-2.87128	1.875042	0.036686
C	1.493742	2.42744	1.138175	H	3.380636	1.463645	1.580013
C	0.277286	2.36066	0.451682	H	1.704595	3.281502	1.774781
C	0.059951	1.251948	-0.35441	H	-0.47034	3.139138	0.545583
C	1.000901	0.225792	-0.47442	H	-1.35839	1.656199	-1.95313
C	2.214447	0.284775	0.233011	H	2.092409	-2.42279	-0.06988
C	-1.12962	0.896027	-1.20003	H	3.765091	-2.56419	0.541665
O	3.197618	-0.65674	0.155177	H	2.478105	-1.9555	1.621487
C	2.843058	-1.98267	0.58856	H	-3.12728	0.141179	-1.10187
C	0.49188	-0.74465	-1.46184	H	-1.60699	0.067252	1.585069
O	-0.73382	-0.30792	-1.88979	H	-3.09623	-2.00898	-0.11241
C	-2.41319	0.593443	-0.39867	H	-1.67536	-3.44874	1.477931
C	-2.16438	-0.34542	0.744675	H	-1.72262	-2.13637	2.674675
C	-2.54637	-1.62499	0.748144	H	-3.20188	-3.026	2.244914
O	0.982615	-1.75896	-1.91189	H	-3.58184	1.722411	0.67989
C	-2.26928	-2.60333	1.849236				

Table S11. Cartesian coordinates of **3a-10**.

C	-2.08427	-1.93376	-0.21822	O	2.785269	2.282757	0.079002
C	-0.82673	-2.37161	-0.64909	H	-2.87701	-2.65845	-0.08248
C	0.241496	-1.49275	-0.845	H	-0.68476	-3.43201	-0.83574
C	0.01049	-0.14521	-0.59247	H	1.207581	-1.853	-1.1749
C	-1.23716	0.312242	-0.17244	H	1.286859	1.206936	-1.72326
C	-2.31235	-0.57249	0.027657	H	-5.41606	-0.30517	0.964099
C	0.929194	1.04543	-0.70047	H	-4.85441	-1.44911	-0.28821
O	-3.48591	-0.04683	0.438966	H	-4.36015	-1.67274	1.418945
C	-4.58717	-0.93505	0.641524	H	1.749354	0.914455	1.273854
C	-1.17844	1.774215	-0.02251	H	3.521535	-0.09349	-1.05773

O	0.09088	2.176364	-0.35835	H	2.923217	-1.06197	1.79109
C	2.135212	1.018961	0.248791	H	5.146157	-2.22939	1.254942
C	3.073889	-0.10992	-0.06327	H	3.804523	-3.17623	0.621484
C	3.385893	-1.07739	0.803145	H	4.75127	-2.14951	-0.47745
O	-2.02078	2.577616	0.31538	H	3.603435	2.244218	0.600469
C	4.323746	-2.21332	0.528169				

Table S12. Cartesian coordinates of **3a-11**.

C	3.127511	1.144171	0.162889	O	-1.54878	0.578358	1.462317
C	2.361469	2.29371	-0.06557	H	4.172279	1.251703	0.425434
C	1.008518	2.229042	-0.40889	H	2.843104	3.262567	0.028355
C	0.44434	0.964672	-0.51733	H	0.431069	3.131085	-0.58173
C	1.186654	-0.19148	-0.29795	H	-1.28348	0.893274	-1.83279
C	2.54764	-0.128	0.050277	H	5.171436	-0.76018	-0.17991
C	-0.96878	0.557466	-0.83941	H	4.722035	-0.71278	1.553395
O	3.197007	-1.29502	0.248628	H	4.893566	-2.28132	0.715178
C	4.580001	-1.24328	0.605531	H	-1.96875	2.139798	0.15315
C	0.308035	-1.35512	-0.4948	H	-3.85153	1.090803	-1.00065
O	-0.93144	-0.88651	-0.85553	H	-3.56979	-0.87395	1.332419
C	-2.00004	1.037906	0.196885	H	-6.12487	-0.69948	1.016267
C	-3.39414	0.581691	-0.15197	H	-5.85962	-0.28714	-0.69161
C	-4.05936	-0.37565	0.496609	H	-5.45848	-1.90554	-0.07794
O	0.524965	-2.54385	-0.40147	H	-2.21647	0.842497	2.115528
C	-5.4476	-0.83432	0.162594				

Table S13. Cartesian coordinates of **3a-14**.

C	2.995423	1.391881	-0.0134	O	-1.4548	0.280247	1.71018
C	2.063126	2.409313	-0.24574	H	4.027707	1.658109	0.175321
C	0.714558	2.138779	-0.49722	H	2.407023	3.43916	-0.22968
C	0.330604	0.803268	-0.51255	H	0.006217	2.940621	-0.67662
C	1.240352	-0.22466	-0.2775	H	-1.48105	0.423119	-1.66339
C	2.596695	0.046339	-0.02387	H	5.255773	-1.72074	0.59679
C	-1.03114	0.188092	-0.69402	H	5.265923	-0.23366	-0.39361
O	3.413832	-1.00488	0.191597	H	4.9157	-0.14191	1.360991
C	4.795039	-0.74355	0.453714	H	-2.21381	1.649057	0.323257
C	0.52439	-1.50883	-0.35153	H	-3.2359	-1.26148	0.475257
O	-0.78752	-1.23599	-0.6521	H	-4.51303	1.458987	-0.14698
C	-2.01954	0.571532	0.432754	H	-6.17901	-0.22003	-1.16451
C	-3.30781	-0.18695	0.315137	H	-5.65187	-1.42972	0.025332
C	-4.47624	0.379348	0.007493	H	-6.53096	0.026557	0.54221
O	0.91239	-2.64737	-0.20887	H	-0.658	0.827793	1.810524

C	-5.77311	-0.35655	-0.15351				

Computational data of **3b**.

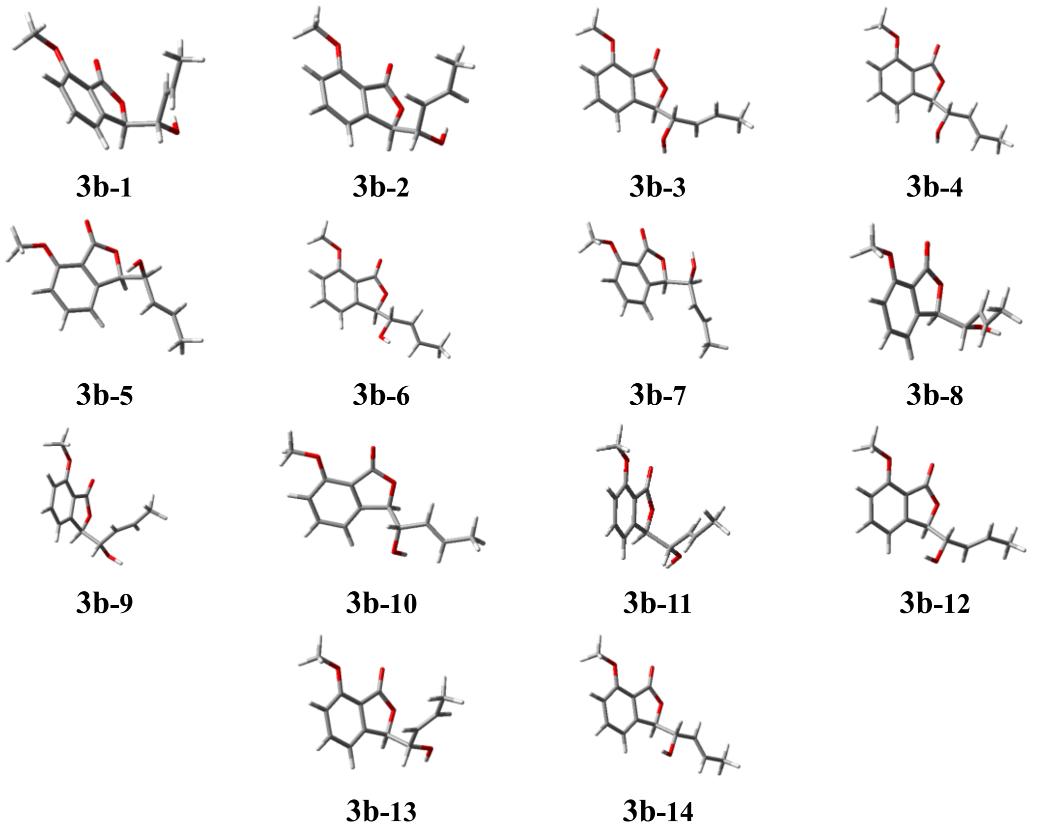

Figure S78. Optimized geometries of 14 dominant conformers of **3b** at the B3LYP-GD3BJ/6-31G(d) level of theory in methanol with the IEFPCM solvent.

Table S14. Conformational analysis of the optimized **3b** at the B3LYP/6-31G(d) of theory in methanol with the IEFPCM solvent.

Conformers	ΔG (kcal/mol) [a]	Population [b]
**3b-1**	0.0000	26.08%
**3b-2**	0.1205	21.27%
**3b-3**	1.3473	2.68%
**3b-4**	1.3824	2.52%
**3b-5**	1.2876	2.96%
**3b-6**	1.3611	2.62%
**3b-7**	0.9174	5.53%
**3b-8**	0.1719	19.50%
**3b-9**	0.8791	5.90%
**3b-10**	1.3818	2.53%
**3b-11**	1.1188	3.94%

**3b-12**	1.8060	1.23%
**3b-13**	1.8016	1.24%
**3b-14**	1.5192	2.00%

[a] The relative Gibbs free energy; [b] The Boltzmann distribution of each conformer.

Table S15. Cartesian coordinates of **3b-1**.

C	2.373662	1.19234	-0.92339	O	-3.61333	-1.17307	-0.42219
C	1.411083	1.615006	-1.84787	H	3.311057	1.729804	-0.85724
C	0.17971	0.96935	-1.98675	H	1.637301	2.473931	-2.47269
C	-0.05692	-0.12328	-1.16309	H	-0.55509	1.313513	-2.70658
C	0.886346	-0.56344	-0.23759	H	-1.52263	-1.5324	-1.94697
C	2.125867	0.087115	-0.09618	H	4.112912	1.300281	1.28466
C	-1.27832	-0.98572	-1.03075	H	4.765445	-0.29195	1.766018
O	2.984407	-0.39745	0.824943	H	4.828052	0.213324	0.053276
C	4.24621	0.256537	0.980355	H	-2.83177	0.452272	-1.34105
C	0.339821	-1.72534	0.479117	H	-2.08037	-0.08123	1.612638
O	-0.92012	-1.96872	-0.02651	H	-2.5327	2.432734	-0.08111
C	-2.53805	-0.2403	-0.54559	H	-1.19124	3.301012	1.935743
C	-2.29669	0.520226	0.729181	H	-2.90982	3.281078	2.315959
C	-2.3193	1.851561	0.817397	H	-1.85787	1.981316	2.920699
O	0.80506	-2.42095	1.354024	H	-3.32523	-1.84095	0.22342
C	-2.0561	2.637383	2.067001				

Table S16. Cartesian coordinates of **3b-2**.

C	2.688899	0.911299	0.984707	O	-3.48503	0.843046	-0.88393
C	1.914031	2.041392	1.270288	H	3.653726	0.800533	1.462995
C	0.659791	2.249525	0.689394	H	2.308106	2.775421	1.966787
C	0.205273	1.280206	-0.19541	H	0.073083	3.131144	0.924223
C	0.959954	0.148035	-0.49603	H	-1.26409	2.011401	-1.62772
C	2.221489	-0.06246	0.089412	H	4.517099	-2.36083	-0.06925
C	-1.0969	1.179993	-0.93656	H	4.873542	-0.61607	0.07683
O	2.88918	-1.18373	-0.25204	H	4.089905	-1.48905	1.430871
C	4.16991	-1.41283	0.341113	H	-2.50264	1.977838	0.463886
C	0.21115	-0.67904	-1.45448	H	-1.32669	0.033568	1.643849
O	-0.96791	-0.02247	-1.73342	H	-3.83597	-1.22376	0.423291
C	-2.35166	1.018127	-0.04708	H	-2.68308	-3.20899	1.597216
C	-2.17863	-0.07907	0.975005	H	-1.92832	-2.06906	2.731433
C	-2.98038	-1.1384	1.091836	H	-3.68664	-2.32786	2.743052
O	0.475821	-1.7373	-1.97912	H	-3.29865	0.059469	-1.43011
C	-2.80392	-2.23847	2.096258				

Table S17. Cartesian coordinates of **3b-3**.

C	3.106469	1.157081	0.29238	O	-2.13914	2.257662	0.229184
C	2.28529	2.276578	0.110265	H	4.143312	1.303097	0.567596
C	0.93938	2.163696	-0.24698	H	2.717942	3.262417	0.253823
C	0.435451	0.879616	-0.41747	H	0.310641	3.035893	-0.37265
C	1.23478	-0.24848	-0.24077	H	-1.24235	0.74735	-1.78612
C	2.589769	-0.13483	0.11963	H	5.241375	-0.62948	-0.12358
C	-0.94868	0.415329	-0.78293	H	4.785304	-0.68078	1.607802
O	3.293899	-1.27724	0.27085	H	5.034625	-2.2005	0.701893
C	4.671641	-1.17487	0.636703	H	-1.70724	0.549819	1.226933
C	0.420545	-1.44697	-0.48991	H	-3.77151	0.357771	-1.07208
O	-0.84771	-1.0266	-0.81734	H	-3.59108	-0.77575	1.770525
C	-2.04502	0.827993	0.2239	H	-5.71358	-1.02482	-0.48921
C	-3.36044	0.172677	-0.07711	H	-5.26224	-2.34855	0.607223
C	-4.02632	-0.59734	0.786124	H	-6.10066	-0.9382	1.24388
O	0.696865	-2.62609	-0.44999	H	-2.6253	2.523033	-0.56955
C	-5.34462	-1.25769	0.514806				

Table S18. Cartesian coordinates of **3b-4**.

C	3.047606	1.378141	0.080829	O	-2.2849	1.847983	0.342392
C	2.087323	2.372887	-0.14076	H	4.074263	1.668681	0.264753
C	0.745097	2.072439	-0.38638	H	2.405762	3.411023	-0.11898
C	0.390252	0.728894	-0.40274	H	0.010443	2.851532	-0.5454
C	1.329689	-0.2772	-0.1839	H	-1.34759	0.272478	-1.62343
C	2.681052	0.024931	0.063783	H	5.387826	-1.68301	0.645319
C	-0.94787	0.075996	-0.6216	H	5.350941	-0.18768	-0.33195
O	3.524747	-1.00979	0.264796	H	5.01651	-0.1196	1.42647
C	4.90104	-0.71659	0.515303	H	-1.56709	0.304114	1.417403
C	0.654418	-1.57968	-0.26098	H	-3.06529	-1.46937	0.180698
O	-0.67462	-1.34163	-0.53468	H	-4.66436	1.135283	0.422186
C	-2.01999	0.454542	0.429008	H	-5.48242	-1.83912	0.026507
C	-3.25201	-0.40301	0.290155	H	-6.3903	-0.67363	1.014174
C	-4.50172	0.065725	0.292062	H	-6.31928	-0.4675	-0.73198
O	1.071893	-2.70921	-0.13213	H	-2.80282	1.992337	-0.46839
C	-5.73216	-0.77947	0.141806				

Table S19. Cartesian coordinates of **3b-5**.

C	2.183044	1.939863	-0.00386	O	-1.37123	-1.10078	1.64977
C	0.929105	2.493168	-0.2851	H	3.015927	2.600422	0.201078
C	-0.18924	1.700529	-0.55924	H	0.830494	3.574543	-0.2919
C	-0.00926	0.321739	-0.54382	H	-1.14937	2.149506	-0.78548
C	1.233647	-0.24862	-0.27568	H	-1.51811	-0.74626	-1.70487
C	2.358716	0.547858	0.00237	H	4.92181	1.366216	-0.31156
C	-0.99626	-0.79816	-0.74513	H	4.52297	1.320913	1.43437
O	3.525107	-0.08322	0.251125	H	5.489104	0.008482	0.701981
C	4.676644	0.715325	0.534641	H	-2.55745	-1.88365	0.222414
C	1.11148	-1.71199	-0.38521	H	-2.6464	1.150645	0.784178
O	-0.18251	-1.99346	-0.74461	H	-4.63786	-0.86664	-0.3797
C	-2.03402	-0.93903	0.397387	H	-5.63315	1.409753	-1.05125
C	-3.01888	0.196819	0.409112	H	-6.15874	0.971844	0.570273
C	-4.28157	0.097367	-0.01302	H	-4.84175	2.147633	0.35831
O	1.929004	-2.59377	-0.23661	H	-0.97307	-0.24622	1.885963
C	-5.27465	1.220569	-0.031				

Table S20. Cartesian coordinates of **3b-6**.

C	3.053434	1.362602	0.016793	O	-2.2683	1.86326	0.317214
C	2.095995	2.365713	-0.17885	H	4.089079	1.643838	0.160317
C	0.742523	2.077483	-0.37123	H	2.426085	3.400464	-0.17898
C	0.374299	0.737548	-0.3618	H	0.009161	2.861648	-0.5103
C	1.310154	-0.27676	-0.16928	H	-1.41945	0.295137	-1.50899
C	2.6727	0.013229	0.026368	H	5.325089	-0.2312	-0.46103
C	-0.97785	0.096412	-0.52667	H	5.052708	-0.14109	1.307023
O	3.512774	-1.02825	0.207663	H	5.381186	-1.71645	0.530617
C	4.899711	-0.74653	0.407002	H	-1.52149	0.342596	1.530075
C	0.617169	-1.57208	-0.21128	H	-3.11263	-1.4037	0.827963
O	-0.71832	-1.32142	-0.43449	H	-4.51675	1.064195	-0.32323
C	-2.00794	0.484821	0.552038	H	-6.47367	-0.37398	0.547861
C	-3.24114	-0.38009	0.480906	H	-6.03123	-0.87458	-1.07986
C	-4.42458	0.036104	0.025092	H	-5.47722	-1.82658	0.314386
O	1.027095	-2.70554	-0.09187	H	-2.87751	2.164411	1.010486
C	-5.66104	-0.80996	-0.04817				

Table S21. Cartesian coordinates of **3b-7**.

C	2.105708	1.980587	0.044103	O	-1.5084	-1.31694	1.612719
C	0.824644	2.492253	-0.19294	H	2.91859	2.666866	0.244911
C	-0.27015	1.665895	-0.4591	H	0.685946	3.569085	-0.17013

C	-0.04265	0.294515	-0.47562	H	-1.25193	2.083121	-0.6485
C	1.228207	-0.23378	-0.25802	H	-1.45666	-0.79477	-1.7113
C	2.331338	0.597104	0.010989	H	4.853185	1.51591	-0.3495
C	-0.99087	-0.84846	-0.72245	H	4.504021	1.41337	1.404486
O	3.526035	0.00267	0.212857	H	5.496984	0.155756	0.613935
C	4.655062	0.835878	0.486044	H	-2.67813	-1.93375	0.003839
C	1.161775	-1.69523	-0.3902	H	-2.59793	0.949863	1.066304
O	-0.14246	-2.02549	-0.69525	H	-4.55775	-0.60586	-0.7094
C	-2.09557	-1.06429	0.341093	H	-5.2679	1.846384	-1.04959
C	-2.9929	0.130455	0.467998	H	-6.05573	1.176925	0.37468
C	-4.18567	0.235059	-0.12191	H	-4.63524	2.22941	0.566032
O	2.011614	-2.55124	-0.28516	H	-0.96265	-2.11461	1.513185
C	-5.07713	1.438401	-0.04836				

Table S22. Cartesian coordinates of **3b-8**.

C	-2.40496	-1.16095	-0.98088	O	3.574877	1.177874	-0.60395
C	-1.44595	-1.53226	-1.93053	H	-3.34219	-1.70127	-0.94066
C	-0.215	-0.8795	-2.03819	H	-1.67438	-2.35539	-2.60107
C	0.024995	0.166313	-1.15639	H	0.515936	-1.18289	-2.77995
C	-0.91453	0.554094	-0.20525	H	1.464936	1.633712	-1.87397
C	-2.15336	-0.10289	-0.09506	H	-4.13616	-1.38996	1.223979
C	1.243781	1.030747	-0.98698	H	-4.78641	0.173458	1.794462
O	-3.00832	0.330325	0.855039	H	-4.85468	-0.23657	0.056726
C	-4.26974	-0.3309	0.97811	H	2.646368	-0.50904	-1.40945
C	-0.36273	1.676141	0.570141	H	2.264084	0.173854	1.5817
O	0.892415	1.946497	0.077022	H	2.457632	-2.4133	-0.05591
C	2.500857	0.234525	-0.61041	H	3.059374	-3.19922	2.314764
C	2.364345	-0.46954	0.708709	H	2.113693	-1.84911	2.982647
C	2.355949	-1.79833	0.839783	H	1.310847	-3.17624	2.115952
O	-0.82837	2.322292	1.483135	H	4.356866	0.709696	-0.2697
C	2.202882	-2.53639	2.135367				

Table S23. Cartesian coordinates of **3b-9**.

C	-2.6986	-1.00223	0.860296	O	3.505002	-0.93366	-0.75542
C	-1.93086	-2.15025	1.08793	H	-3.6804	-0.92867	1.31049
C	-0.6559	-2.31243	0.538763	H	-2.34745	-2.93529	1.711993
C	-0.1739	-1.27878	-0.25407	H	-0.07532	-3.20921	0.726857
C	-0.92019	-0.12824	-0.49557	H	1.354799	-1.8991	-1.68173
C	-2.20238	0.036596	0.058477	H	-4.12802	1.351803	1.433949

C	1.155391	-1.11936	-0.93995	H	-4.50205	2.335983	-0.00966
O	-2.86208	1.18062	-0.21998	H	-4.85428	0.584325	-0.01265
C	-4.16539	1.359921	0.339218	H	2.337546	-2.03269	0.56963
C	-0.13784	0.763221	-1.36786	H	1.508458	-0.18179	1.895498
O	1.049185	0.132368	-1.64984	H	3.399693	1.434918	0.110801
C	2.337587	-1.06256	0.04578	H	3.549921	2.653689	2.380774
C	2.171013	0.041186	1.060579	H	2.170105	3.246567	1.463621
C	2.752643	1.238712	0.964929	H	1.916697	2.071443	2.771935
O	-0.39185	1.854946	-1.82798	H	4.252088	-0.80939	-0.14773
C	2.584457	2.354733	1.951802				

Table S24. Cartesian coordinates of **3b-10**.

C	3.047538	1.377939	0.081066	O	-2.28454	1.847981	0.343007
C	2.087474	2.3728	-0.14087	H	4.074172	1.668323	0.265306
C	0.745269	2.072535	-0.38688	H	2.406044	3.410894	-0.11908
C	0.390245	0.729058	-0.40328	H	0.010864	2.851794	-0.54621
C	1.329449	-0.27716	-0.18411	H	-1.34808	0.273173	-1.62377
C	2.68075	0.024782	0.063948	H	5.387198	-1.68341	0.645963
C	-0.94796	0.076354	-0.62219	H	5.350552	-0.18805	-0.33129
O	3.524253	-1.01008	0.265268	H	5.015962	-0.11996	1.427095
C	4.900546	-0.71693	0.515913	H	-1.56595	0.303846	1.417174
C	0.654009	-1.57957	-0.26146	H	-3.06485	-1.46948	0.182204
O	-0.67486	-1.34137	-0.53582	H	-4.66375	1.135427	0.421373
C	-2.01952	0.454603	0.429127	H	-6.39	-0.67284	1.014356
C	-3.25149	-0.40303	0.29079	H	-6.31847	-0.46834	-0.73194
C	-4.50119	0.065766	0.292081	H	-5.48189	-1.83927	0.028041
O	1.071278	-2.70913	-0.13244	H	-2.80207	1.992736	-0.46797
C	-5.73158	-0.77949	0.142298				

Table S25. Cartesian coordinates of **3b-11**.

C	2.389222	1.087722	-1.01668	O	-3.72195	-0.94518	-0.43515
C	1.430815	1.500154	-1.94973	H	3.345892	1.593549	-0.98674
C	0.175972	0.892304	-2.04429	H	1.679151	2.319418	-2.61791
C	-0.08944	-0.14995	-1.16541	H	-0.55334	1.226581	-2.77427
C	0.848892	-0.57752	-0.23001	H	-1.59246	-1.54828	-1.87015
C	2.112617	0.032513	-0.13474	H	4.76297	-0.35525	1.710739
C	-1.33858	-0.96436	-0.97735	H	4.819613	0.068041	-0.02423
O	2.966468	-0.44005	0.797512	H	4.160543	1.235513	1.16399
C	4.2524	0.174701	0.906841	H	-2.70316	0.638848	-1.3381

C	0.265453	-1.68049	0.549355	H	-2.38276	-0.10448	1.638464
O	-1.00415	-1.90428	0.069032	H	-2.02394	2.47432	0.016697
C	-2.5644	-0.12108	-0.55468	H	-1.84297	1.832021	3.053629
C	-2.34315	0.549742	0.769073	H	-0.76514	2.962786	2.207958
C	-2.06373	1.846736	0.908056	H	-2.47129	3.356858	2.388265
O	0.716082	-2.34485	1.456771	H	-3.90038	-1.33671	-1.30597
C	-1.77345	2.528126	2.21161				

Table S26. Cartesian coordinates of **3b-12**.

C	3.110301	1.143788	0.176363	O	-2.04732	2.232579	0.446012
C	2.307177	2.261408	-0.07328	H	4.154259	1.289966	0.422643
C	0.952246	2.146466	-0.39885	H	2.759399	3.246953	-0.01539
C	0.422068	0.862117	-0.46339	H	0.353897	3.024032	-0.61605
C	1.208074	-0.26497	-0.231	H	-1.34383	0.728741	-1.72414
C	2.571059	-0.14946	0.096159	H	5.209121	-0.69891	-0.17209
C	-0.98173	0.399724	-0.74492	H	4.792576	-0.63423	1.569267
O	3.261807	-1.2886	0.305744	H	4.998165	-2.21148	0.75544
C	4.649907	-1.18603	0.633926	H	-1.67972	0.353039	1.288853
C	0.376942	-1.46403	-0.42188	H	-3.84659	0.82274	-0.8616
O	-0.88751	-1.04264	-0.76234	H	-3.53389	-1.11557	1.495916
C	-2.00776	0.807709	0.344124	H	-5.81322	-0.62653	-0.56291
C	-3.38457	0.331589	-0.00428	H	-5.3508	-2.22884	0.049997
C	-4.02523	-0.64207	0.644742	H	-6.07811	-1.05571	1.141925
O	0.642257	-2.64293	-0.33696	H	-1.23556	2.515363	0.897486
C	-5.38841	-1.15904	0.2941				

Table S27. Cartesian coordinates of **3b-13**.

C	-2.68016	-0.9792	0.900993	O	3.566235	-0.81106	-0.65504
C	-1.90477	-2.11404	1.165744	H	-3.65802	-0.89236	1.357308
C	-0.63388	-2.29175	0.611756	H	-2.31176	-2.87599	1.823837
C	-0.16384	-1.28736	-0.22487	H	-0.04719	-3.17764	0.829962
C	-0.91893	-0.15099	-0.50498	H	1.35435	-1.95265	-1.63162
C	-2.19648	0.029698	0.054444	H	-4.50292	2.319772	-0.07324
C	1.161993	-1.14213	-0.91925	H	-4.85202	0.568856	-0.00414
O	-2.86379	1.159348	-0.26188	H	-4.107	1.392097	1.401657
C	-4.15968	1.358237	0.308052	H	2.39608	-1.98476	0.604053
C	-0.14848	0.71074	-1.41628	H	1.396576	-0.10672	1.808416
O	1.042465	0.077959	-1.67927	H	3.514635	1.436706	0.228548
C	2.359612	-1.02681	0.059369	H	3.45918	2.657228	2.503011

C	2.146934	0.089308	1.044685	H	2.202316	3.292815	1.44826
C	2.773276	1.266002	1.006445	H	1.777163	2.125495	2.71809
O	-0.41304	1.781377	-1.91801	H	3.695104	-1.5615	-1.25666
C	2.535836	2.387898	1.973227				

Table S28. Cartesian coordinates of **3b-14**.

C	3.007155	1.404376	0.009738	O	-2.26533	1.757478	0.483011
C	2.03343	2.373797	-0.25371	H	4.029993	1.717405	0.176644
C	0.694715	2.041643	-0.4822	H	2.336589	3.41608	-0.28497
C	0.359007	0.693096	-0.43361	H	-0.04075	2.805151	-0.70798
C	1.315027	-0.2889	-0.18134	H	-1.42809	0.20738	-1.5757
C	2.662219	0.044674	0.0463	H	5.332218	-0.13931	-0.36411
C	-0.97274	0.014364	-0.59962	H	5.01145	-0.00982	1.393776
O	3.524142	-0.96613	0.279609	H	5.400636	-1.59575	0.668773
C	4.89833	-0.64231	0.506743	H	-1.53664	0.07279	1.467387
C	0.662017	-1.60567	-0.22994	H	-3.14068	-1.4917	0.253374
O	-0.66891	-1.39736	-0.51622	H	-4.58099	1.21142	0.276395
C	-1.99917	0.362074	0.51079	H	-6.46352	-0.46824	0.819321
C	-3.2717	-0.41294	0.316221	H	-6.25504	-0.34601	-0.92334
C	-4.48558	0.129587	0.209058	H	-5.55888	-1.72695	-0.049
O	1.101112	-2.72366	-0.07605	H	-1.54436	2.203214	0.953672
C	-5.75104	-0.65017	0.00373				

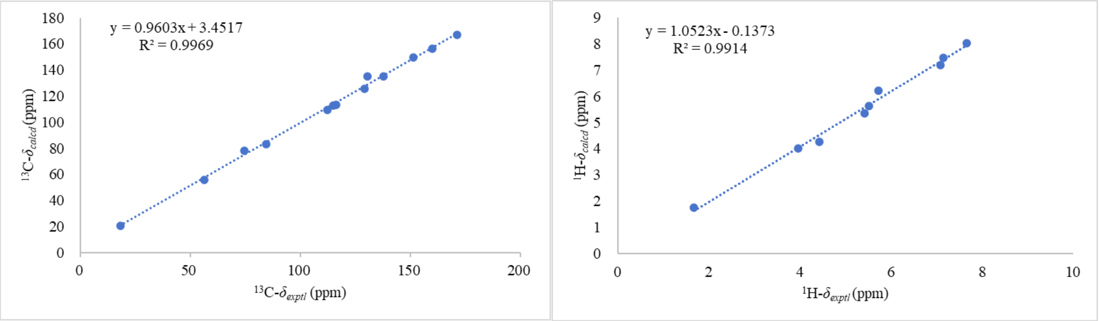

Figure S79. Linear regression analysis between the experimental and calculated NMR chemical shifts of **3a**.

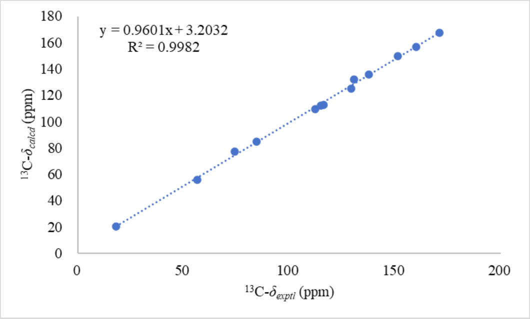

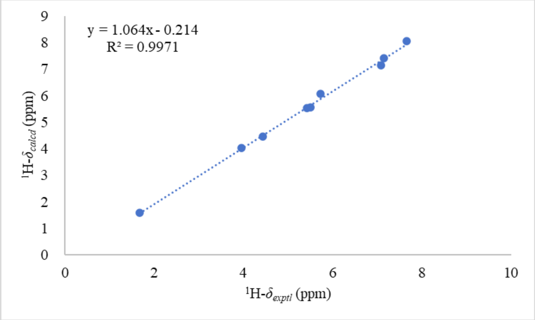

Figure S80. Linear regression analysis between the experimental and calculated NMR chemical shifts of **3b**.

Table S29. The experimental and calculated ^{13}C NMR chemical shifts of **3**, **3a** and **3b** and the MAE and CMAE analysis of **3a** and **3b**.

Number	Explt-**3**	Calcd-**3a**	Calcd-**3b**
1	171.1	166.9	167.4
3	84.5	83.3	84.7
3a	151.3	149.4	149.6
4	116.2	113.6	112.5
5	137.6	135.2	135.5
6	112.3	109.6	109.4
7	159.9	156.5	156.7
7a	115.0	112.6	112.3
8	74.2	78.2	77.5
9	129.2	125.8	125.3
10	130.5	134.9	132.1
11	17.9	20.8	20.5
12	56.3	56.0	56.0
R^2		0.9969	0.9982
MAE		2.7	2.4
CMAE		1.7	1.4

Table S30. The experimental and calculated ^{1}H NMR chemical shifts of **3**, **3a** and **3b** and the MAE and CMAE analysis of **3a** and **3b**.

Number	Explt-**3**	Calcd-**3a**	Calcd-**3b**
3	5.42	5.35	5.53
4	7.15	7.47	7.40
5	7.66	8.01	8.05
6	7.09	7.19	7.15
8	4.43	4.27	4.46
9	5.51	5.64	5.56

10	5.73	6.22	6.07
11	1.68	1.76	1.58
12	3.96	4.02	4.02
R^2		0.9914	0.9971
MAE		0.20	0.15
CMAE		0.13	0.07

Table S31. The DP4+ probabilities of **3a**-**3b**.

Functional	Solvent	Basis Set	Type of Data
mPW1PW91	PCM	6-31+G(d,p)	Shielding Tensors

	Isomer **3a**	Isomer **3b**
sDP4+ (H data)	0.48%	99.52%
sDP4+ (C data)	4.63%	95.37%
sDP4+ (all data)	0.02%	99.98%
uDP4+ (H data)	50.84%	49.16%
uDP4+ (C data)	34.25%	65.75%
uDP4+ (all data)	35.01%	64.99%
DP4+ (H data)	0.50%	99.50%
DP4+ (C data)	2.47%	97.53%
DP4+ (all data)	0.01%	99.99%

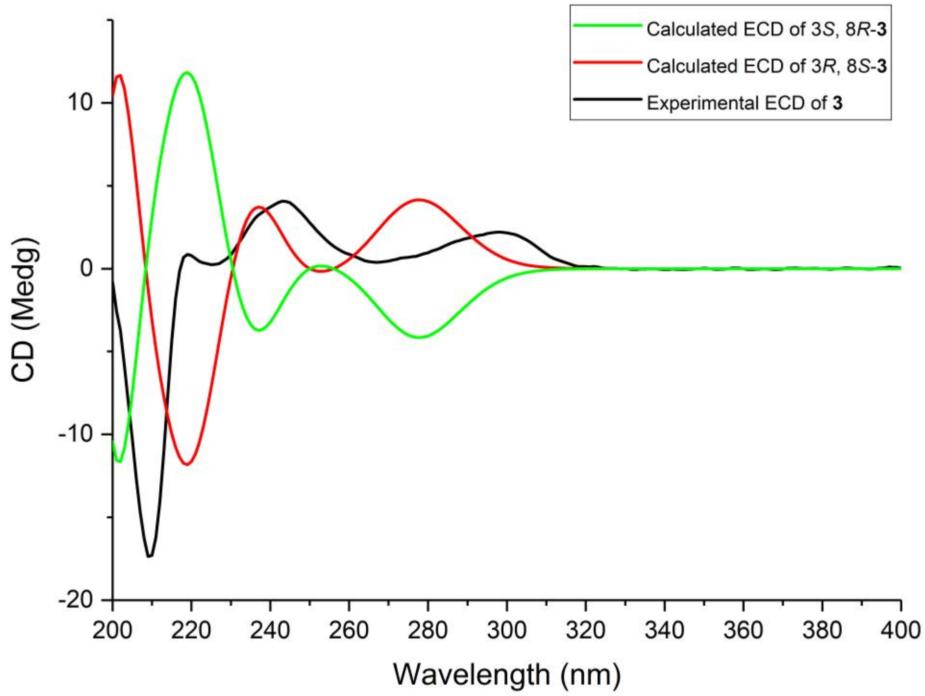

Figure S81. Comparison between the experimental ECD and the calculated ECD spectra for compound **3**.

The ITS sequence of *A. subversicolor* CYH-17

CCGTGACTACCTAACACTGTTGCTTCGGCGGGGAGCCCCCTTCCCGGGGGCGAGCCGCCG
GGGACTACTGAACTTCATGCCTGAGAGTGATGCAGTCTGAGTCTGAATATAAAATCAGTC
AAAACTTTCAACAATGGATCTCTTGGTTCCGGCATCGATGAAGAACGCAGCGAACTGCGA
TAAGTAATGTGAATTGCAGAATTCAGTGAATCATCGAGTCTTTGAACGCACATTGCGCCC
CCTGGCATTCCGGGGGGCATGCCTGTCCGAGCGTCATTGCTGCCCATCAAGCCCGGCTTG
TGTGTTGGGTCGTCGTCCCCCCCGGGGGACGGGCCCGAAAGGCAGCGGCGGCACCGTGTC
CGGTCCTCGAGCGTATGGGGCTTTGTCACCCGCTCGATTAGGGCCGGCCGGGCGCCAGCC
GACGTCTCCAACCATTTTTCTTCAGGTTGACCTCGGATCAGGTAGGGATACCCGCTGAAC
TTAAGCATA

Table S32. The antibacterial activity of the compounds.

MIC / μM	Strain					
	B. subtilis	*E. profundum*	*E. faecalis*	*A. baumannii*	*S.aureus*	MRSA
**1**	-	100	-	-	-	-
**10**	0.1	25	25	50	25	12.5
**11**	-	100	-	-	-	-
ciprofloxacin	0.03	0.2	0.8	0.4	0.8	1.6

Table S33. The α-glucosidase inhibitory activity and antioxidant activity of the compounds.

IC_{50} / μM	α-glucosidase inhibitory activity	antioxidant activity
**1**	37.2 ± 0.7	-
**10**	127.8 ± 6.3	48.9 ± 9.8
**11**	101.3 ± 5.0	-
**13**	66.3 ± 2.7	-

Acarbose	0.01 ± 0.00	-
Ascorbic acid	-	16.7 ± 0.7

References

1. Grimme, S. Exploration of chemical compound, conformer, and reaction space with meta-dynamics simulations based on tight-binding quantum chemical calculations. *J. Chem. Theory Comput.* **2019**, 15 (5), 2847-2862.

2. Lu, T. Molclus program, Version 1.9.9.9, http://www.keinsci.com/research/molclus.html (accessed Mar-6, **2022**).

3 Bruhn, T.; Schaumlöffel, A.; Hemberger, Y.; Pecitelli, G. SpecDis version 1.71, Berlin, Germany, **2017**, https:/specdis-software.jimdo.com.
